# Regulation of brain cognitive states through auditory, gustatory, and olfactory stimulation with wearable monitoring

**DOI:** 10.1038/s41598-023-37829-z

**Published:** 2023-08-08

**Authors:** Hamid Fekri Azgomi, Luciano R. F. Branco, Md. Rafiul Amin, Saman Khazaei, Rose T. Faghih

**Affiliations:** 1 Electrical and Computer Engineering Department, University of Houston, Houston, TX 77004 USA; 2 Department of Neurological Surgery, University of California San Francisco, San Francisco, CA 94143 USA; 3 Biomedical Engineering Department, University of Houston, Houston, TX 77004 USA; 4 Department of Biomedical Engineering, New York University, New York, New York, 10003 USA

**Keywords:** Neuroscience, Psychology, Biomarkers, Engineering, Mathematics and computing

## Abstract

Inspired by advances in wearable technologies, we design and perform human-subject experiments. We aim to investigate the effects of applying safe actuation (i.e., auditory, gustatory, and olfactory) for the purpose of regulating cognitive arousal and enhancing the performance states. In two proposed experiments, subjects are asked to perform a working memory experiment called *n*-back tasks. Next, we incorporate listening to different types of music, drinking coffee, and smelling perfume as safe actuators. We employ signal processing methods to seamlessly infer participants’ brain cognitive states. The results demonstrate the effectiveness of the proposed safe actuation in regulating the arousal state and enhancing performance levels. Employing only wearable devices for human monitoring and using safe actuation intervention are the key components of the proposed experiments. Our dataset fills the existing gap of the lack of publicly available datasets for the self-management of internal brain states using wearable devices and safe everyday actuators. This dataset enables further machine learning and system identification investigations to facilitate future smart work environments. This would lead us to the ultimate idea of developing practical automated personalized closed-loop architectures for managing internal brain states and enhancing the quality of life.

## Introduction

Any activity might be a source of cognitive stress. Stress in workplaces^1^ and cognitive load while learning at schools^2^ are considered examples of such occasions that might cause cognitive stress in humans. Additionally, to reach enhanced productivity and retain it, it is crucial to elevate cognitive arousal levels and prevent low engagement^3–5^. According to the Yerkes–Dodson law from psychology, one’s cognitive performance levels change as a function of their cognitive arousal state by following an inverse-U-shaped relationship^5,6^. Hence, cognitive performance can be maximized by maintaining the internal arousal state in an optimal range^7,8^. Besides cognitive performance enhancement, over the last few years, a growing interest in human emotion regulation arises in various areas such as education^9,10^, neural rehabilitation^11,12^, physiological disorder treatments^13^, and brain–computer interfaces^14,15^. Thus, it is crucial to regulate the arousal state and keep it within the optimal range^16^. In this research, we design and perform human-subject experiments to analyze the effects of safe actuation (i.e., auditory, gustatory, and olfactory) on participants’ cognitive performance state and explore their arousal state fluctuations.

To investigate the relationship between cognitive performance and internal arousal states, we aim to analyze the changes in cognitive arousal state while under cognitive load^17–19^. As the internal arousal state is a hidden state, we approach this problem indirectly. In response to the presence of cognitive stress stimuli, similar to any other internal or external stimuli, the brain reacts in multiple ways. Monitoring brain signals with Electroencephalography (EEG)^20–23^ or functional Near-Infrared Spectroscopy (fNIRS)^24,25^ methods would shed light on how the brain would respond to those environmental stimuli. In addition to the direct changes in the human brain, there are also fluctuations in other physiological signals such as heart rate (HR), blood volume pulses (BVP), and electrodermal activity (EDA)^26^ that carry important information regarding the internal arousal state. With recent advances in wearable technologies, there exist fascinating and unique opportunities to investigate human brain responses in a more practical way. Compared to research-grade technologies that are more expensive and precise in sensing, wearable devices are designed to be seamlessly integrated into everyday life and smart work environments^26–30^. Low-cost and portability features are the most remarkable characteristics that make the wearable technologies more attractive in the field of emotion recognition and human performance^31–33^.

In this study, we focus on employing only wearable devices for monitoring physiological responses. We propose to use Empatica E4 wristbands^34^ and a muse headband^35^ to collect data from human subjects while exposing them to cognitive stress tasks. The Empatica E4 wristband employs noninvasive sensors to collect multiple physiological signals (i.e., EDA, BVP, Photoplethysmography (PPG), 3-axis accelerometer data, and skin temperature). Additionally, we employ a muse headband to directly record brain electrical activity with a noninvasive EEG method^36,37^. Compared to other research-grade devices that collect EEG signals from the entire scalp, and are not practical in daily life, a muse headband collects EEG signals from four channels^38^.

In the present research, to induce cognitive stress, we propose to employ a well-studied working memory experiment called *n*-back tasks^39^. We design and perform *n*-back experiments to investigate the brain responses while under cognitive load^40–42^. In *n*-back tasks, the system represents a sequence of stimuli, each followed by a consistent fixation^43^. The participants are asked to recall if the stimulus they observe is the same as the one they were shown during the *n*-th step before. Higher values of *n* will result in more difficult tasks. This study also seeks to investigate the effects of using safe actuation in influencing physiological responses and enhancing cognitive performance state.

There are multiple studies discussing how listening to different kinds of music (as a safe actuation) might affect humans’ internal states^44–46^. Researchers in^47^ performed an experiment to show the efficiency of listening to music in improving imagery in the context of sports skills. By collecting physiological data such as EDA and HR, they have demonstrated the effectiveness of listening to music in enhancing the performance index. Lehmann et al. examined the effectiveness of background music in improving learning outcomes^48^. Half of the subjects are asked to perform a memory task in silence while the other half are asked to listen to two pop songs^48^. The results further support their hypothesis about the positive role of music in improving working memory capabilities^49^. In a similar study, Du et al. analyzed the effects of high and low-arousal music on neural responses by using eye blinks extracted from the recorded EEG data^49^. While there exists rich literature verifying the positive impacts of listening to music on the human brain state, there is still a lack of experimental studies that computationally evaluate their effects on the human performance state and explore their impacts in changing physiological data collected via wearable technologies. To this end, in experiment 1, we propose to use music as a safe intervention for regulating the internal arousal state and enhancing the performance state.

In addition to listening to music, there exist other types of safe actuation that would influence human cognitive behavior. Caffeine intake and olfactory stimulants are examples of safe actuation that would be effective in brain state regulation. The caffeine involved in coffee is in the class of central nervous system (CNS) stimulants. Organic molecular methylxanthine in caffeine causes an increase in energy metabolism and influences cognitive function. These positive impacts are widely discussed in several studies on humans and animals^50–57^. McLellan et al. performed a comprehensive review of multiple studies verifying the effects of caffeine in enhancing alertness, attention, and reaction time^58^. Souissi et al. demonstrated how caffeine ingestion is effective in enhancing cognitive and physical performance^59^. They used reaction time and “number cancellation” tests to analyze cognitive performance^59^. Researchers in^60^ designed an experiment and analyzed the effects of coffee intake on the brain’s electrical activity. Saifudinova et al. collect and analyze EEG signals before and after taking coffee. In a recent study by Sargent et al., they performed experiments and collected EEG and EDA data from subjects while performing daily tasks in a naturalistic work environment^61^. While participants are in an office-type environment, they were provided with hot beverages. In a similar study, researchers in^62^ designed and performed experiments to investigate the effects of hot tea and coffee on cognitive performance. During the experiment, they collected EDA and fNIRS data from the subjects. To explore the effects of coffee on brain-computer interfaces, Meng et al. performed an experiment and analyzed EEG signals from the subjects who are asked to drink coffee^63^. In a separate study, Fine et al. also verified the effects of caffeine in improving cognitive performance and reducing fatigue^54^.

In past decades, the effects of olfactory stimulation have also been explored by multiple researchers^64^. Examples of these studies are analyzing the effects of smelling perfumes on lung function and exercise performance^65^, pain management^66,67^, and alleviating psychological effects in women’s menopausal symptoms^68^. Porcherot *et al.* designed and performed experiments to investigate changes in emotions in response to smelling fragrances^69^. Similar to any stimulation, to analyze the effects of olfactory stimulation, researchers proposed to collect multiple physiological signals such as cardiac and electrodermal activity^70^, EEG recording^71^, galvanic skin response^72^, heartbeat^73^, and fNIRS^74^. Saeki et al. investigated the effects of inhaling favorite fragrances for relieving pricking pain^75^. They used electrical stimulation to cause pain and measured skin conductance levels^75^. The results verify their hypothesis about the positive influence of fragrances to alleviate pain. They also discuss the possibility of the effectiveness of aromatherapy in chronic pain relief^76^. Onuma et al. conducted similar research and recorded brain activity from the frontal region and explored how smelling fragrances would affect that area^77^. They concluded a positive relationship between activity associated with the right region of the brain and induced impression^77^. Moss et al. performed experiments for evaluating the effects of different aromas in modulating cognitive performance^78^. They showed that peppermint has significant potential to enhance cognitive mood. The results of these studies validate the effects of smelling fragrances on changes in individuals’ psychological and physiological conditions. Inspired by these findings, we propose to explore the effects of drinking coffee and smelling perfumes on cognitive performance and arousal states for potential future use in related closed-loop applications. In experiment 2, we design and perform human-subject experiments to analytically investigate the effects of this safe actuation on participants’ cognitive performance state while using only wearable technologies for physiological signals monitoring.

We hypothesize that using safe actuators would improve the cognitive performance state and influence the cognitive arousal state. With the goal of making the findings in this research more applicable in everyday life, we only use wearable technologies for our investigation. To infer an individual’s internal arousal state, we model and estimate the hidden state by utilizing well-established computational tools. To this end, we analyze EDA data measured by the Empatica E4. While the main function of sweat gland activation is body thermoregulation, it also carries important information regarding an individual’s internal arousal state^17,18,79^. In response to internal and external stimuli, the human brain employs the autonomic nervous system to adjust sweat gland secretions^80^. Accordingly, skin surface conductivity, which is measured by electrodes placed on the Empatica wristbands, provides information about brain peripheral signals. By performing deconvolution algorithms and inferring underlying neural impulses^81,82^, we employ state-space representations and point process algorithms to model and estimate internal arousal state^83,84^. Scholars have shown that the state-space representation is a suitable tool for capturing internal arousal state in response to the changes in skin conductance signal^85,86^. As another measure, we also collect EEG signals to directly monitor brain activity and study its functionalities in the proposed experiments.

In summary, we analyze the impacts of listening to different kinds of music while subjects perform cognitive tasks and are under cognitive load in experiment 1. In experiment 2, we propose to explore the effects of drinking coffee and smelling fragrances as safe actuation for closing the loop. To explore the effectiveness of this safe actuation in enhancing the performance state, we record the correct/incorrect responses as well as their reaction time while performing *n*-back tasks. Next, we employ a similar state-space approach to model the cognitive performance state. We take correct/incorrect response and reaction times as binary and continuous observations and utilize a Bayesian filtering method to estimate the hidden performance state. The obtained data in these experiments will give us the insight required to confirm our hypothesis on the effectiveness of safe actuation while closing the loop in a systematic way. In this study, we have analyzed a comprehensive set of data, including behavioral measurements (i.e., correct/incorrect responses and reaction times) and physiological measures (i.e., EDA and EEG). However, we posit that a more thorough exploration of the remaining physiological data holds significant potential for advancing our understanding of human neurophysiology. Moreover, this expanded investigation will afford valuable insights into the effects of safe actuation on modulating cognitive states at the neural level.

### Contribution

While there exist multiple studies discussing the impacts of safe actuation in regulating the brain’s cognitive states, a systematic approach is still required for implementing them in real-world environments. The proposed experiments in this study are the first attempts to explore cognitive brain state regulation using safe actuation by utilizing only wearable technologies. Employing commercially available wearable devices (i.e., Empatica E4 and muse headband) along with safe actuation (i.e., music, perfume, and coffee) make this research applicable in real-world settings. The goal is to demonstrate how safe actuation intervention would enhance the cognitive performance state and regulate the arousal state. The insights gained from this study could provide tremendous illumination for practical future uses in closed-loop regulation of internal brain states. We make a comprehensive physiological and behavioral dataset of working memory *n*-back experiments available to the research community. The resulting dataset in these human-in-the-loop experiments has also great potential to be further investigated for modeling the dynamics of the proposed safe actuation in modulating internal brain states and would enhance our understanding of human neurophysiology.

## Results

In this section, we demonstrate the results of two experiments. In both experiments, subjects are asked to perform working memory *n*-back tasks. In experiment 1, we analyzed the effects of listening to music on their physiological data and their cognitive performance levels. In experiment 2, the effects of smelling perfume and drinking coffee on their cognitive performance levels are analyzed. Hence, we first present the raw physiological data collected from the wearable devices (i.e., Empatica wristband and muse headband). To evaluate the effects of safe actuation while performing memory tasks, we employ correct/incorrect responses and their reaction time to estimate the cognitive performance state. Moreover, the results of analyzing the skin conductance signals and estimating the internal arousal state are presented for each experiment. In what follows, we present one sample subject in each experiment. The results associated with the rest of the subjects are presented in the supplementary material document.

**Figure 1:**
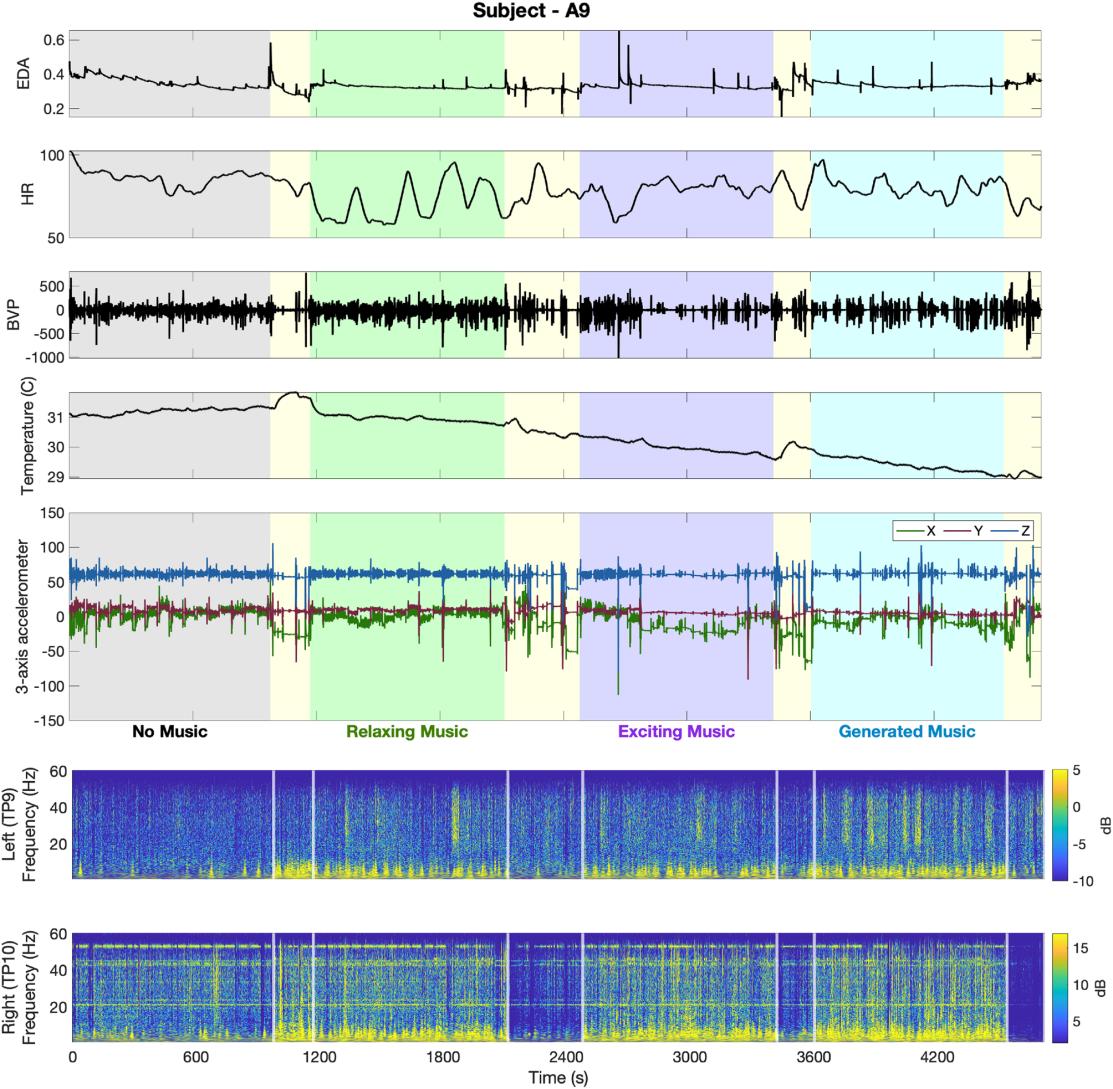
Raw physiological data collected via Empatica E4 Wristband and Muse Headband (Subject—A9, Experiment 1). In the top panel, sub-panels in turn represent the EDA, HR, BVP, body temperature, and 3-axis accelerometer data. The grey, green, purple, and blue background colors in turn represent the results associated with no music, relaxing music, exciting music, and newly generated relaxing music sessions, respectively. Yellow backgrounds are associated with rest time periods. Sub-panels in the bottom panel depict spectrogram representations of EEG signals recorded from the left and right temporoparietal areas of the brain (i.e., TP9 and TP10). White vertical lines separate the memory tasks from rest times.

**Figure 2:**
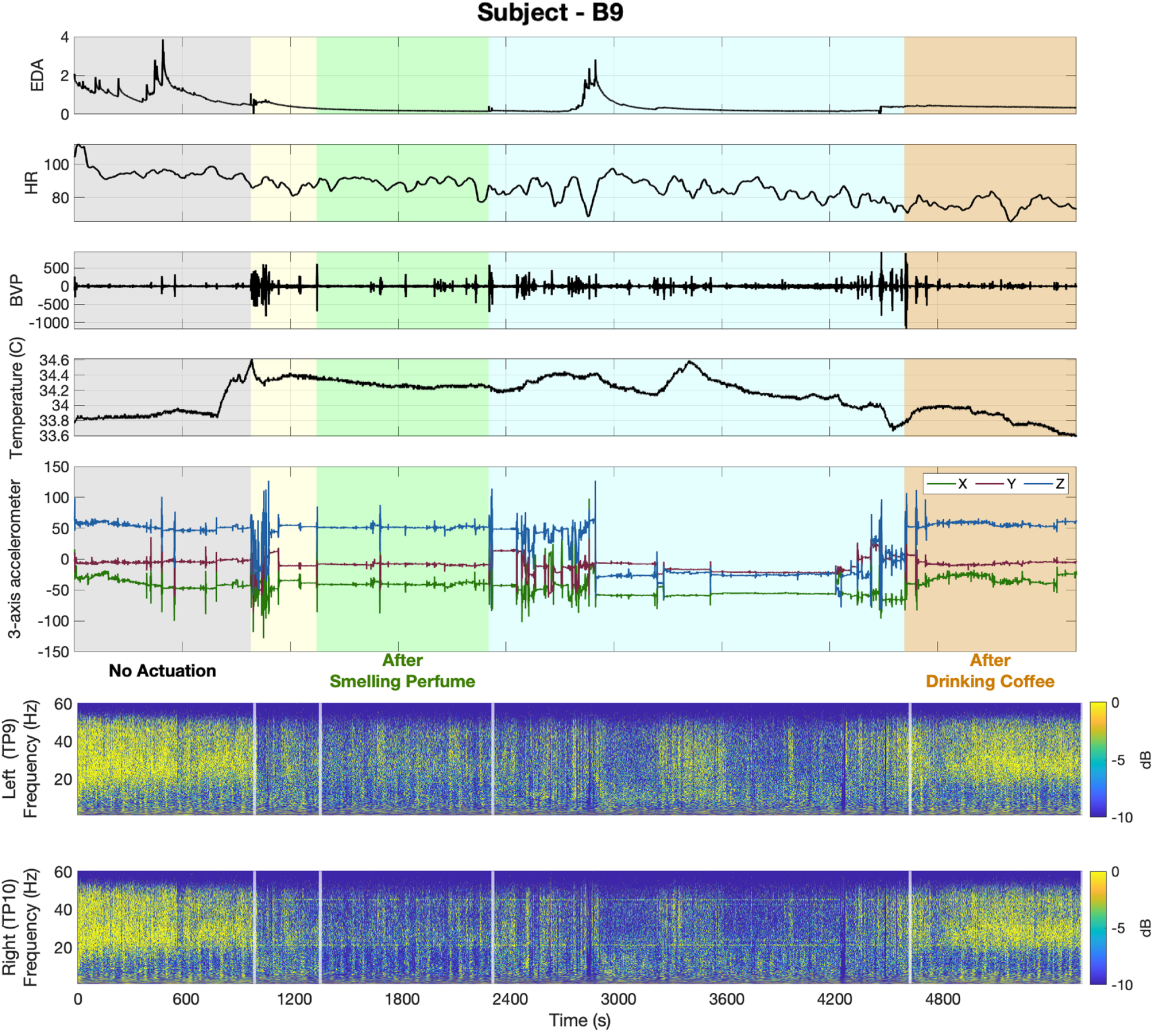
Raw physiological data collected via Empatica E4 Wristband and Muse Headband (Subject—B9, Experiment 2). In the top panel, sub-panels in turn represent the EDA, HR, BVP, body temperature, and 3-axis accelerometer data. The grey, green, and rust background colors in turn represent the results associated with no actuation, smelling perfume, and drinking coffee sessions, respectively. Yellow and blue background colors are associated with rest time periods for smelling perfume and drinking coffee, respectively. Sub-panels in the bottom panel depict spectrogram representations of EEG signals recorded from the left and right temporoparietal areas of the brain (i.e., TP9 and TP10). White vertical lines separate the memory tasks from rest times.

The recorded physiological data in experiments 1 and 2 are presented in Figs. 1 and 2. In Fig. 1 the grey, green, purple, and blue background colors in turn represent the results associated with no music, relaxing music, exciting music, and newly generated relaxing music sessions, respectively. The yellow backgrounds are associated with rest time periods. In experiment 1, after each session, participants are asked to sit and relax for three minutes. The rest time after the second session, which is in the middle of the experiment, was set to 6 min. In Fig. 2, the grey, green, and rust background colors in turn represent the results associated with no actuation, smelling perfume, and drinking coffee sessions, respectively. The yellow background is associated with the six minutes they have to smell their choice of fragrance as the olfactory stimuli. The blue background implies the time participants have to drink the coffee.

As illustrated in the top panels of Figs. 1 and 2, Empatica provides us with electrodermal activity (EDA) (i.e., measured as skin conductance signal), heart rate (HR) (i.e., derived from the interval between successive heartbeats), blood volume pulses (BVP), body temperature, and 3-axis accelerometer data. The muse headband used in these experiments provides us with electroencephalogram (EEG) recordings from four channels. The spectrogram analysis of two temporal channels (i.e., left (TP9) and right (TP10))^87,88^ are presented in the bottom panel of Figs. 1 and 2. To generate these spectrogram representations from the raw EEG signals, we followed the steps presented in the data analysis section.

**Figure 3:**
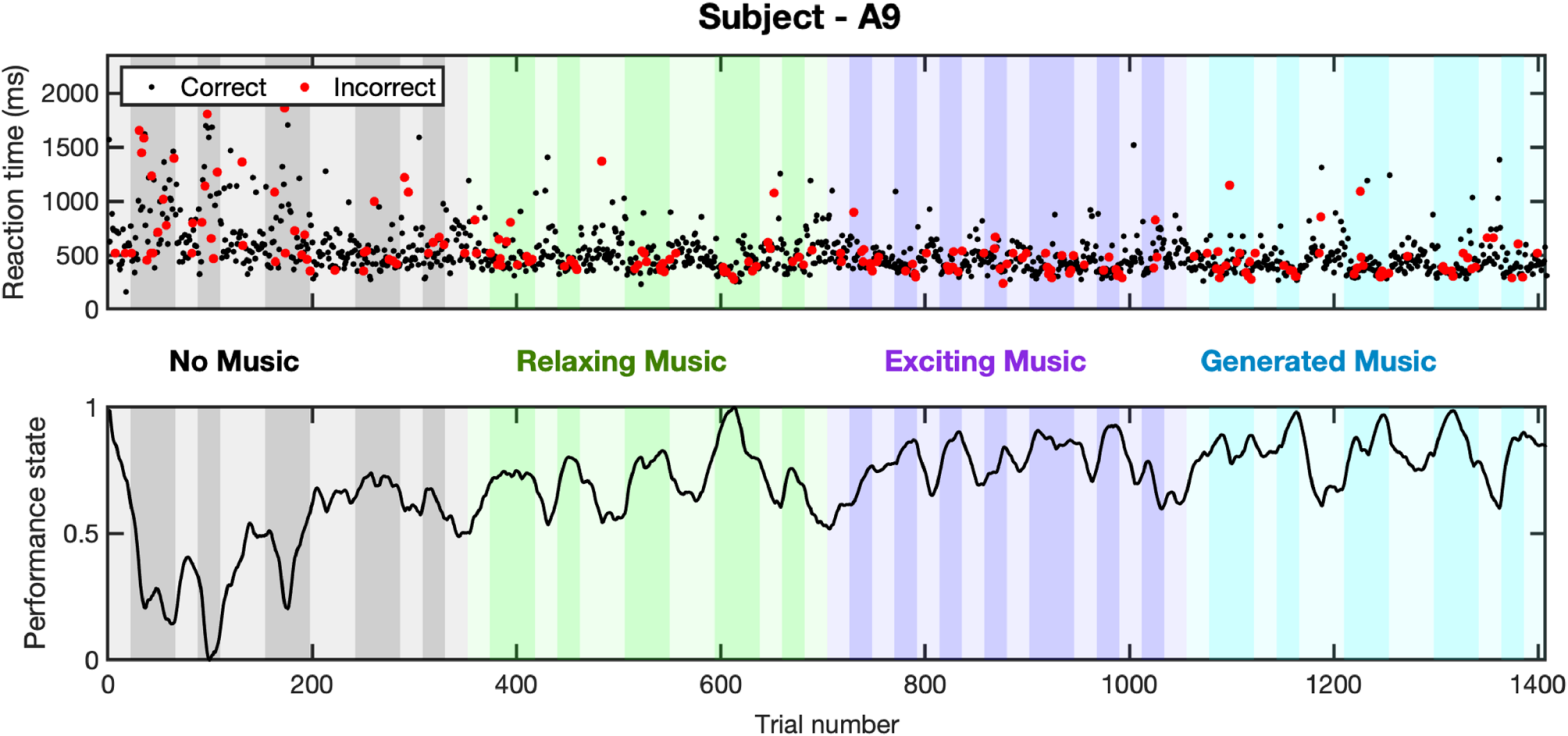
Cognitive performance results (Subject—A9, Experiment 1). The top panel shows the reaction time along with correct (black) and incorrect (red) responses. The bottom panel shows cognitive performance state estimates. The grey, green, purple, and blue background colors in turn represent the results associated with no music, relaxing music, exciting music, and newly generated relaxing music sessions, respectively. Bright and dark backgrounds present 1-back and 3-back tasks, respectively.

**Figure 4:**
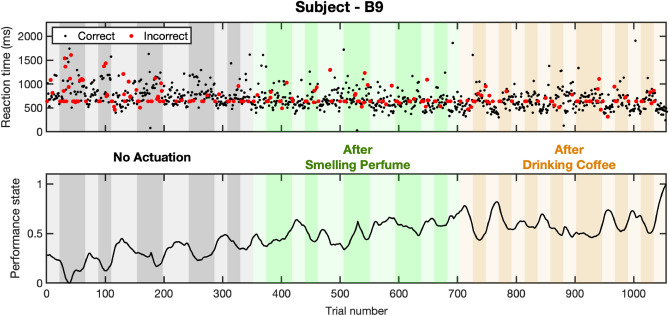
Cognitive performance results (Subject—B9, Experiment 2). The top panel shows the reaction time along with correct (black) and incorrect (red) responses. The bottom panel shows cognitive performance state estimates. The grey, green, and rust background colors in turn represent the results associated with no actuation, smelling perfume, and drinking coffee sessions, respectively. Bright and dark backgrounds present 1-back and 3-back tasks, respectively.

**Figure 5:**
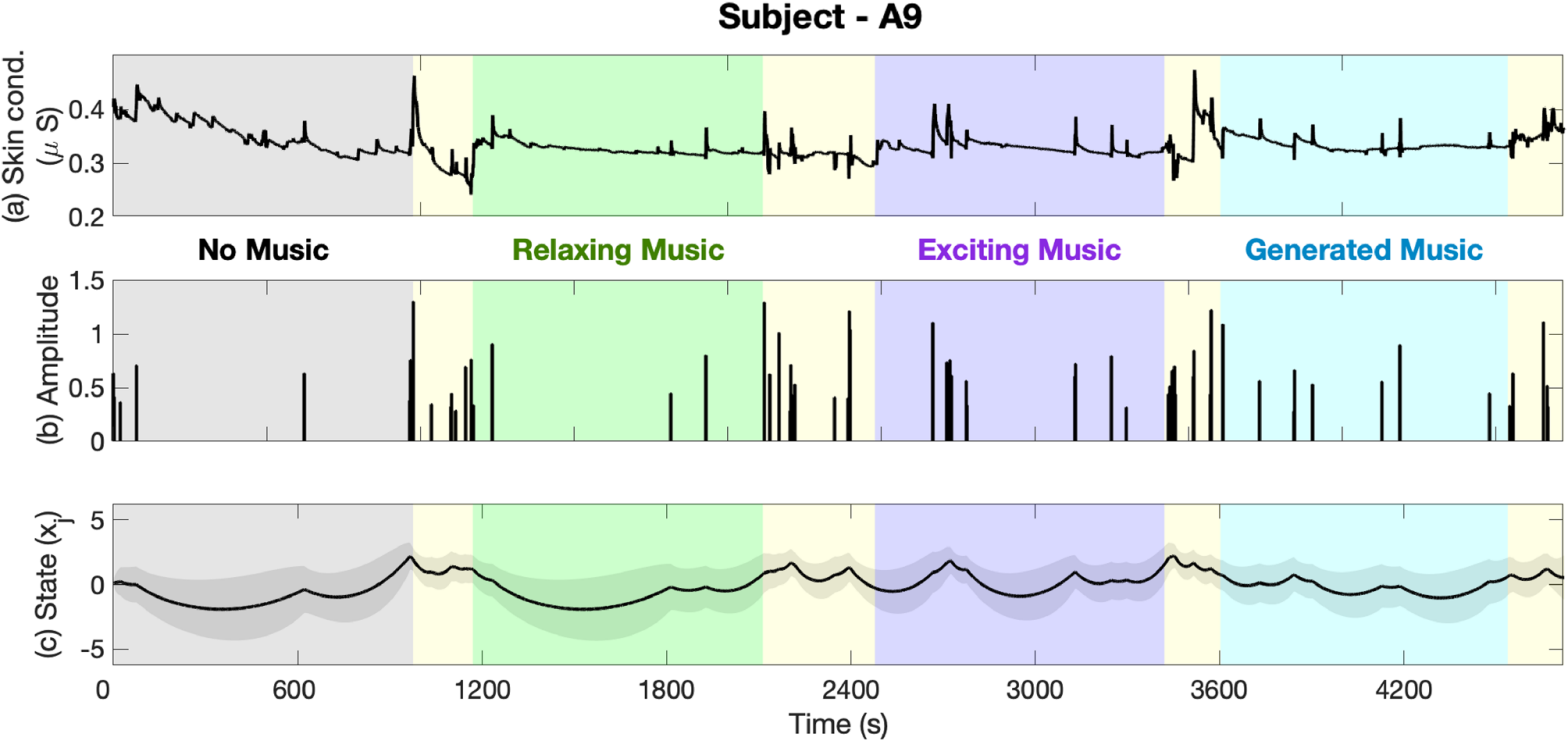
Cognitive arousal results (Subject—A9, Experiment 1). Panels from the top show in turn the skin conductance signal, underlying neural impulses, and estimated cognitive arousal state. The grey, green, purple, and blue background colors represent the results associated with no music, relaxing music, exciting music, and newly generated relaxing music sessions, respectively. Yellow backgrounds are associated with rest time periods.

**Figure 6:**
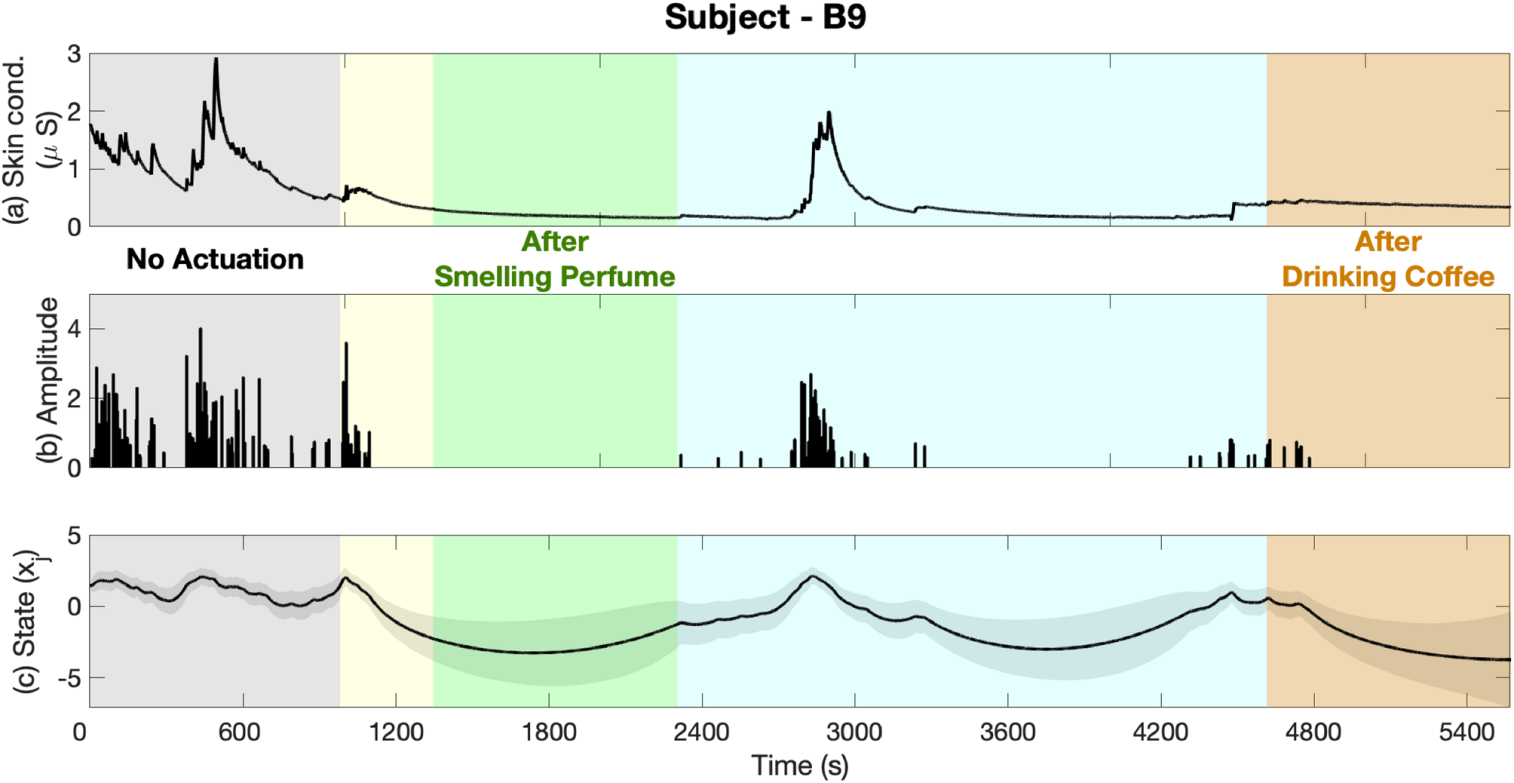
Cognitive arousal results (Subject—B9, Experiment 2). Panels from the top show in turn the skin conductance signal, underlying neural impulses, and estimated cognitive arousal state. The grey, green, and rust background colors represent the results associated with no actuation, smelling perfume, and drinking coffee sessions, respectively. Yellow and blue background colors are associated with rest time periods for smelling perfume and drinking coffee, respectively.

The collected correct/incorrect responses and their reaction times while performing *n*-back experiments, along with the estimated cognitive performance state are presented in Figs. 3 and 4. In each figure, the first panel shows the reaction time along with correct (black) and incorrect (red) responses. The second panel shows cognitive performance state estimates. Bright and dark backgrounds represent 1-back and 3-back tasks, respectively. The results of estimating the internal arousal state based on analyzing skin conductance signals are presented in Figs. 5 and 6. As presented in these figures, panels from the top show in turn the skin conductance signal, underlying neural impulses, and estimated cognitive arousal state. Different background colors are associated with different sessions of each experiment.

## Discussion

**Figure 7:**
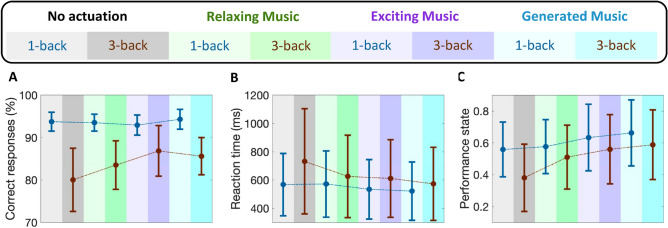
Performance analysis of all participants (Experiment 1). Box-plots in each panel show in turn **(A)** correct response percentage, **(B)** reaction time, and **(C)** estimated cognitive performance state for all participants.

**Figure 8:**
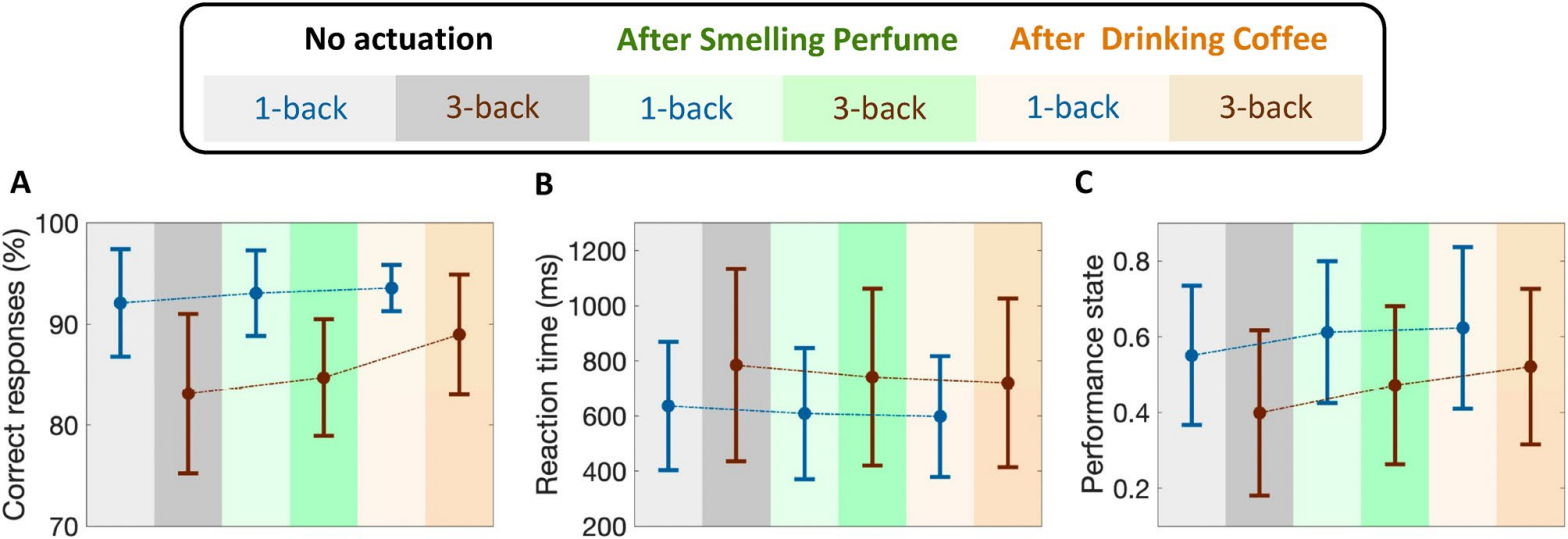
Performance analysis of all participants (Experiment 2). Box-plots in each panel show in turn **(A)** correct response percentage, **(B)** reaction time, and **(C)** estimated cognitive performance state for all participants.

As one of the very first attempts in brain state regulation with safe actuation using only wearable technologies, we designed and performed human-subject experiments. We designed two sets of memory-related *n*-back experiments and proposed to take safe actuation (i.e., listening to different music tracks in experiment 1, smelling perfumes, and drinking coffee in experiment 2) for the purpose of brain state regulation. We examined the effectiveness of this safe actuation on regulating brain states and enhancing cognitive performance state (see Figs. 7, 8). Employing only wearable devices to record physiological signals makes the findings of this research applicable in real life. As a result of these experiments (Figs. 1, 2), we collected multiple physiological data using wearable devices. In terms of physiological signal processing, our aim is to (1) analyze the EDA signal collected via the Empatica E4 and derive the internal arousal state, and (2) investigate changes in EEG signal power, and (3) study their connection with cognitive performance state. The remaining of physiological data presented in the top panels of Figs. 1 and 2 (i.e., HR, BVP, temperature, and 3-axis acceleration) were not analyzed in this research but hold great potential for future investigation and analysis. In what follows, we discuss the results of each experiment. Next, we elaborate on general findings and discuss the challenges.

Participants in experiment 1 were asked to listen to different kinds of music while performing memory tasks. Fluctuations in their physiological data (Fig. 1) and performance state (Fig. 3) demonstrate important information regarding the effects of listening to music while under cognitive load. Analyzing the results of all participants, we observe an enhancement in their cognitive performance state while listening to music. The primary aim of using music as an intervention during cognitive tasks is to investigate their impact in (i) controlling the performance state, (ii) keeping it within the desired range, and (iii) preventing the subjects from feeling bored and unengaged. Moreover, our results indicate that listening to music led to an increase in the performance state and helped them better concentrate on memory tasks. To further explain these results, we perform multiple analyses to compare the participants’ performance levels within different sessions (i.e., baseline with no music and sessions while listening to different music). As it is presented in Fig. 7, listening to relaxing music elevates the average levels of performance state in 3-back tasks from 0.38 to 0.51. This is equal to 34% enhancement in the estimated performance levels while listening to relaxing music (p < 0.001). The observed enhancement in the levels of performance state is because of either receiving more correct responses or faster reaction times. The utilized Bayesian filter to estimate the modeled performance stat incorporates this information (i.e., correct/incorrect responses and reaction times) and results in an estimate of the cognitive performance state. As depicted in panels A and B of Fig. 7, listening to relaxing music improved the rate of correct responses by 3.5% within faster reaction times (i.e., 17.1% faster reaction times (p < 0.001) compared to the baseline with no music played). It should be also noted that listening to relaxing music slightly improved subjects’ performance levels in 1-back tasks (p > 0.4). In the third session, subjects showed higher improvements while listening to exciting music. Listening to exciting music increases the average levels of performance state from 0.55 and 0.38 to 0.63 (p < 0.001) and 0.56 (p < 0.001) in 1-back and 3-back tasks, respectively. These enhancements were because of receiving more correct responses and/or faster reaction times. Listening to exciting music resulted in 5.9% and 16.5% faster reaction times in 1-back and 3-back tasks, respectively. Interestingly, these faster reaction times in 3-back tasks are achieved with receiving a significant increase in the number of correct responses (i.e., a 6.8% increase compared to the baseline with no music played (p < 0.001)). In 1-back and 3-back tasks by 5.9% and by 3.5% within faster reaction times (i.e., 17.1% faster reaction times (p < 0.001) compared to the baseline with no music played).

In addition to these familiar types of music, we also played newly generated relaxing music in the fourth session of experiment 1, which they had never heard before. These newly generated relaxing music tracks were created by using deep learning approaches. In response to listening to this new music, participants showed higher levels of performance state (i.e., reaching 0.66 (p < 0.001) and 0.58 (p < 0.001) performance levels in 1-back and 3-back tasks, respectively). The main objective of incorporating the newly generated music into the study was to evaluate its potential positive impact on participants’ cognitive performance levels. Our findings suggest that despite the music being tailored to the subject’s preferences, its novelty factor might have contributed to the observed cognitive enhancements. However, to fully comprehend the potential benefits of new artificial intelligence (AI)-generated music on cognitive performance, further research is necessary. This could entail exploring the specific music features that lead to cognitive improvements, as well as investigating the potential for personalized AI-generated music to enhance cognitive function.

In experiment 2, where the participants were asked to smell perfume and drink coffee as the safe actuation, we observe an enhancement in average levels of performance state. Similar to experiment 1, the proposed safe actuation not only prevents the participants from feeling bored and unengaged but also leads the subjects to achieve higher performance levels. Here, we perform corresponding analyses to compare the performance levels in the baseline session with those in sessions with safe actuated conditions. As it is illustrated in Fig. 8, smelling perfume has increased the average levels of performance state from 0.55 and 0.39 to 0.61 (p < 0.001) and 0.47 (p < 0.001) in 1-back and 3-back tasks, respectively. Drinking coffee has also increased the average levels of performance state from 0.55 and 0.39 in 1-back and 3-back tasks to 0.62 and 0.52, respectively. This enhancement in the levels of estimated performance state is due to receiving more correct responses with faster reaction times. To further demonstrate the effects of olfactory stimulation and caffeine intake in improving brain cognitive performance levels, we compare their impacts on the number of correct responses and reaction times. As presented in Fig. 8, smelling perfumes improves the average rate of correct responses by 0.9% (p < 0.01) and 1.6% (p < 0.001) in 1-back and 3-back tasks, respectively. These improvements in correct responses are achieved with 4.4% (p < 0.01) and 5.4% (p < 0.01) enhancements in reaction times in 1-back and 3-back tasks, respectively. Similarly, in response to drinking coffee, subjects showed 1.4% (p < 0.05) and 5.8% (p < 0.001) enhancement in the rate of correct responses in 1-back and 3-back tasks, respectively. After Drinking coffee, more correct responses are achieved within faster reaction times (i.e., 6.1% (p < 0.01) and 8.6% (p < 0.01) improvement in 1-back and 3-back tasks, respectively). These comparisons further demonstrate how this safe actuation has improved subjects’ performance state by both reducing reaction times and receiving more correct responses. Therefore, one may conclude that the subjects’ cognitive performance state while conducting memory-related tasks would be improved after smelling perfume or drinking coffee.

**Figure 9:**
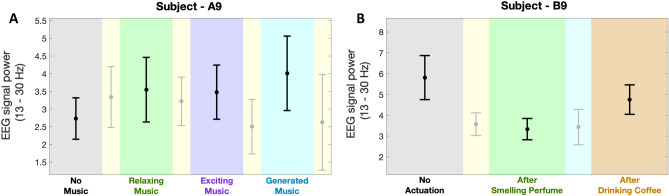
EEG power variation in beta band frequency. Panels **A** and **B** show in turn the average levels of EEG power in the beta frequency band for the subject—A9 (in experiment 1) and the subject—B9 (in experiment 2).

**Figure 10:**
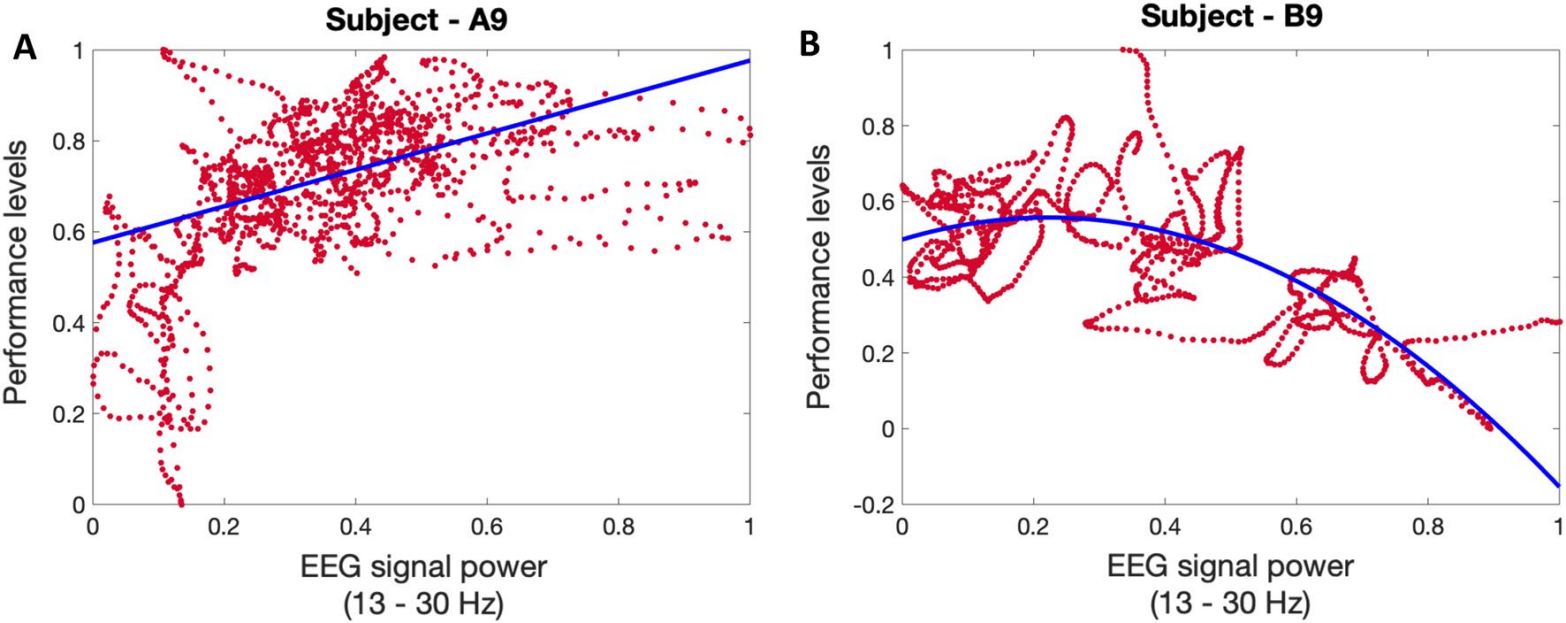
EEG beta band power-performance relationship. Panels **A** and **B** show in turn the relationship between the averaged levels of beta band power in the EEG signal and the normalized levels of estimated cognitive performance state of the subject—A9 (in experiment 1) and subject—B9 (in experiment 2). The blue line in each figure shows the fitted line to the actual data shown in red.

In addition to the positive impacts of the proposed safe actuation on enhancing the subjects’ performance state, we also observe different sorts of modulation in human physiological data. As these physiological data are collected only using wearable devices, further studying the aforementioned fluctuations would make these devices along with the proposed algorithms more applicable in everyday life. For instance, changes in EEG signals collected via muse headband would also provide us with insight regarding the impacts of safe actuation in brain activities. By prepossessing the EEG signal (see “Methods” section) and performing a continuous wavelet transform, we analyze the fluctuation in EEG signal power. As shown in the bottom panel of Fig. 1 (i.e., results of subject A9 in experiment 1), significant activation of brain beta frequency bands (13–30 Hz), during the *n*-back experiments compared to the rest periods, could be an indicator of the high levels of cognitive engagement. To further analyze these results, we compared the average levels of brain signal power in the beta frequency bands. As shown in panel A of Fig. 9, we observe a 29.9% increase in beta band power while listening to relaxing music and a 27.3% increase while listening to exciting music (p < 0.001). Interestingly, the increase in beta power was even greater, at 46.8%, while listening to newly generated music (p < 0.001). These higher levels of beta band power have also resulted in faster reaction times and an increase in correct response rates (as shown in the top and bottom panels of Fig. 3). Statistical analysis revealed an important 42.5% increase in the average levels of estimated performance state compared to the baseline with no actuation (p < 0.001). So, we might conclude that listening to different kinds of music leads to this increase in the beta band power and could eventually be a reason for the observed enhancement in the cognitive performance state in this specific subject (Fig. 3). As seen in the bottom panel of Fig. 3, listening to different kinds of music has also led to an improvement in cognitive performance levels. Therefore, it can be inferred that the increase in beta band power while listening to music is an indicator of an elevated cognitive performance state in the subject. To further explore the connection between the beta band power and the subject’s performance state, we present their relationship in panel A of Fig. 10. The results demonstrate a linear-shaped relationship between the estimated performance levels and the beta band power calculated from the EEG signals. This implies that achieving the maximum levels in the performance state is correlated with the increase in beta band power. These findings are consistent with previous studies reporting higher levels of alpha and beta band activity in the brain’s temporal region during cognitive tasks^87,88^. Additionally, the higher levels of beta band power during the actuated periods (while listening to relaxing music, exciting music, and newly generated music) may be attributed to the positive effects of safe actuation.

To further investigate the impacts of safe actuation utilized in experiment 2 (i.e., smelling perfume and drinking coffee) on the physiological signals, we conducted a similar analysis on their EEG signal. As shown in panel B of Fig. 9, subject B9 exhibited a different beta power synchronization compared to subject A9 in Panel A of Fig. 9 during no actuation periods. Subject B9 started with higher signal power in the baseline compared to subject A9. Smelling perfume resulted in a 42% drop in the beta power in the second session (p < 0.001). Drinking coffee increased beta power by 24%, which was higher than the second session (i.e., after smelling perfume) but relatively lower than the baseline with no actuation (% a8 lower compared to the baseline (p < 0.001)). Therefore, subject B9 experienced a lower level of beta power after smelling perfume and reached a moderate level after drinking coffee. These moderate levels of beta band power after drinking coffee led to faster reaction times and an increase in correct response rates (see top and bottom panels of Fig. 3). Statistical analysis indicated an important 115% increase in the average levels of estimated performance state compared to the baseline with no actuation (p < 0.001). Hence, we might conclude that drinking coffee for this subject led to modulation in the beta band power and could be a cause for the improvement in cognitive performance state (Fig. 3). Additionally, the higher levels of beta band power with no actuation could be attributed to the low levels of performance state in this subject. This finding supports the hypothesis that for achieving the highest performance levels, neither high levels of engagement nor a low level is desired^5,6^. Similar to the analysis conducted for experiment 1, we have explored the connection between the beta band power and the subject’s performance state in experiment 2. The relationship between them is presented in panel B of Fig. 10. The findings indicate that there exists an inverted U-shaped relationship between the estimated performance levels and the beta band power derived from the EEG signals. This implies that achieving the maximum levels in the performance state requires the modulation of beta band power within a moderate range. These results further demonstrate the need to investigate the impact of any actuation within a personalized framework.

**Figure 11:**
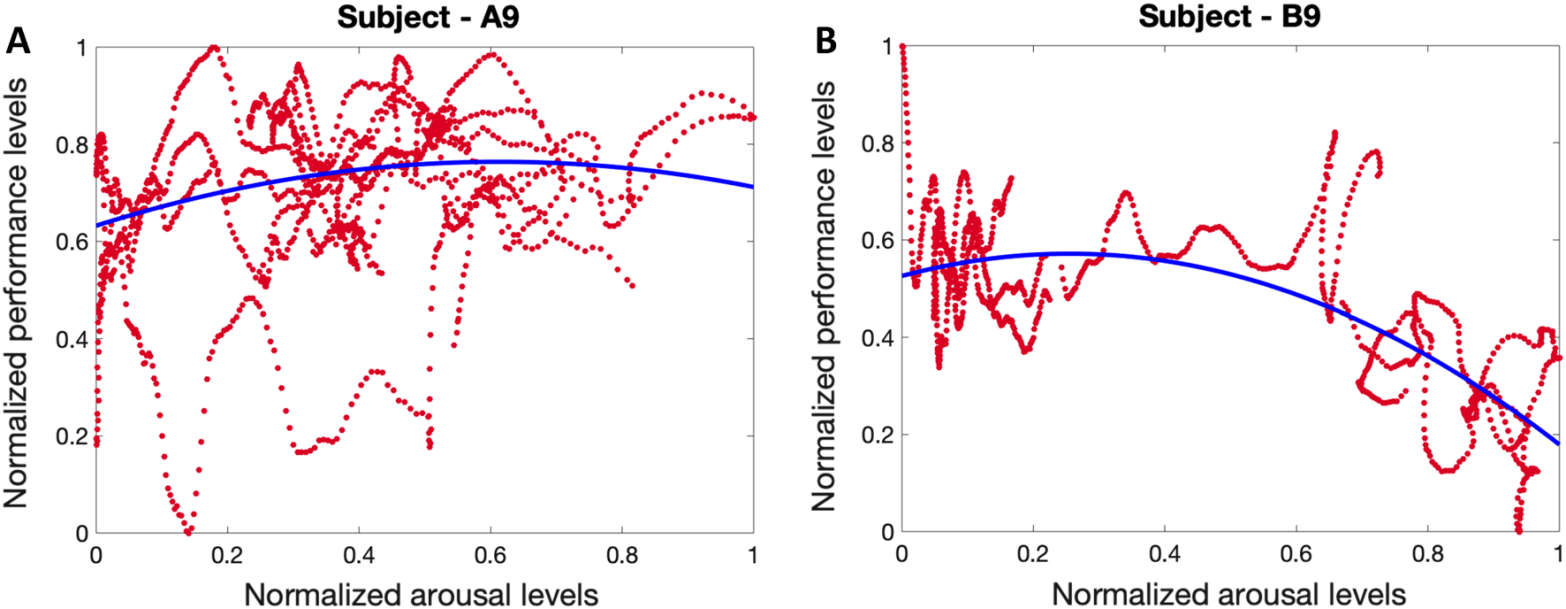
Arousal-performance relationship. Panels **A** and **B** show in turn the relationship between the normalized levels of estimated cognitive arousal and estimated cognitive performance state of Subject—A9 (in experiment 1) and Subject—B9 (in experiment 2). The blue curve in each figure shows the inverted U-shaped fitted to the actual data shown in red.

In addition to these changes in EEG signal recorded from the muse headband, we analyzed skin conductance signal (as a valid indicator of the internal arousal state^17,83,89–91^) collected by Empatica wristband. By utilizing skin conductance signals, applying a deconvolution algorithm to extract underlying neural impulses, and employing a state-space approach to relate the underlying neural impulses to the hidden arousal state, we estimated the internal arousal state (Fig. 5). As seen in the top panel of Fig. 5, although we observe more skin conductance responses in the raw signal in the first session (i.e., no music), the deconvolution algorithm recovers fewer arousal events (over sparsifies). This can be due to changes in the sweat dynamics as we move to the three sessions with music played in the background putting more emphasis on the last three-quarters of the data. This indicates the need for an adaptive deconvolution algorithm for real-world settings. Both Figs. 1 and 5 demonstrate that listening to all kinds of music modulated the subject’s skin conductance data and their estimated arousal state. We then sought to explore the relationship between the arousal state and the estimated performance state. As presented in panel A of Fig. 11, we examined the normalized levels of arousal and performance state in subject A9. The results reveal an inverted U-shaped relationship between these two cognitive states. This implies that to achieve maximum performance, it is important to maintain arousal levels within a moderate range.

In a similar manner, and to examine how this safe actuation impacts the subjects’ physiological data in experiment 2, we analyzed the arousal levels of subject B9. The selected subject shows a distinct pattern in their physiological signal modulation in response to the safe actuation (i.e., smelling perfume and drinking coffee). The data presented in both Figs. 2 and  6, indicate that subject B9 exhibited a decrease in skin conductance levels after smelling perfume, followed by an increase in levels after drinking coffee. As a result, changes in the estimated arousal state can be attributed to these observed alterations in skin conductance levels. As shown in the bottom panel of Fig. 6, smelling perfumes has resulted in a drop in estimated arousal levels, while drinking coffee has led to an elevation in the levels of estimated arousal state. As a final step, we explore the relationship between the arousal state and performance state in Fig. 11. As shown in panel B of Fig. 11, we see an inverted U-shaped relationship here. This suggests that optimal performance is achieved when arousal levels are modulated and kept within a moderate range. These person-specific insights are valuable for the development of closed-loop architectures aimed at improving performance and productivity. While an inverted U-shaped relationship between arousal and performance was observed in the example subjects (i.e., A9 in experiment 1 and B9 in experiment 2), it should be noted that some subjects exhibited different patterns (see Supplementary Information). This highlights the importance of a personalized framework in the design of closed-loop systems.

The performed analyses provide additional insights into how the implementation of safe actuation can modify the performance state of individuals by improving their reaction times and/or increasing the accuracy of their responses. It is also worth mentioning that these experiments lasted so long (i.e., more than 70 min in each experiment) and it is a possibility that without safe actuation (either listening to music, smelling perfume, or drinking coffee), the subjects’ performance state would be dropped due to tiredness. Therefore, we may conclude that the proposed safe actuation while performing memory-related tasks would enhance subjects’ productivity. In addition, one concern that could arise is that the improvement in cognitive performance levels may be due to the habituation effect. While this is a valid point, it should be considered that within each session we do not observe meaningful improvements that could be resulted from learning the tasks (Figs. 3, 4). To validate this hypothesis, a possible future direction of this research could be changing the sequence of sessions to further differentiate the effects of habituation versus the influence of applying safe actuation. It should also be noted that among the various n-back tasks, we specifically selected the 1-back and 3-back tasks for our study. Although it is possible to include 2-back tasks, we opted to focus on the 1-back and 3-back tasks to reduce the duration of the experiment. Additionally, the primary goal of experiments in this research was to examine the influence of safe actuation on cognitive performance. Therefore, we chose the 1-back and 3-back tasks as distinct and representative cases to better comprehend their dissimilarities. In designing experiment 1, we also included washout periods lasting 3 min after session one, and 3 and 6 min after session two and three, respectively. The duration of these periods is influenced by various factors such as the nature of the task, individual responses, and the characteristics of the music stimulus^92^. Importantly, when utilizing shorter intervals between music tracks, it is critical to recognize the potential for lingering effects from the previous session to persist into the subsequent one.

## Methods

**Figure 12:**
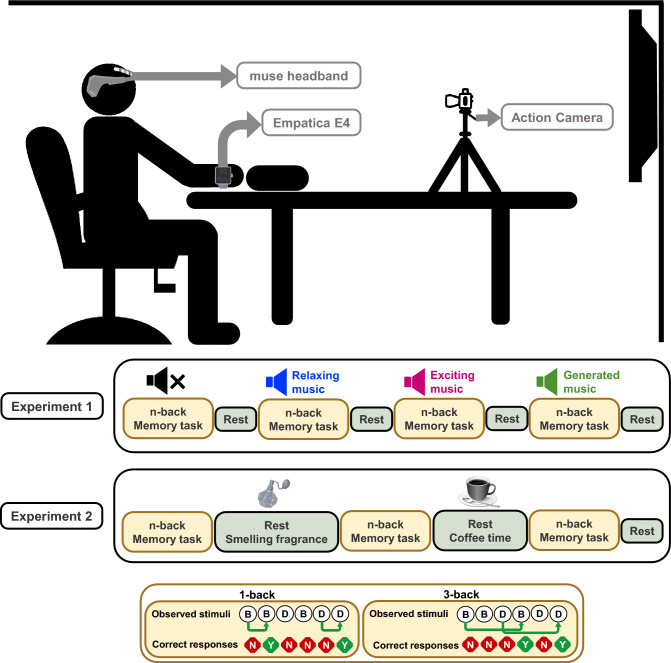
Experimental setup and summary. Panels demonstrate the experimental setup and experiments’ summary. During the experiment, the participant is seated comfortably on an armchair and wears a muse headband and one empatica E4 wristband on each hand. During the experiment, the subject looks at the screen to perform *n*-back memory-related tasks and we record facial activity with an action camera. In experiment 1, listening to music is used as the safe actuation. In experiment 2, smelling fragrances and drinking coffee are used as the safe actuation. The bottom panel describes the *n*-back tasks.

All experiments were performed in the Computational Medicine Lab (CML) at the University of Houston (see Fig. 12). During the experiment, the subject is seated comfortably on an armchair and wears a muse headband and Empatica E4 wristbands on both hands. During the experiment, the subject looks at the screen to perform memory-related *n*-back tasks. We also record facial activity with an action camera. All methods were performed according to the guidelines of the Declaration of Helsinki and the current ethical guidelines. All the experimental procedures and corresponding documents were approved by the institutional review board at the University of Houston, TX, USA (STUDY 00002490).

### Participants

This pilot study includes two sets of experiments. Subjects were recruited from the members of the University of Houston (i.e., students and postdocs) for these experiments. In Experiment 1, 17 participants (11 males, 6 females) were recruited in total. In Experiment 2, 13 participants (10 males, 3 females) were recruited in total. Participants were required to be at least 18 years old. All participants read and signed an informed consent document. All participants received gift cards as incentive compensation. They all received a base amount plus an additional incentive to further encourage them to fully focus on the tasks.

In analyzing participants’ data, seven participants were excluded due to the E-Prime program crashes ($N=1$) for collecting participants’ behavioral responses or the quality of wearable Empatica wristband data due to the motion artifacts and/or the lack of proper contact between the wearable’s electrodes and participants’ skin surface ($N=6$) in experiment 1. In experiment 2, the data of ($N=3$) participants were excluded similar to experiment 1. In the present study, we utilized 10 participants for each experiment and labeled the subjects accordingly (please see supplementary information). In Experiment 1, 10 subjects (6 males, 4 females), with a mean age of 29.2 (SD = 4.0), were included in total (i.e., subject A1—subject A10). In Experiment 2, 10 subjects (8 males, 2 females), with a mean age of 29.0 (SD = 3.8), were included in total (i.e., subject B1—subject B10).

### Equipment

We used two wearable Empatica E4 wristbands and a portable muse headband for EEG recording. Using the Empatica E4 wristbands, we collected electrodermal activity (EDA) (or skin conductance) that tracks the changes in skin conductivity using two metal electrodes, blood volume pulse (BVP), from which heart rate variability can be measured, using a photoplethysmography sensor, motion-based activity using a 3-axis accelerometer sensor, and skin temperature using infrared thermopile. Using the 2016 edition muse headband, we collected brain activity using four EEG sensors.

### Procedure

In this study, we propose to perform two sets of experiments for regulating cognitive arousal and cognitive performance states. As presented in Fig. 12, in the first experiment we aim to analyze the effects of listening to music in enhancing brain states. In the second experiment, we evaluate the effects of drinking coffee and smelling perfume in regulating brain cognitive states. In each experiment, we ask the subjects to perform memory-related *n*-back tasks.

To design experiments 1 and 2, we used E-Prime professional software (version 3.0) on a Dell Latitude 5580 DESKTOP-Q6TBA9H. Within E-Prime, E-Studio and E-Data Aid modules were used to design the presentation of multiple sessions of *n*-back tasks. To record the responses, we used Chronos. To have a more comfortable setting, we used a 50 inches LCD screen mounted on the wall within a 2-meter distance of the subjects (see Fig. 12). Participants were asked to sit in an armchair comfortably facing the screen with their dominant hand on the Chronos response device.

In the designed *n*-back experiments, subjects were shown trials of stimulus (500 ms) along with a plus sign for their response (1500 ms). Each session consisted of an instruction that lasted for 5 s and 16 trials each of which includes 22 stimuli. There were 10 s breaks in between trials and 20 s relaxation in between the 16 trials. The total duration of each session was 964 s (i.e., $16\times [5+(22\times 2)+10]+20 = 964$). To specify their response, participants had to press the target (green) vs non-target (red) buttons on Chronos. Before the start of the experiment, they were provided with instructions regarding the tasks and performed a couple of practice trials (i.e., one 1-back and one 3-back trial). In the 1-back task, the participants were asked to determine if the stimulus they saw is the same as they saw one step before. Conversely, in the 3-back task, they were asked to indicate if the one they saw is the same as they observed three steps before (see bottom sub-panel in Fig. 12). In session one of experiment 1, subjects perform *n*-back tasks with no music. In the second session, they are asked to repeat the tasks while listening to their choice of relaxing music. In the third session, they repeated the tasks while listening to their choice of exciting music. In the final session, they repeated the tasks while listening to the newly generated relaxing music. In what follows we illustrate the process of generating music based on their taste for relaxing music.

Music taste is a colloquial term to represent how different songs have distinct effects on each individual. Nowadays, there are multiple music genres and within each genre, there are various bands producing a variety of content, in accordance with a wide range of preferences from their audiences. Musical preference is a very subjective matter, which usually encodes distinct auditory stimulation responses in the brain^93^. Moreover, music has been used to improve clinician-rated depressive symptoms^94^, reduce stress levels and increase performance during exams^95,96^ and improve performance in non-complex cognitive tasks^97^. Artificially generated music is an interesting research topic that has the potential to automatically alter musical parameters and optimize the songs to achieve certain desired goals, such as relaxation, excitation, or concentration^98^. In this research, we employ deep learning neural networks to generate new songs based on the subject’s preference. More specifically, we use a long short-term memory (LSTM) neural network. LSTMs are capable of learning long-term and short-term dependencies and have been widely used in music generation^98,99^. The LSTM architecture is comprised of various interconnected cell blocks that transfer the cell’s hidden state to the next cell, after mathematical manipulations. Each cell block has a memory component that gets altered via the *Forget, Input, and Output* gates. The Forget gate is responsible for removing unwanted information from the cell state, the input gate selects relevant information to be stored in the cell’s state, and the output gate filters information for the next cell, all based on the input data and previous cell’s output. Each gate works by computing^99^

1 $$\begin{aligned} f_t&= \sigma (w_f [h_{t-1}, u_t ] + b_f), \end{aligned}$$

2 $$\begin{aligned} i_t&= \sigma (w_i [h_{t-1}, u_t ] + b_i), \end{aligned}$$

3 $$\begin{aligned} o_t&= \sigma (w_o [h_{t-1}, u_t ] + b_o), \end{aligned}$$

where $f_t$, $i_t$, $o_t$ represent forget, input, and output gates, respectively. $\sigma$ stands for a Sigmoid function, $w_x$ and $b_x$ are weights and biases for a gate *x* respectively. $h_{t-1}$ represents the output of the previous cell block at time-step $t-1$, $u_t$ is the input at current time-step.

To generate the music in this study, we employ a neural network with three LSTM layers in succession, with a recurrent dropout parameter set to 0.3. With this parameter, at every update, a percentage of the input is dropped, preventing over-fitting. Next, a batch normalization layer is added, followed by a fully connected layer, and an activation layer with a rectified linear activation function (ReLU). Subsequently, another batch normalization layer is added, a dropout layer, and a fully connected layer before the final activation layer with a “SoftMax” function. The “SoftMax” is a generalized logistic function. Finally, the loss metric is calculated during the training phase with a categorical cross-entropy function.

The input songs for the training phase of the neural network need to be in textual format. For this, we use the musical instrument digital interface (MIDI) format as there are plenty of songs available online^100^. This text-based musical format carries instructions on how to play the song, such as notation, pitch, and tempo. With this format, the neural network is easily trained on the n-th sequence of notes of songs from a dataset. Once training is complete, each prediction of a future note considers the n-th previous notes and the neural network would be capable of generating new songs with a similar structure. We trained 3 separate networks in 3 different datasets of MIDI songs, obtained from^100^. The musical genres chosen were, (1) classical music with songs from Ludwig van Beethoven, Johann Sebastian Bach, and Frédéric François Chopin; (2) fantasy music with video-game songs such as the various Final Fantasy and Mario theme songs; and (3) jazz music including songs from Frank Sinatra and various other authors. Prior to the start of the experiment, samples of these types of music are played for the subjects and we asked them to choose their favorite one. Within the experiment, newly generated music based on their selection is played for them in the last session. After each session, they sit and relax for three minutes (the relaxation time after the second session was 6 min). The entire duration of the experiment is about 80 min.

In experiment 2, in the first session, they perform *n*-back tasks with no actuation. Before the second session, they are asked to smell their choice of fragrance. They have 6 min to apply this actuation. Next, they are asked to repeat the *n*-back tasks. In the third session, we aimed to investigate the effects of drinking coffee as the actuation. They were provided with their regular coffee and were asked to sit down and drink their coffee during a 30-min break while resting. Next, they repeat performing the memory tasks. The entire duration of the experiment is about 90 min.

### Data analysis

We perform multiple types of analyses to evaluate our hypothesis about the effectiveness of safe actuation in regulating brain states. These include physiological signal processing and evaluating cognitive performance levels. To evaluate cognitive performance state, we follow the methodology presented in^101,102^ to model latent performance state^6^. Using a systematic approach, we relate the cognitive performance state to the subjects’ correct/incorrect responses and reaction times in the *n*-back experiments. We next estimate the latent performance state. Moreover, we employ physiological measurements and use a state-space representation to model the internal arousal state. To estimate the internal arousal state, we analyze the skin conductance signal collected via Empatica E4 wristbands. By applying a deconvolution algorithm and inferring underlying neural impulses, we establish a marked point process Bayesian filter to estimate hidden cognitive arousal state^83,91^. For the purpose of statistical analysis, we conducted a one-way analysis of variance (ANOVA) to test for differences in behavioral data, physiological signals, and all derived metrics.

#### Prepossessing of EEG recordings and spectrogram representation

Due to the low spatial resolution of the EEG signal, we first filter the raw EEG signal^22,36^. To this end, we start with raw EEG signal and perform high-pass filtering above 1 Hz and low-pass filtering below 50 Hz, similarly to previous studies^103^. Next, the signal is down-sampled from 256 to 128 Hz to improve computational efficiency. Then, we run “clean_artifacts” from EEGLAB toolbox, version 2021^104^. This function is based on burst criteria on the EEG signal. To calculate the spectrogram of the cleaned EEG signal, we use built-in constant-Q nonstationary Gabor transform (*cqt*) MATLAB function.

#### Cognitive performance analysis

Pursuing the approach presented in^101^, we model the cognitive performance state as a first-order state-space system

4 $$\begin{aligned} z_{k+1} = \rho z_k + w_k, \end{aligned}$$

where $z_k$ is the hidden performance state, $v_k\sim {\mathscr {N}}(0,\sigma ^2_{w})$ represents the process noise, $\rho$ is the unknown coefficient; *k* stands for the trial number during each experiments. Assigning one binary observation (correct/incorrect response at $k$th trial) and one continuous observation (reaction time of the corresponding trial)^101^, we form the observation model

5 $$\begin{aligned} I_k&= \log (t_k) = \alpha _0 + \alpha _1 z_k + \delta _k, \end{aligned}$$

where $\delta _k\sim {\mathscr {N}}(0,\sigma ^2_{\delta })$, $t_k$ displays the reaction time at each trial; $\alpha _0$ and $\alpha _1$ are the unknown parameters. The binary response is assumed to follow a Bernoulli distribution with the probability mass function $p_k^{m_k}(1 - p_k)^{1 - m_k}$ where $p_k$ stand for the probability of receiving a response (i.e., $P(m_k = 1)$). To relate the performance state to the probability of having a correct response, we apply the same Sigmoid transform function. Therefore,

6 $$\begin{aligned} p_k&= \frac{1}{1 + e^{-(z_k + \mu)}}. \end{aligned}$$

The constant term $\mu$ can be evaluated by $\mu \approx \log \Big (\frac{p_0}{1 - p_0}\Big)$ where $p_0$ is the average probability of having a correct response over the experiment. Utilizing an expectation maximization (EM) approach, we estimate unknown parameters $\theta _P = \{\rho ,\sigma ^2_w,\alpha _0,\alpha _1,\sigma ^2_{\delta }\}$ as well as the performance state $z_k$. The E-step formulation consists of the following prediction and update steps.

*Prediction:*

7 $$\begin{aligned} z_{k|k-1}= & {} \rho z_{k-1|k-1}, \end{aligned}$$

8 $$\begin{aligned} s^2_{k|k-1}= & {} \rho ^2s^2_{k-1|k-1} + \sigma ^2_w. \end{aligned}$$

*Update:*

9 $$\begin{aligned} z_{k|k} &= z_{k|k-1} + \frac{s^2_{k|k-1}}{\alpha _1^2 s^2_{k|k-1} + \sigma ^2_{\delta }}\bigg [\sigma ^2_{\delta }(m_k-p_{k|k}) ]+ \alpha _1(I_k - \alpha _0-\alpha _1z_{k|k-1})\bigg ], \end{aligned}$$

10 $$\begin{aligned} s^2_{k|k}= & {} \bigg [\frac{1}{s^2_{k|k-1}} + p_{k|k}(1-p_{k|k})+\frac{\alpha _1^2}{\sigma ^2_{\delta }}\bigg ]^{-1}. \end{aligned}$$

To achieve smoother results, we perform the following smoothing steps

11 $$\begin{aligned} B_{k}&= \rho \frac{s^2_{k|k}}{s^2_{k+1|k}}, \end{aligned}$$

12 $$\begin{aligned} z_{k|K}&= z_{k|k}+B_{k}(z_{k+1|K}-z_{k+1|k}), \end{aligned}$$

13 $$\begin{aligned} s^2_{k|K}&= s^2_{k|k}+B^2_{k}(s^2_{k+1|K}-s^2_{k+1|k}). \end{aligned}$$

The expected values of $z_{k}^2$, and $z_{k}z_{k-1}$ can be derived as,

14 $$\begin{aligned} \mathbb {E}[z_{k}^2]&= z_{k|K}^2 + s^2_{k|K}, \end{aligned}$$

15 $$\begin{aligned} \mathbb {E}[z_{k+1}z_{k}]&= z_{k+1|K}z_{k|K} + B_{k}s^2_{k+1|K}. \end{aligned}$$

At the M-step, the expected log-likelihood function can be formulated as

16 $$\begin{aligned} Q_2&= \sum _{k=1}^K \mathbb {E}[m_k(\mu +z_k)-\log (1+e^{\mu + z_k})]\nonumber \\&\quad + \frac{-K}{2}\log (2\pi \sigma ^2_{\delta })-\sum _{k =1}^K\frac{\mathbb {E}\bigg [(I_k-\alpha _0-\alpha _1z_k)^2\bigg ]}{2\sigma _{\delta }^2} + \frac{-K}{2}\log (2\pi \sigma ^2_{w})-\sum _{k=1}^{K}\frac{\mathbb {E}\bigg [(z_k-z_{k-1})^2\bigg ]}{2\sigma _{w}^2}. \end{aligned}$$

Consequently, in response to the correct/incorrect responses and the subject’s reaction time, the cognitive performance state can be obtained.

#### Cognitive arousal analysis via EDA

While the main purpose of EDA or skin conductivity is the body’s thermoregulation, it carries important information about the internal cognitive arousal state. The human brain employs the autonomic nervous system to handle sweat gland activation and response to internal and external stimuli. The skin conductance signal consists of two components: fast varying phasic and slow varying tonic^105^. By performing a cvxEDA approach we first separate phasic and tonic parts. By performing a deconvolution algorithm, similar to the ones presented in^18,79,82,85^, we obtain the underlying neural impulses. Next, we employ a state-space approach to relate the internal arousal state to the changes in SCR events. Since the internal cognitive arousal state is not directly measured, we utilize a marked pint process Bayesian-type filter to estimate the hidden arousal state^83,106^. In what follows, we review the steps.

In this study, we utilize the approach presented in^85^. SCR measurement as a function of time can be thought of as the summation of a slow varying (tonic) component and a fast varying (phasic) component. The SCR signal can be represented by combining these three components as

17 $$\begin{aligned} y(t) = y_p(t)+y_s(t)+\nu (t), \end{aligned}$$

where *y*(*t*), $y_{p}(t)$, $y_{s}(t)$, and $\nu (t)$ represent the SCR signal, phasic component, tonic component, and noise process, respectively. The phasic responses can be written as the convolution operation between the autonomic nervous system activation *u*(*t*) and the phasic response $h_{1,\varvec{\tau }}(t)$, i.e. $y_p(t) = h_{1,\varvec{\tau }}(t) * u(t)$. The phasic impulse response $h_{\varvec{\tau }}(t)$ can be written as^85^

18 $$\begin{aligned} h_{\varvec{\tau }}(t) = {\left\{ \begin{array}{ll} \frac{1}{\tau _r-\tau _d} \Big (e^{- \frac{t}{\tau _r}} - e^{-\frac{t}{\tau _d}}\Big)&{};\quad \text {if}\ t\ge 0 \\ 0&{};\quad \text {otherwise} \end{array}\right. }. \end{aligned}$$

Here, $\tau _r$ and $\tau _d$ are the rise time and decay time of a skin conductance response. On the other hand, the autonomic nervous system activation can be modeled as the weighted shifted sum of the delta functions.

If SCR is periodically sampled with a period of $T_y$ for *M* measurements, we can write the discrete observation equation as follows, i.e., $u(t) = \sum _{i=0}^{N-1}u_i \delta (t-i T_u)$. Here, *N* is the number of impulses in the input and $T_u$ is the sampling frequency of the input

19 $$\begin{aligned} y[k] = y_p(kT_y)+y_s(kT_y)+ \nu [k], \end{aligned}$$

where $k \in \{1, 2, \cdots , M\}$ represents the $k^{\text {th}}$ measurement with sampling frequency of $T_y$. One should note that here, $T_d = N T_u = M T_y$ is the sampled signal duration. Here, $\nu [k]$ represents the discretized measurement errors. We model $\nu [k]$ as a zero-mean independent and identically distributed (i.i.d) Gaussian random variable. We write the discrete model for *y*[*k*] based on the tonic and phasic modeling

20 $$\begin{aligned} y[k] = \underbrace{h_{0,\varvec{\tau }}[k]y_{p_0}}_{\text {initial condition}} + \underbrace{{\textbf {h}}_{1,\varvec{\tau }}[k] \mathrm {{\textbf {u}}}}_{\text {phasic}} + \underbrace{{\textbf {h}}_{2}[k] {\textbf {q}}}_{\text {tonic}}+ \nu {[k]}, \end{aligned}$$

where $h_{0,\varvec{\tau }}[k] = e^{- \frac{kT_y}{\tau _d}}$, ${\textbf {h}}_{1,\varvec{\tau }}[k] =\big [ h_{1,\varvec{\tau }}(kT_y) \; h_{1,\varvec{\tau }}(kT_y - T_u) \; \cdots \; h_{1,\varvec{\tau }}(T_u) \; \underbrace{0 \; \cdots \; 0}_{N - \frac{kT_y}{T_u}}\big ]^\top$, ${\textbf {h}}_{2}[k] =\big [ h_{2}(kT_y+\Lambda _{s})\;\; h_{2}(kT_y) \;\; h_{2}(kT_y - \Lambda _{s}) \;\; \cdots \;\; h_{2}(kT_y-(P-1)\Lambda _{s})\big ]^\top$; $\varvec{\textrm{u}} = [u_{1}\quad u_{2}\quad \cdots \quad u_{N}]^\top$ represents a sparse vector containing all the amplitudes of the impulses in the autonomic nervous system activation model over the entire signal duration and ${\textbf {q}} =[q_1 \quad q_2 \quad \cdots \quad q_N]^\top$ represents all the coefficients of the cubic B-spline basis functions and $y_{p_0} = y_{p}(0)$. Here $h_2(t)$ represents the cubic B-spline basis functions and ${\textbf {h}}_2[k]$ is the discretized version of a shifted cubic-spline basis function. Here the knot size for the cubic B-spline basis functions is selected similar to^85^. The overall vector matrix form becomes

21 $$\begin{aligned} \varvec{\textrm{y}} = \underbrace{{\textbf {H}}_{0,\varvec{\tau }} y_{p_0}+ {\textbf {H}}_{1,\varvec{\tau }}{} {\textbf {u}}}_{\text {phasic}}+ \underbrace{{\textbf {H}}_{2}\mathrm {{\textbf {q}}}}_{\text {tonic}}+\mathrm {\varvec{\nu }}, \end{aligned}$$

where ${\textbf {y}} = [ y[1]\quad y[2]\quad \cdots \quad y[M]]^\top$, ${\textbf {H}}_{0,\varvec{\tau }} = [h_{0,\varvec{\tau }}[1]\quad h_{0,\varvec{\tau }}[2]\quad \cdots \quad h_{0,\varvec{\tau }}[M]^\top$, ${\textbf {H}}_{1,\varvec{\tau }} = [{\textbf {h}}_{1,\varvec{\tau }}[1]\quad {\textbf {h}}_{1,\varvec{\tau }}[2]\quad \cdots \quad {\textbf {h}}_{1,\varvec{\tau }}[M]]^\top$, ${\textbf {H}}_{2} = [{\textbf {h}}_{2}[1]\quad {\textbf {h}}_{2}[2]\quad \cdots \quad {\textbf {h}}_{2}[M]]^\top$, and $\mathrm {\varvec{\nu }} = [\nu _{1}\quad \nu _{2}\quad \cdots \quad \nu _{M}]^\top$. Here $y_{p_0}$ is assumed to be unknown and estimated during the deconvolution. During the deconvolution, all the unknowns, i.e., $\varvec{\tau }$, ${\textbf {u}}$, and ${\textbf {q}}$ are identified by solving an optimization problem in a coordinate descent manner that utilizes physiological prior information and generalized-cross-validation. The details for the estimation are provided in^85^. For a long measurement, we split the data into multiple blocks of 200 s with a stride of 100 s to perform the deconvolution for each of these blocks. Later, all the results of ${\textbf {u}}$ are concatenated by discarding 50 s of the start and end part of the results to avoid inaccuracies in the boundaries of the deconvolution. Only for the first block and last block, we keep the first 50-s and last 50-s parts, respectively, as they cannot be replaced by results from the adjacent blocks.

Following^83,102^, we specify a first-order auto-regressive model for the hidden cognitive arousal state

22 $$\begin{aligned} x_{j+1}&= x_j + \varepsilon _j, \end{aligned}$$

where $x_j$ and $\varepsilon _j \sim {\mathscr {N}}(0,\sigma ^2_{\varepsilon })$ stand for internal cognitive arousal state and process noise at time *j*, respectively. Employing SCR events’ timing and their amplitudes as the observation, we intend to estimate the hidden arousal state using a marked point process Bayesian filter^83^. To this end, we consider the occurrence of a neural impulse $n_j$, as a Bernoulli-distributed random variable with probability mass function $a_j^{n_j}(1 - a_j)^{1 - n_j}$ where $a_j = P(n_j = 1)$. To relate relate $x_j$ to $a_j$, we use Sigmoid transfer function^102^

23 $$\begin{aligned} a_j&= \frac{1}{1 + e^{-(x_j + \beta)}}, \end{aligned}$$

where $\beta$ is a constant that can be calculated from $\beta \approx \log \Big (\frac{a_0}{1 - a_0}\Big)$ and $a_0$ represents the average probability of observing an impulse during the experiment. Similar to^83^, we define the continuous-valued amplitude $r_j$ of each neural impulse as

24 $$\begin{aligned} r_j&= \gamma _0 + \gamma _1x_j + v_j, \end{aligned}$$

where $r_j$ is the amplitude of the observed neural impulse due to ANS activation, $v_j\sim {\mathscr {N}}(0,\sigma ^2_{v})$ describes the sensor noise, $\gamma _0$ and $\gamma _1$ are the unknown parameters to be determined. Consequently, the joint density function for the observed neural stimuli is

25 $$\begin{aligned} p(n_j \cap r_j|x_j)={\left\{ \begin{array}{ll} 1-a_j \qquad \qquad \qquad &{}\text {if }\,\, n_j = 0\\ a_j\frac{1}{\sqrt{2\pi \sigma _v^2}}e^{\frac{-(r_j-\gamma _0-\gamma _1x_j)^2}{2\sigma _v^2}} &{}\text {if }\,\, n_j = 1 \end{array}\right. }. \end{aligned}$$

Applying the expectation-maximization framework, we estimate the unknown parameters $\theta _A = \{\sigma ^2_{\varepsilon },\gamma _0,\gamma _1,\sigma ^2_{v}\}$, and hidden state $x_j$, simultaneously. The E-step equations have been derived based on the observations $R^J = \{(n_1,r_1),...,(n_J,r_J)\}$ up to time *J*. At the E-step, the main objective is to estimate $x_j$ and its variance. The forward filter consists of the prediction and updates steps.

*Prediction:*

26 $$\begin{aligned} x_{j|j-1} &= x_{j-1|j-1}, \end{aligned}$$

27 $$\begin{aligned} \sigma ^2_{j|j-1} &= \sigma ^2_{j-1|j-1} + \sigma ^2_{\varepsilon }. \end{aligned}$$

*Update:*

If $$n_{j}=0$$

28 $$\begin{aligned} x_{j|j}&= x_{j|j-1} + \sigma ^2_{j|j-1}(n_{j}-a_{j|j}), \end{aligned}$$

29 $$\begin{aligned} \sigma ^2_{j|j}&= \Bigg [\frac{1}{\sigma ^2_{j|j-1}}+a_{j|j}(1-a_{j|j})\Bigg ]^{-1}. \end{aligned}$$

If $$n_j=1$$

30 $$\begin{aligned} C_j&= \frac{\sigma ^2_{j|j-1}}{\gamma _1^2\sigma ^2_{j|j-1}+\sigma _v^2}, \end{aligned}$$

31 $$\begin{aligned} x_{j|j} &= x_{j|j-1}+ C_j\bigg [\sigma _v^2(n_j-a_{j|j}) +\gamma _1(r_j-\gamma _0-\gamma _1x_{j|j-1})\bigg ], \end{aligned}$$

32 $$\begin{aligned} \sigma ^2_{j|j} &= \Bigg [\frac{1}{\sigma _{j|j-1}^2}+a_{j|j}(1-a_{j|j})+\frac{\gamma _{1}^2}{\sigma _v^2}\Bigg ]^{-1}. \end{aligned}$$

To derive $x_{j|j}$ appears on both sides of Eqs. (28) and (31), we use Newton-Raphson method. Next we follow a smoother approach to derive s smooth estimate

33 $$\begin{aligned} A_{j} &= \frac{\sigma ^2_{j|j}}{\sigma ^2_{j+1|j}}, \end{aligned}$$

34 $$\begin{aligned} x_{j|J} &= x_{j|j}+A_{j}(x_{j+1|J}-x_{j+1|j}), \end{aligned}$$

35 $$\begin{aligned} \sigma ^2_{j|J}&= \sigma ^2_{j|j}+A^2_{j}(\sigma ^2_{j+1|J}-\sigma ^2_{j+1|j}). \end{aligned}$$

At the M-step, we define $\tilde{J} = \{j|n_j = 1\}$ to indicate the locations of neural impulse occurrences. Similar to^83,102^, we compute the expected values of $x_{j}^2$ and $x_{j}x_{j-1}$ as

36 $$\begin{aligned} \mathbb {E}[x_{j}^2] = x_{j|J}^2 + \sigma ^2_{j|J} \text {\ \ \ \ and \ \ } \mathbb {E}[x_{j+1}x_{j}] = x_{j+1|J}x_{j|J} + A_{j}\sigma ^2_{j+1|J}. \end{aligned}$$

Thereafter, we derive the log-likelihood function $Q_1$ and, we estimate the unknown parameters such that they maximize it. The $Q_1$ function is

37 $$\begin{aligned} Q_1&= \sum _{j=1}^J \mathbb {E}[n_j(\beta +x_j)-\log (1+e^{\beta + x_j})]\nonumber \\&+ \frac{-\tilde{J}}{2}\log (2\pi \sigma ^2_v)-\sum _{j\in \tilde{J}}\frac{\mathbb {E}\bigg [(r_j-\gamma _0-\gamma _1x_j)^2\bigg ]}{2\sigma _v^2} + \frac{-J}{2}\log (2\pi \sigma ^2_{\varepsilon })-\sum _{j=1}^{J}\frac{\mathbb {E}\bigg [(x_j-x_{j-1})^2\bigg ]}{2\sigma _{\varepsilon }^2}. \end{aligned}$$

The algorithm iterates between the E-step and the M-step until convergence.

## Conclusion

In this research, with the ultimate goal of implementing wearable machine interface architectures in real-world settings, we examined the effects of auditory, gustatory, and olfactory actuation in regulating internal brain states. We designed and performed two sets of experiments to systematically evaluate the effects of safe actuation in modulating brain states. We employed wearable devices to collect human physiological data while asking them to perform memory-related cognitive tasks. Utilizing only wearable devices provides us with an excellent opportunity to further examine the idea of practical implementation of the proposed algorithms. In experiment 1, different types of music were played for participants while they perform *n*-back tasks. In experiment 2, we explored the effects of applying olfactory stimuli (i.e., smelling perfume) and drinking coffee on internal brain states. To validate our hypothesis about the effectiveness of this actuation (i.e., listening to music, smelling fragrances, and drinking coffee) in regulating brain states, we compared the results in all sessions (i.e., baseline sessions with no music and sessions with safe actuated conditions). To estimate cognitive performance, we collected and analyzed subjects’ correct/incorrect responses as well as their reaction times. To explore the effects of safe actuation in regulating physiological signals and cognitive arousal, we analyzed changes in electrodermal activity collected via wristband devices and EEG signals collected via muse headband. The experimental results verify our hypothesis about the efficiency of the proposed safe actuation in regulating internal brain states.

As a result of these human-subject experiments, we collected multiple physiological data (i.e., EDA, BVP, PPG, 3-axis accelerometer data, skin temperature, EEG) using only wearable technologies. An important future direction of this research could be on analyzing various physiological data and exploring the corresponding biomarkers. There is potential for further analysis of the published dataset, which could enhance our understanding of human cognitive science. While the overall positive impacts of the proposed safe actuation in elevating the average levels of cognitive performance state in all participants are reported, different types of physiological responses have been observed in the conducted experiments. Thus, the person-specific analysis would shed light on the physiological bases of the observed improvements in participants’ cognitive performance levels. Performing additional experiments on more subjects would lead to reaching a more diverse dataset. Given the variability in subject-specific reactions and potential latency in physiological responses to various forms of actuation, it may be beneficial to model actuation dynamics and incorporate them into the development of wearable machine interface (WMI) architectures. The proposed future directions of the present research will deepen the understanding of sensory stimulation’s impact on cognitive states and help develop new interventions to enhance cognitive performance in a closed-loop system. In such practical closed-loop WMI architectures, a wearable device collects physiological data from humans in the loop, a decoder estimates the internal cognitive brain state(s), and a controller incorporates the personalized dynamics of safe actuation and suggests the appropriate safe actuation. Consequently, it brings the hidden states to the desired range within a closed-loop framework. With ongoing advances in wearable technologies, the proposed research would open avenues of opportunities addressing mental health-related disorders within the remote monitoring properties. Humankind would derive a benefit from the proposed real-time monitoring and regulation toolsets by receiving personalized effective suggestions and medications with minimized side effects to enhance their overall quality of life.

## Supplementary Information

Supplementary Information.

## Data Availability

All data generated or analyzed during this study are included in this published article and its supplementary information files. The datasets used and/or analyzed during the current study are available from the corresponding author upon reasonable request.
