# Supplementary material for: Regulation of brain cognitive states through auditory, gustatory, and olfactory stimulation with wearable monitoring
Source: Sci Rep. 2023 Aug 8;13:12399. doi: 10.1038/s41598-023-37829-z (PMC10409795; doi:10.1038/s41598-023-37829-z)
Supplement: Supplementary file 1 — Supplementary Information. [file 41598_2023_37829_MOESM1_ESM.pdf]

Regulation of Brain Cognitive States through Auditory, Gustatory, and Olfactory Stimulation with Wearable Monitoring

Hamid Fekri Azgomi[1,2], Luciano R. F. Branco[1,3], Md. Rafiul Amin[1], Saman Khazaei[1,4], and Rose T. Faghih[1,4,*]

[1]Electrical and Computer Engineering Department, University of Houston, Houston, Texas, 77004, United States
[2]Department of Neurological Surgery, University of California San Francisco, San Francisco, California, 94143, United States
[3]Biomedical Engineering Department, University of Houston, Houston, Texas, 77004, United States
[4]Department of Biomedical Engineering, New York University, New York, New York, 10003, United States
[*]Corresponding Author: rfaghih@nyu.edu

Supplementary Materials

The participants' information is presented in Table S1. In summary, in Experiment 1, 17 subjects (11 males, 6 females) were involved in total. In Experiment 2, 13 subjects (10 males, 3 females) were involved in total. In analyzing participants' data, seven participants were excluded due to the E-Prime program crashes ($N = 1$) for collecting participants' behavioral responses or the quality of wearable Empatica wristband data due to the motion artifacts and/or the lack of proper contact between the wearable's electrodes and participants' skin surface ($N = 6$) in experiment 1. In experiment 2, the data of ($N = 3$) participants were excluded similar to experiment 1. In this study, we utilized 10 participants for each experiment and labeled the subjects accordingly. The selected subjects in experiment 1 and experiment 2 are labeled as subjects A1 - A10 and B1 - B10, respectively.

N/A term in Table S1 refers to the fact that the participant did not participate in the experiment. During the data collection, we employed the empatica wristbands on both hands. Next, we visually inspected the skin conductance signal and included the data from one hand for our cognitive arousal analysis. The information regarding the analyzed hand in each subject/experiment is presented in Table S1.

The raw physiological data collected from all the subjects in experiment 1 are presented in Figures S1- S10. The correct/incorrect response, reaction times, and the estimated performance state from all the subjects in experiment 1 are presented in Figures S11- S20. The estimated arousal states based on the variations observed in the skin conductance signal from all the subjects in experiment 1 are presented in Figures S21- S30. The raw physiological data collected from all the subjects in experiment 2 are presented in Figures S31- S40. The correct/incorrect response, reaction times, and the estimated performance state from all the subjects in experiment 2 are presented in Figures S41- S50. The estimated arousal states based on the variations observed in the skin conductance signal from all the subjects in experiment 2 are presented in Figures S51- S60. The relationship between the beta band power in EEG signals and the estimated cognitive performance state for subjects in experiments 1 and 2 are presented in Figures S61- S62. The relationship between the normalized levels of estimated cognitive arousal and estimated cognitive performance state for subjects in experiments 1 and 2 are presented in Figures S63- S64.

Table S1. Subjects information.

Participants ID	Gender	Age	Dominant hand	**Experiment 1**		**Experiment 2**	
				Subject ID	Analyzed hand	Subject ID	Analyzed hand
1	Male	29	Right	A1	Right	Excluded	Excluded
2	Female	21	Right	A2	Left	B1	Left
3	Male	27	Right	A3	Right	B2	Right
4	Female	28	Right	A4	Left	Excluded	Excluded
5	Male	31	Right	Excluded	Excluded	B3	Right
6	Male	31	Right	A5	Right	B4	Right
7	Male	28	Right	Excluded	Excluded	B5	Left
8	Male	27	Right	A6	Left	B6	Left
9	Female	18	Right	Excluded	Excluded	N/A	N/A
10	Male	22	Left	Excluded	Excluded	Excluded	Excluded
11	Male	27	Right	Excluded	Excluded	N/A	N/A
12	Male	32	Right	Excluded	Excluded	B7	Left
13	Female	34	Right	Excluded	Excluded	B8	Left
14	Female	34	Right	A7	Right	N/A	N/A
15	Male	27	Right	N/A	N/A	B9	Left
16	Male	32	Right	N/A	N/A	B10	Left
19	Male	32	Right	A8	Right	N/A	N/A
26	Female	35	Right	A9	Right	N/A	N/A
27	Male	28	Left	A10	Right	N/A	N/A

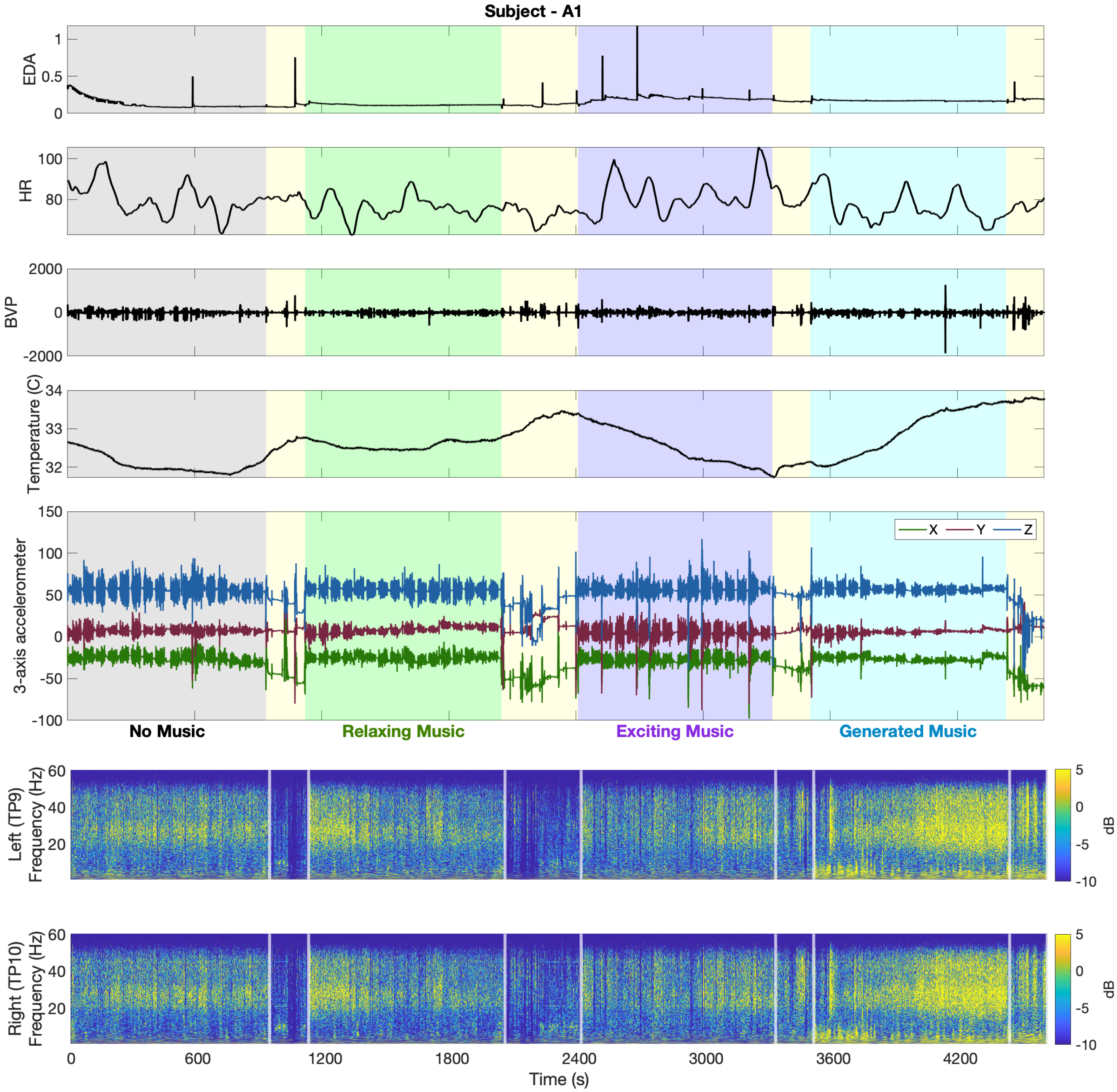

Figure S1. Raw Physiological Data Collected via Empatica E4 Wristband and Muse Headband (Subject - A1, Experiment 1). In the top panel, sub-panels in turn represent the EDA, HR, BVP, body temperature, and 3-axis accelerometer data. The grey, green, purple, and blue background colors in turn represent the results associated with no music, relaxing music, exciting music, and newly generated relaxing music sessions, respectively. Yellow backgrounds are associated with rest time periods. Sub-panels in the bottom panel depict spectrogram representations of EEG signals recorded from the left and right temporoparietal areas of the brain (i.e., TP9 and TP10). White vertical lines separate the memory tasks from rest times.

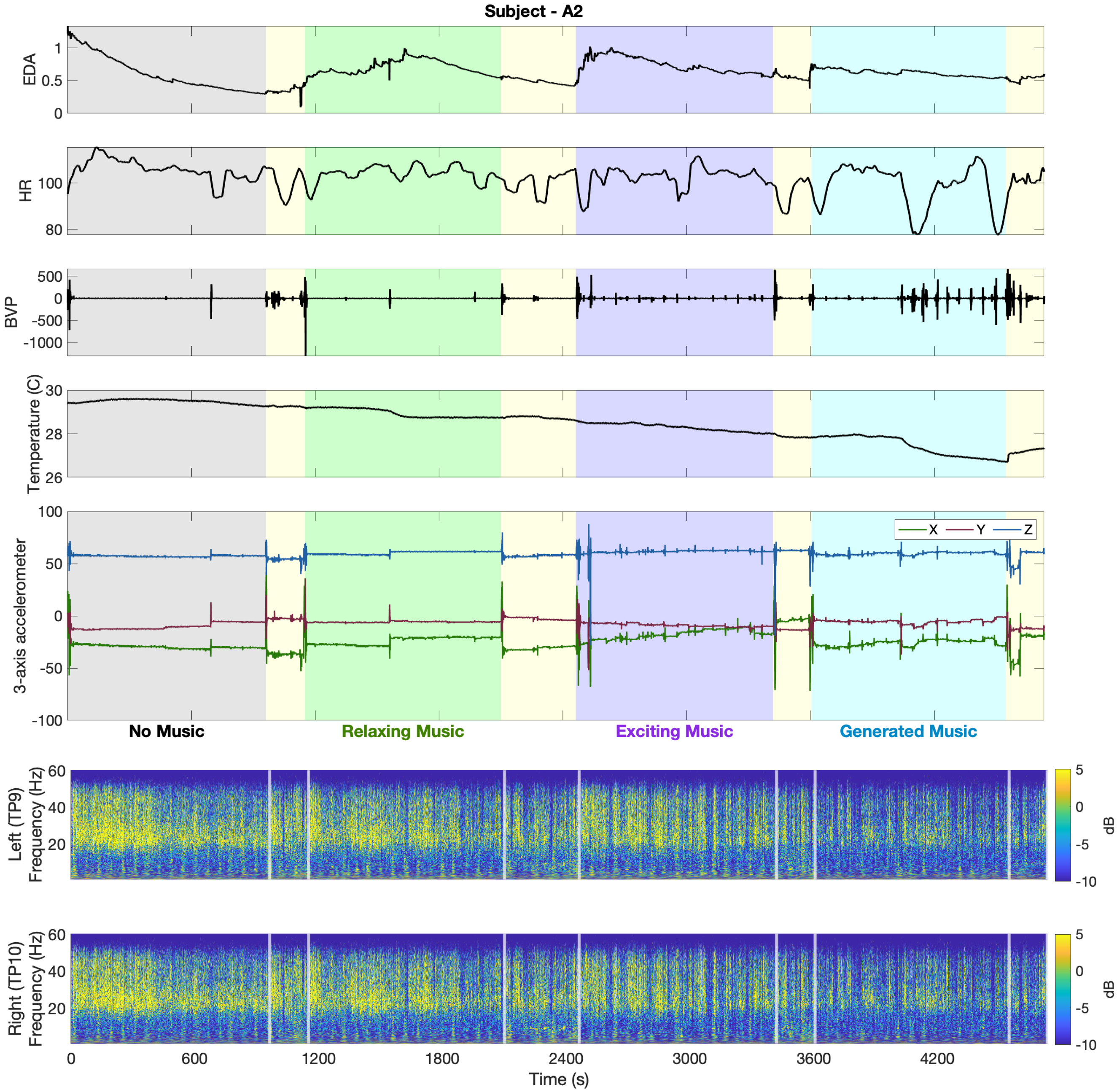

Figure S2. Raw Physiological Data Collected via Empatica E4 Wristband and Muse Headband (Subject - A2, Experiment 1). In the top panel, sub-panels in turn represent the EDA, HR, BVP, body temperature, and 3-axis accelerometer data. The grey, green, purple, and blue background colors in turn represent the results associated with no music, relaxing music, exciting music, and newly generated relaxing music sessions, respectively. Yellow backgrounds are associated with rest time periods. Sub-panels in the bottom panel depict spectrogram representations of EEG signals recorded from the left and right temporoparietal areas of the brain (i.e., TP9 and TP10). White vertical lines separate the memory tasks from rest times.

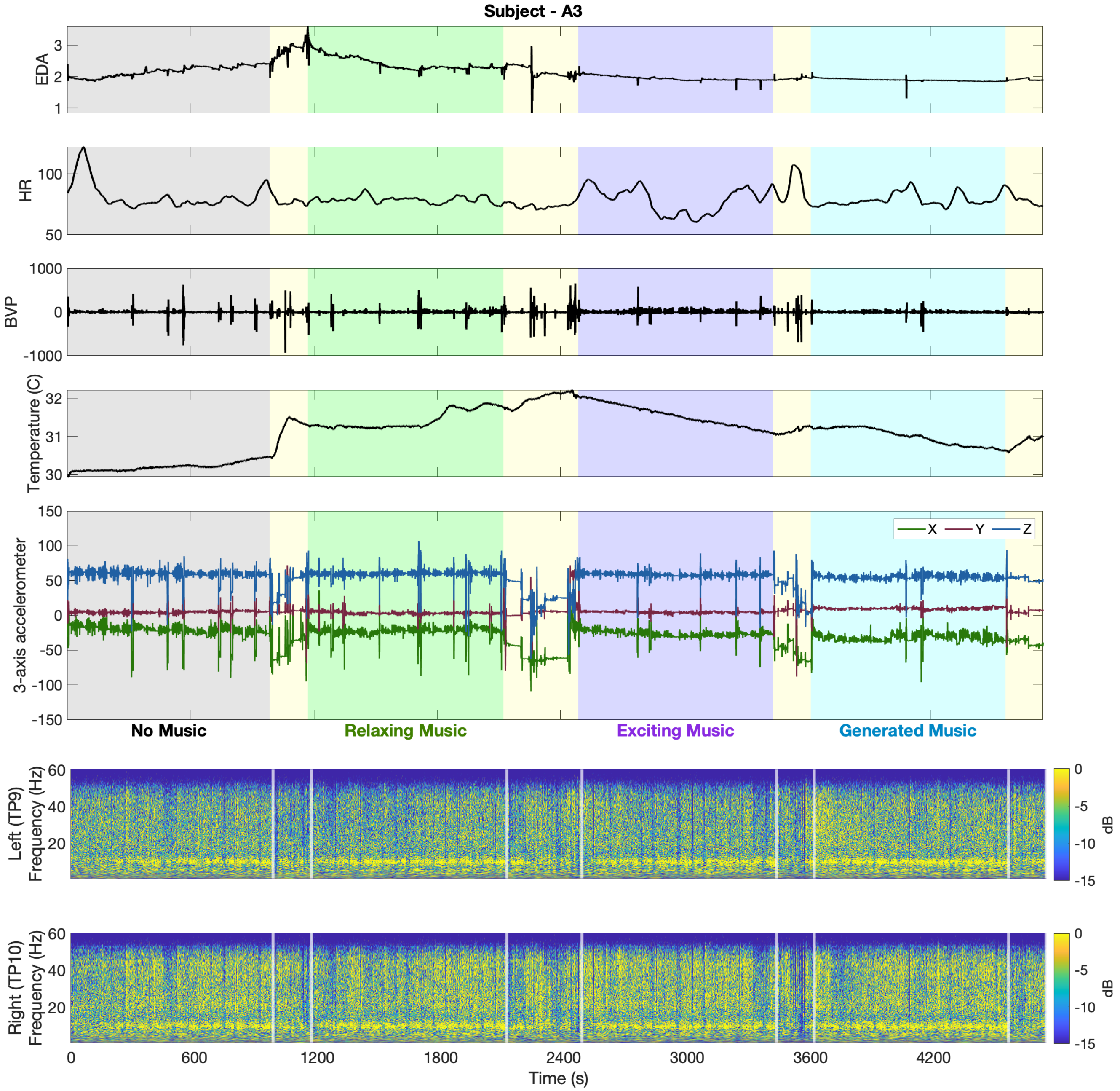

Figure S3. Raw Physiological Data Collected via Empatica E4 Wristband and Muse Headband (Subject - A3, Experiment 1). In the top panel, sub-panels in turn represent the EDA, HR, BVP, body temperature, and 3-axis accelerometer data. The grey, green, purple, and blue background colors in turn represent the results associated with no music, relaxing music, exciting music, and newly generated relaxing music sessions, respectively. Yellow backgrounds are associated with rest time periods. Sub-panels in the bottom panel depict spectrogram representations of EEG signals recorded from the left and right temporoparietal areas of the brain (i.e., TP9 and TP10). White vertical lines separate the memory tasks from rest times.

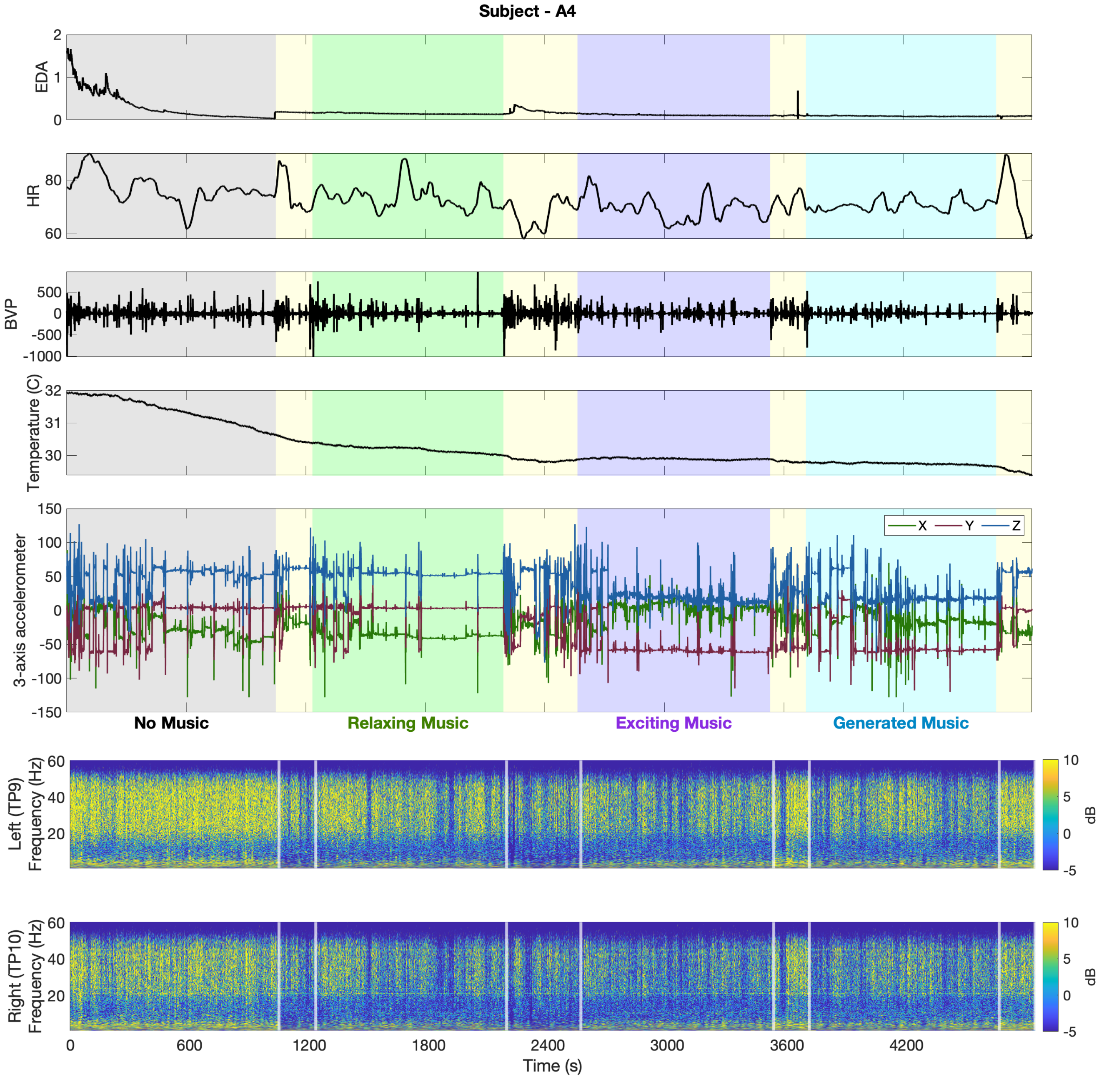

Figure S4. Raw Physiological Data Collected via Empatica E4 Wristband and Muse Headband (Subject - A4, Experiment 1). In the top panel, sub-panels in turn represent the EDA, HR, BVP, body temperature, and 3-axis accelerometer data. The grey, green, purple, and blue background colors in turn represent the results associated with no music, relaxing music, exciting music, and newly generated relaxing music sessions, respectively. Yellow backgrounds are associated with rest time periods. Sub-panels in the bottom panel depict spectrogram representations of EEG signals recorded from the left and right temporoparietal areas of the brain (i.e., TP9 and TP10). White vertical lines separate the memory tasks from rest times.

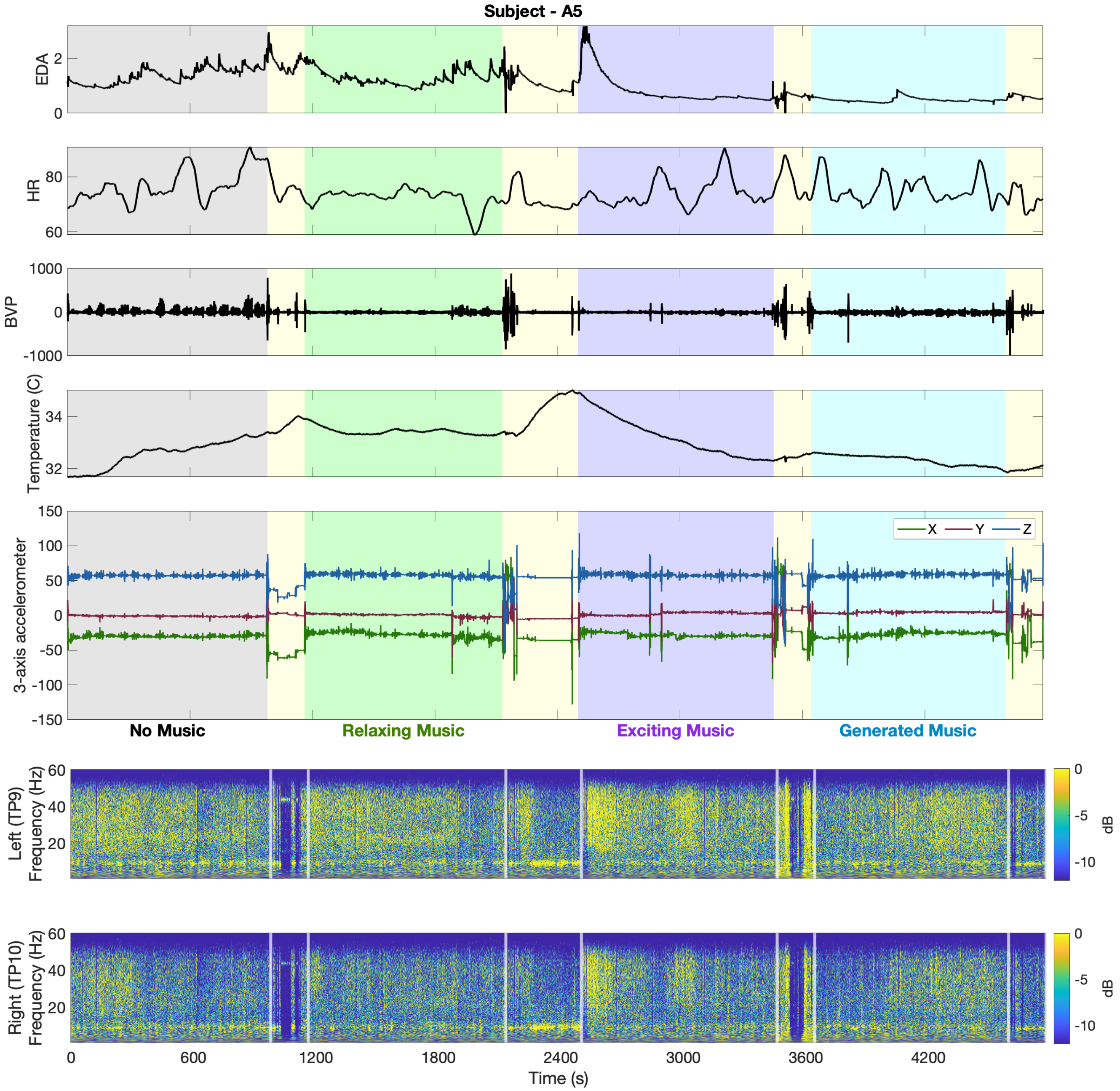

Figure S5. Raw Physiological Data Collected via Empatica E4 Wristband and Muse Headband (Subject - A5, Experiment 1). In the top panel, sub-panels in turn represent the EDA, HR, BVP, body temperature, and 3-axis accelerometer data. The grey, green, purple, and blue background colors in turn represent the results associated with no music, relaxing music, exciting music, and newly generated relaxing music sessions, respectively. Yellow backgrounds are associated with rest time periods. Sub-panels in the bottom panel depict spectrogram representations of EEG signals recorded from the left and right temporoparietal areas of the brain (i.e., TP9 and TP10). White vertical lines separate the memory tasks from rest times.

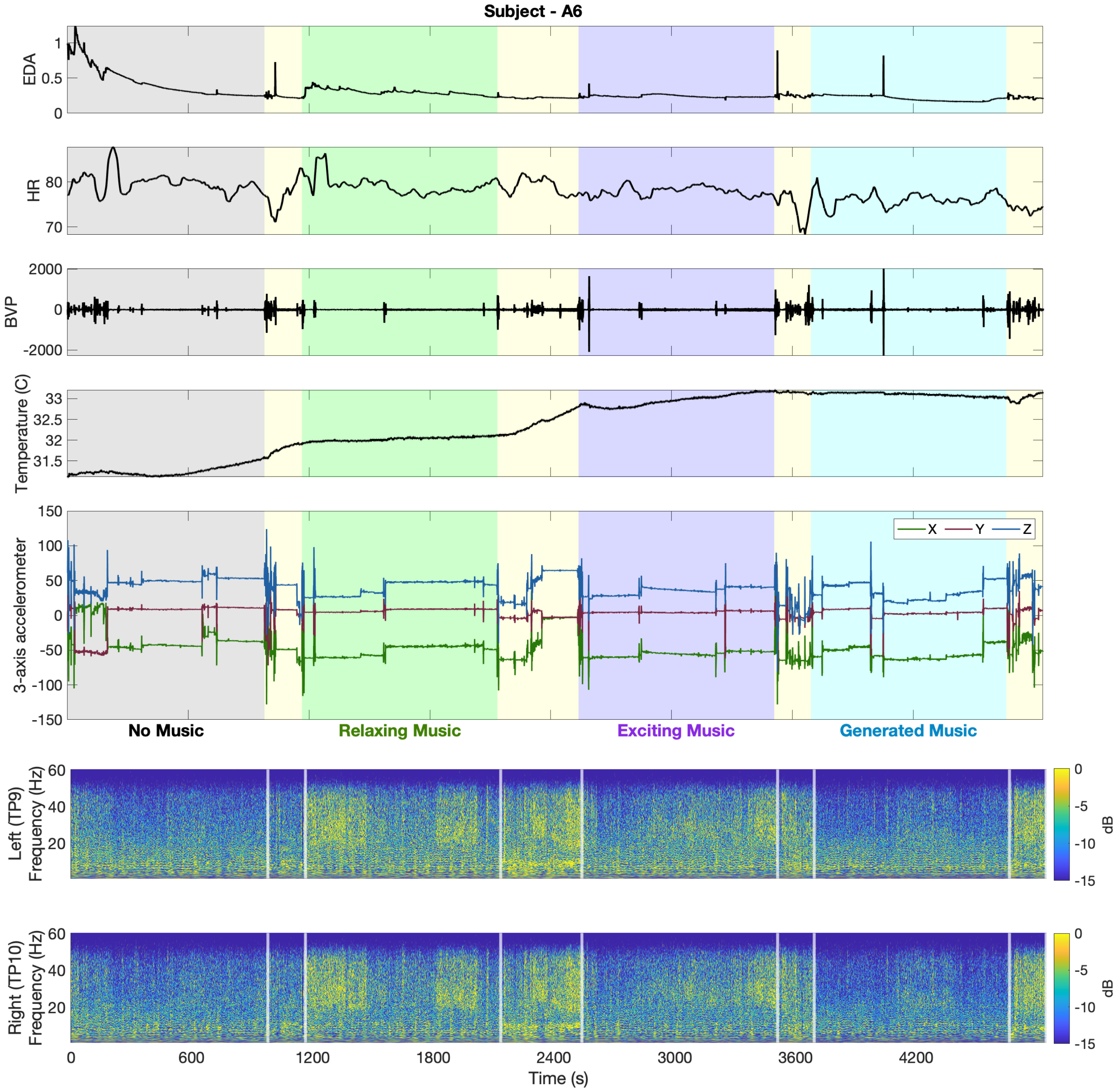

Figure S6. Raw Physiological Data Collected via Empatica E4 Wristband and Muse Headband (Subject - A6, Experiment 1). In the top panel, sub-panels in turn represent the EDA, HR, BVP, body temperature, and 3-axis accelerometer data. The grey, green, purple, and blue background colors in turn represent the results associated with no music, relaxing music, exciting music, and newly generated relaxing music sessions, respectively. Yellow backgrounds are associated with rest time periods. Sub-panels in the bottom panel depict spectrogram representations of EEG signals recorded from the left and right temporoparietal areas of the brain (i.e., TP9 and TP10). White vertical lines separate the memory tasks from rest times.

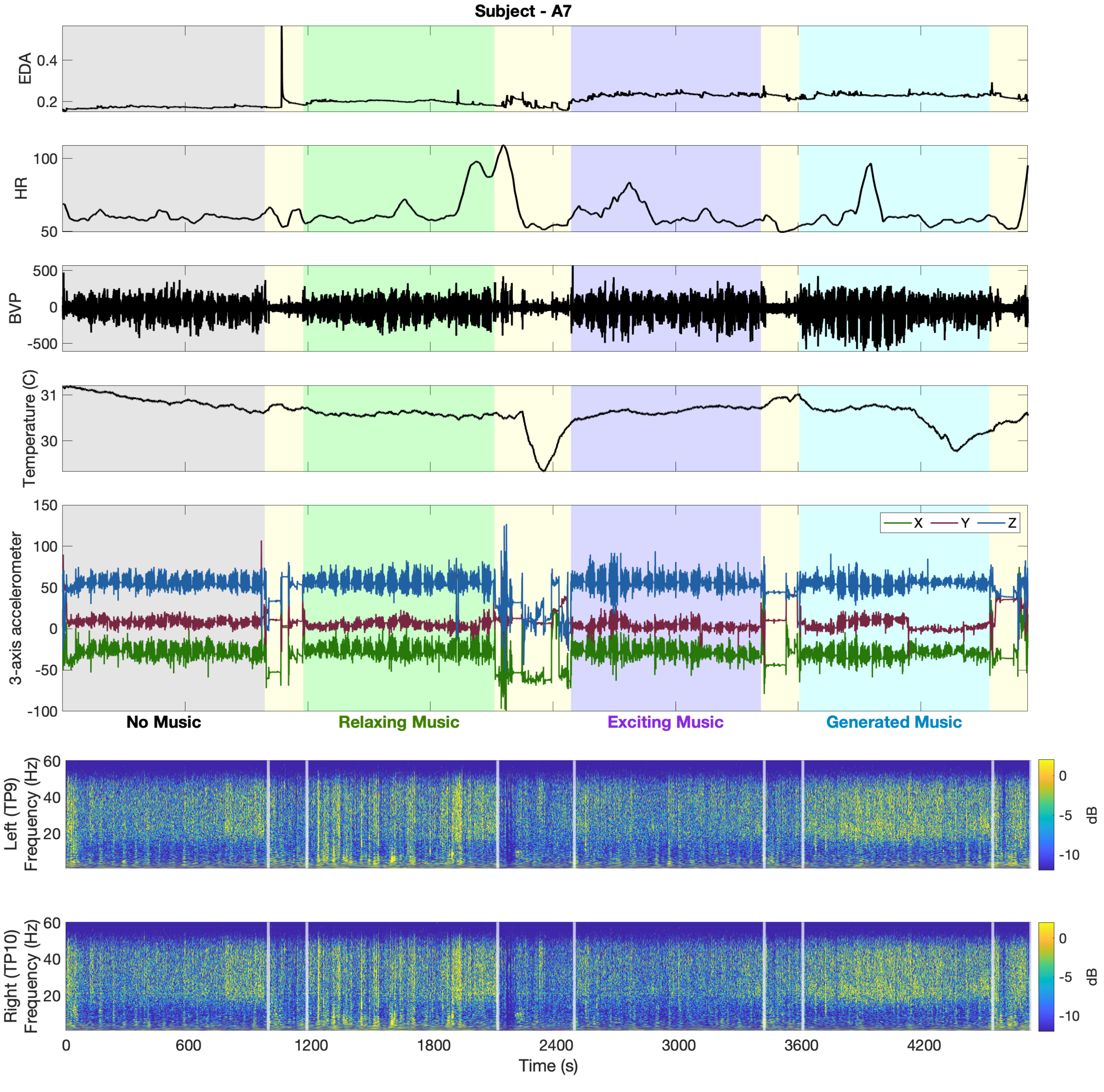

Figure S7. Raw Physiological Data Collected via Empatica E4 Wristband and Muse Headband (Subject - A7, Experiment 1). In the top panel, sub-panels in turn represent the EDA, HR, BVP, body temperature, and 3-axis accelerometer data. The grey, green, purple, and blue background colors in turn represent the results associated with no music, relaxing music, exciting music, and newly generated relaxing music sessions, respectively. Yellow backgrounds are associated with rest time periods. Sub-panels in the bottom panel depict spectrogram representations of EEG signals recorded from the left and right temporoparietal areas of the brain (i.e., TP9 and TP10). White vertical lines separate the memory tasks from rest times.

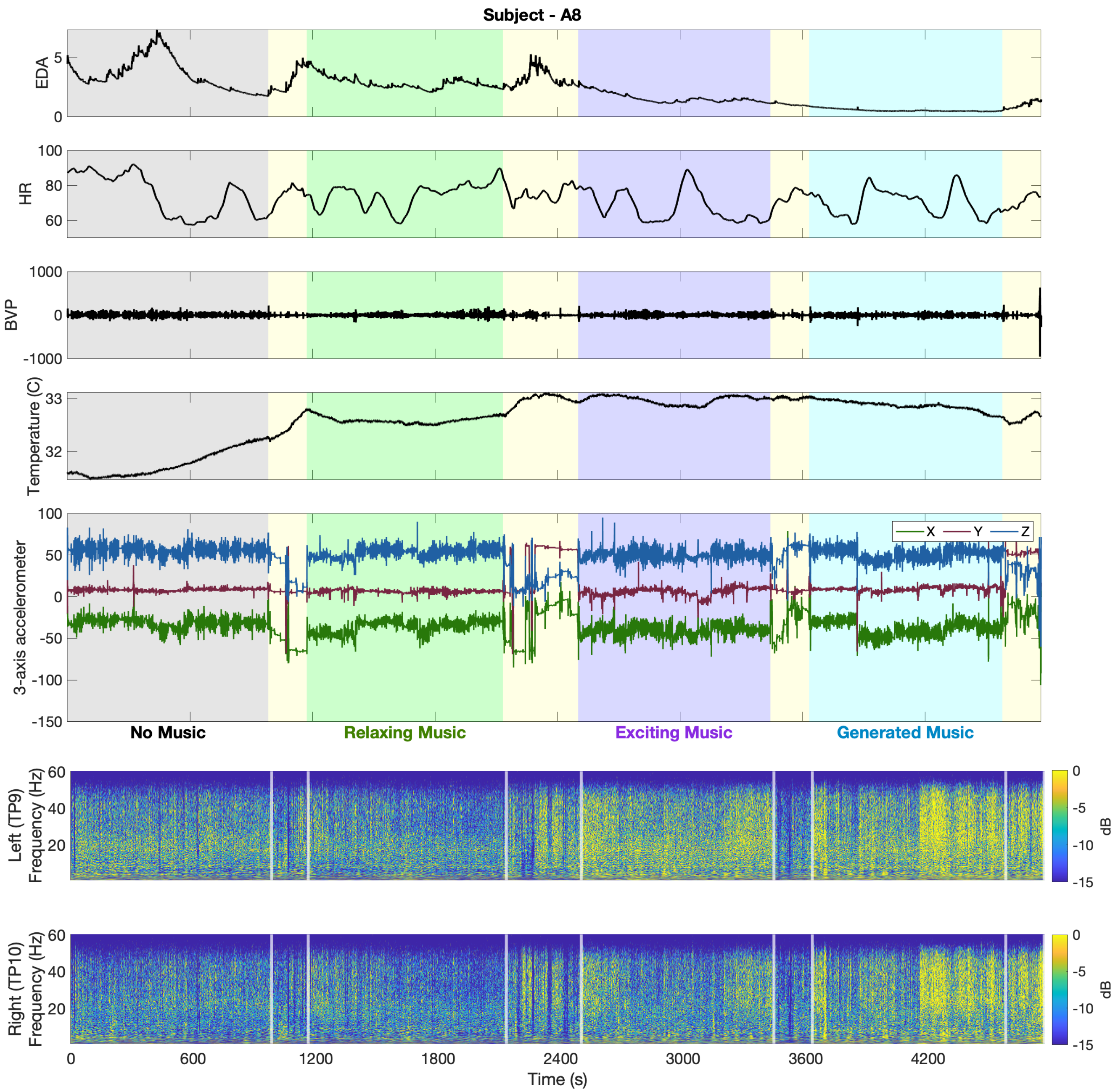

Figure S8. Raw Physiological Data Collected via Empatica E4 Wristband and Muse Headband (Subject - A8, Experiment 1). In the top panel, sub-panels in turn represent the EDA, HR, BVP, body temperature, and 3-axis accelerometer data. The grey, green, purple, and blue background colors in turn represent the results associated with no music, relaxing music, exciting music, and newly generated relaxing music sessions, respectively. Yellow backgrounds are associated with rest time periods. Sub-panels in the bottom panel depict spectrogram representations of EEG signals recorded from the left and right temporoparietal areas of the brain (i.e., TP9 and TP10). White vertical lines separate the memory tasks from rest times.

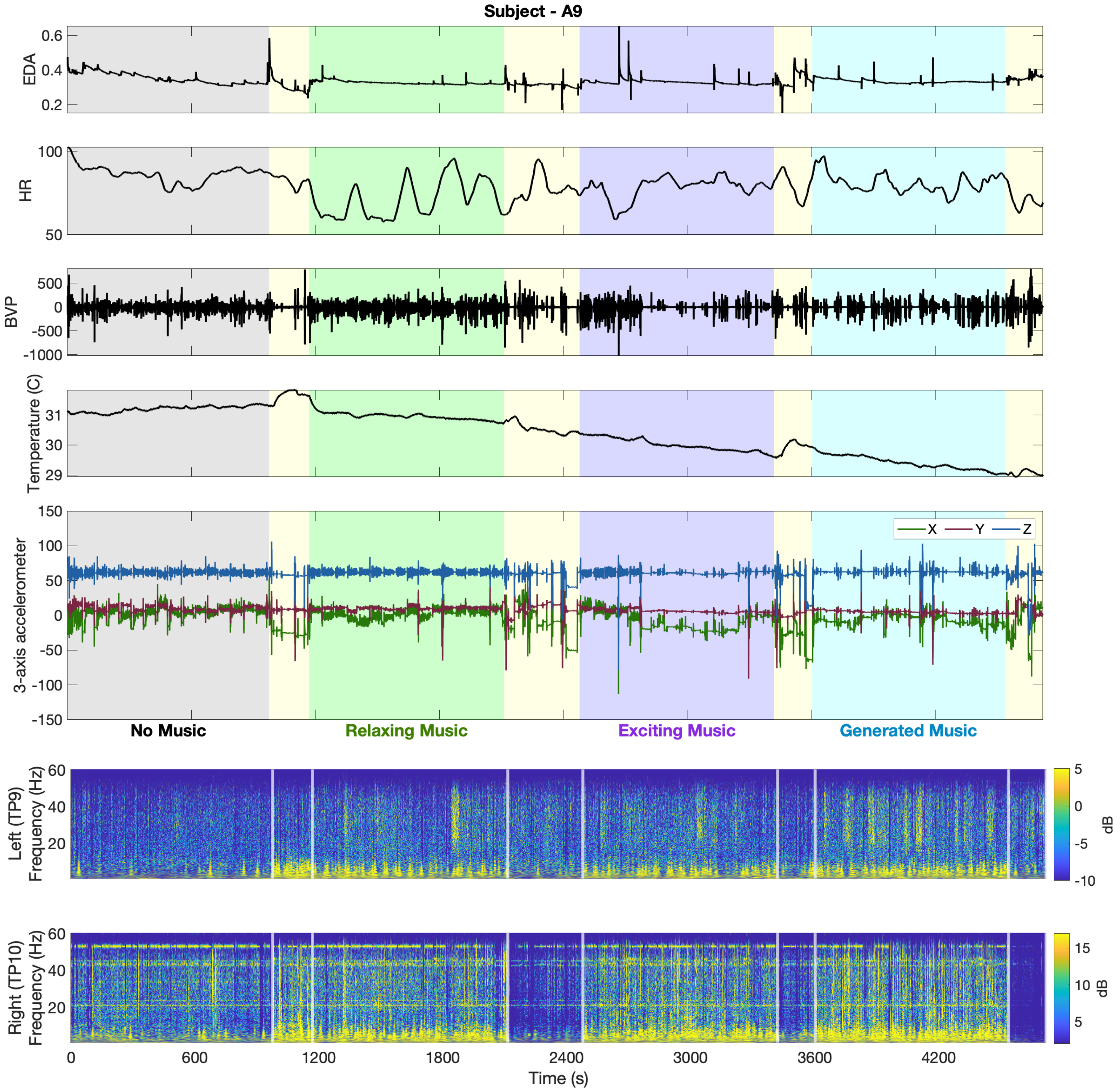

Figure S9. Raw Physiological Data Collected via Empatica E4 Wristband and Muse Headband (Subject - A9, Experiment 1). In the top panel, sub-panels in turn represent the EDA, HR, BVP, body temperature, and 3-axis accelerometer data. The grey, green, purple, and blue background colors in turn represent the results associated with no music, relaxing music, exciting music, and newly generated relaxing music sessions, respectively. Yellow backgrounds are associated with rest time periods. Sub-panels in the bottom panel depict spectrogram representations of EEG signals recorded from the left and right temporoparietal areas of the brain (i.e., TP9 and TP10). White vertical lines separate the memory tasks from rest times.

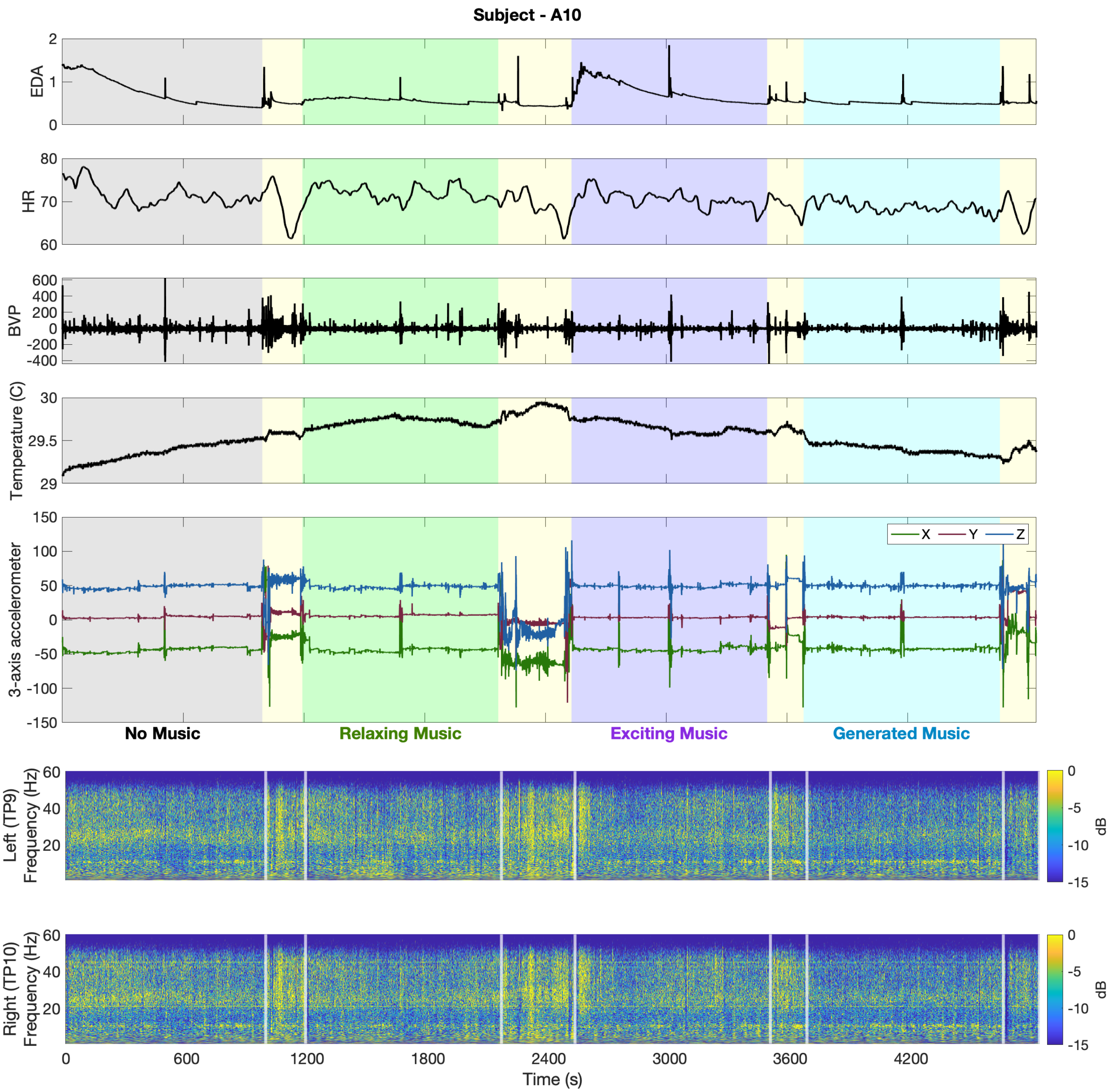

Figure S10. Raw Physiological Data Collected via Empatica E4 Wristband and Muse Headband (Subject - A10, Experiment 1). In the top panel, sub-panels in turn represent the EDA, HR, BVP, body temperature, and 3-axis accelerometer data. The grey, green, purple, and blue background colors in turn represent the results associated with no music, relaxing music, exciting music, and newly generated relaxing music sessions, respectively. Yellow backgrounds are associated with rest time periods. Sub-panels in the bottom panel depict spectrogram representations of EEG signals recorded from the left and right temporoparietal areas of the brain (i.e., TP9 and TP10). White vertical lines separate the memory tasks from rest times.

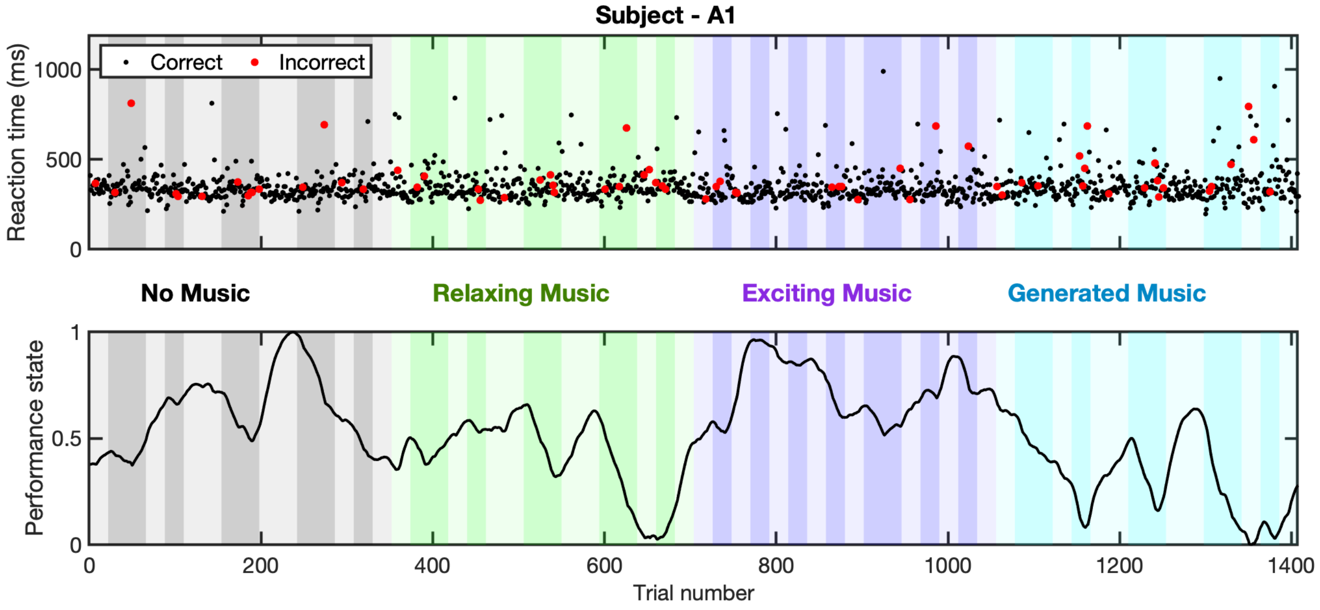

Figure S11. Cognitive Performance Results (Subject - A1, Experiment 1). The top panel shows the reaction time along with correct (black) and incorrect (red) responses. The bottom panel shows cognitive performance state estimates. The grey, green, purple, and blue background colors in turn represent the results associated with no music, relaxing music, exciting music, and newly generated relaxing music sessions, respectively. Bright and dark backgrounds present 1-back and 3-back tasks, respectively.

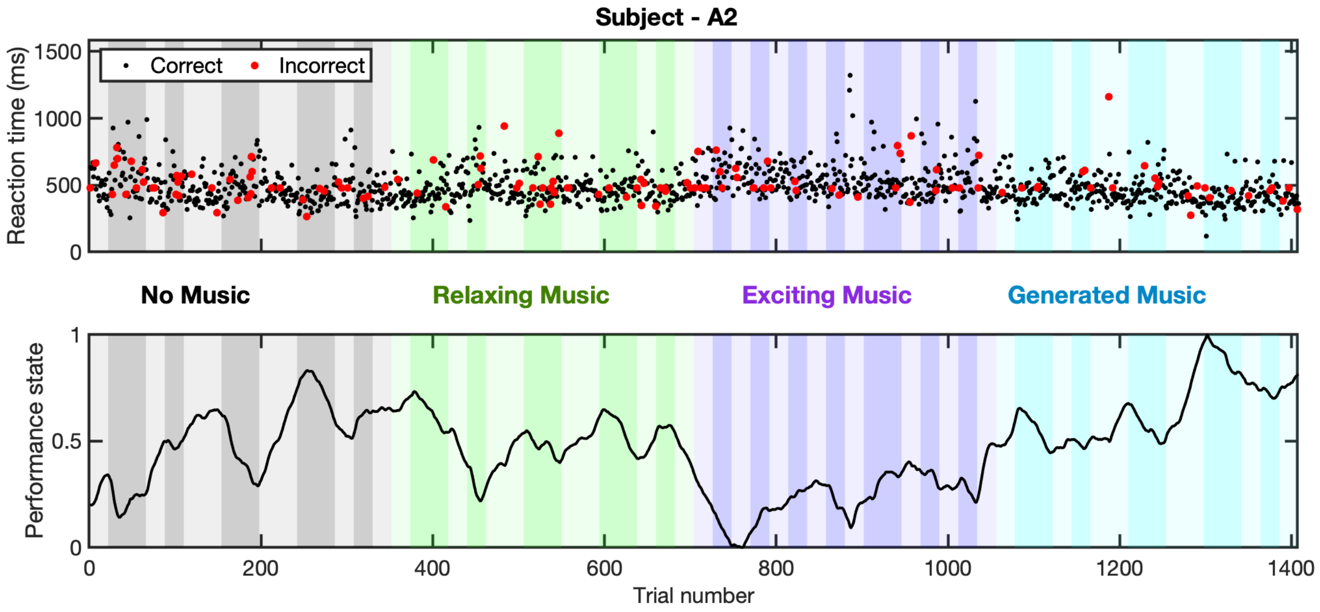

Figure S12. Cognitive Performance Results (Subject - A2, Experiment 1). The top panel shows the reaction time along with correct (black) and incorrect (red) responses. The bottom panel shows cognitive performance state estimates. The grey, green, purple, and blue background colors in turn represent the results associated with no music, relaxing music, exciting music, and newly generated relaxing music sessions, respectively. Bright and dark backgrounds present 1-back and 3-back tasks, respectively.

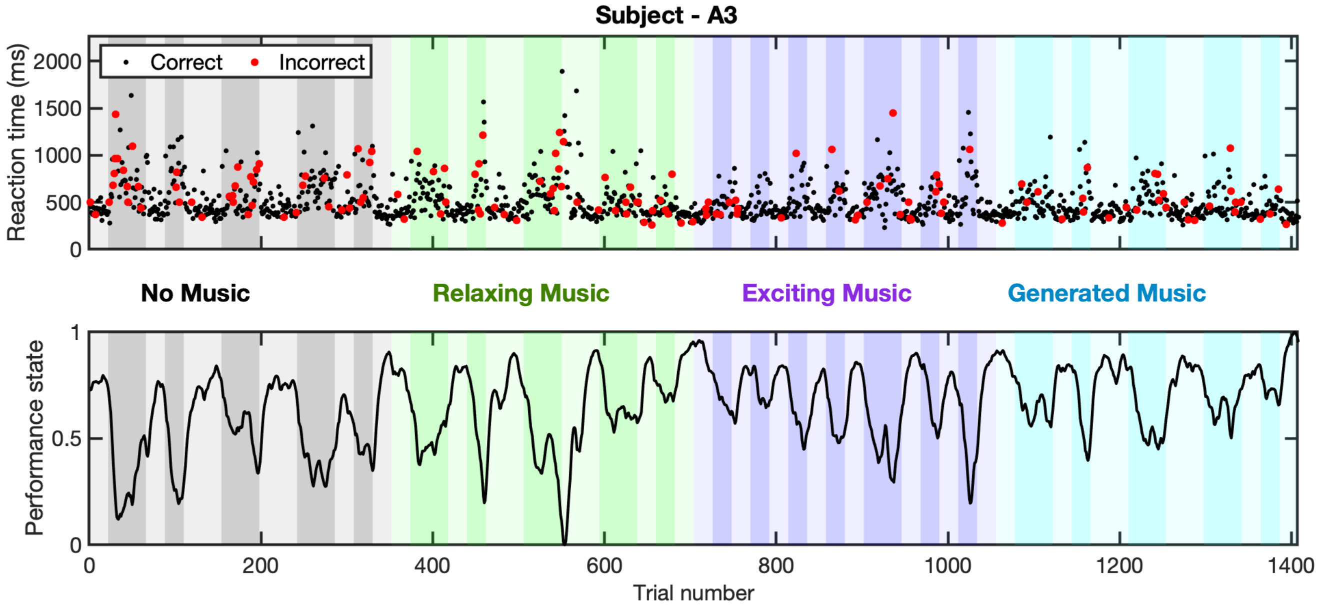

Figure S13. **Cognitive Performance Results (Subject - A3, Experiment 1).** The top panel shows the reaction time along with correct (black) and incorrect (red) responses. The bottom panel shows cognitive performance state estimates. The grey, green, purple, and blue background colors in turn represent the results associated with no music, relaxing music, exciting music, and newly generated relaxing music sessions, respectively. Bright and dark backgrounds present 1-back and 3-back tasks, respectively.

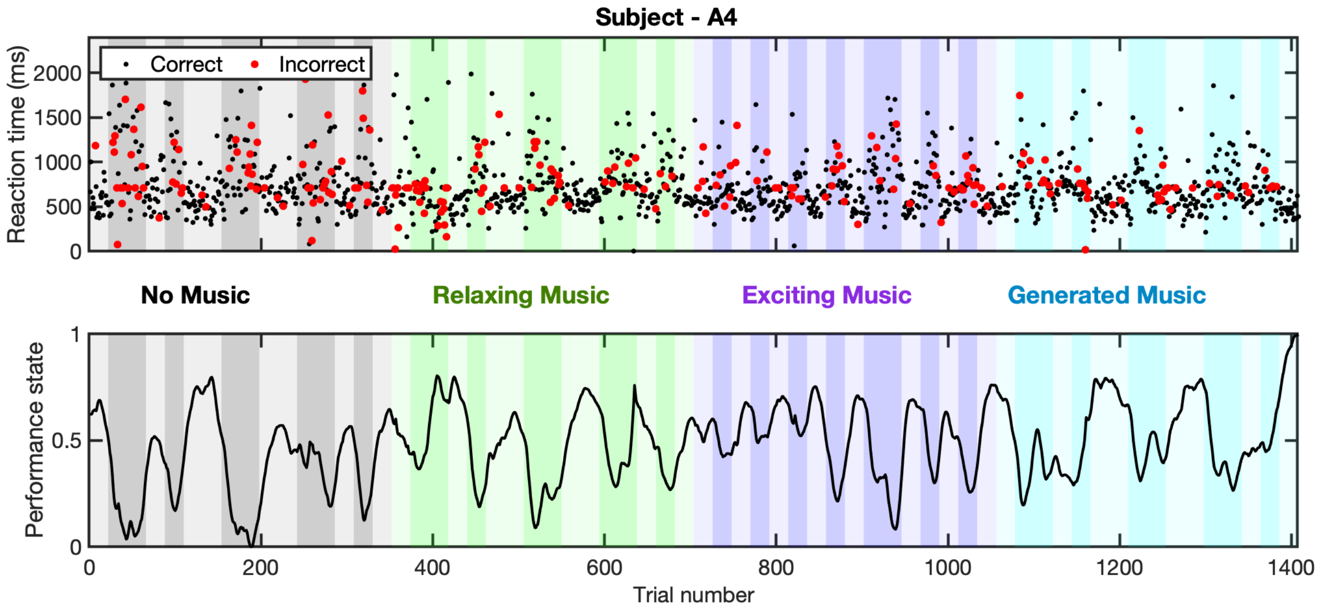

Figure S14. **Cognitive Performance Results (Subject - A4, Experiment 1).** The top panel shows the reaction time along with correct (black) and incorrect (red) responses. The bottom panel shows cognitive performance state estimates. The grey, green, purple, and blue background colors in turn represent the results associated with no music, relaxing music, exciting music, and newly generated relaxing music sessions, respectively. Bright and dark backgrounds present 1-back and 3-back tasks, respectively.

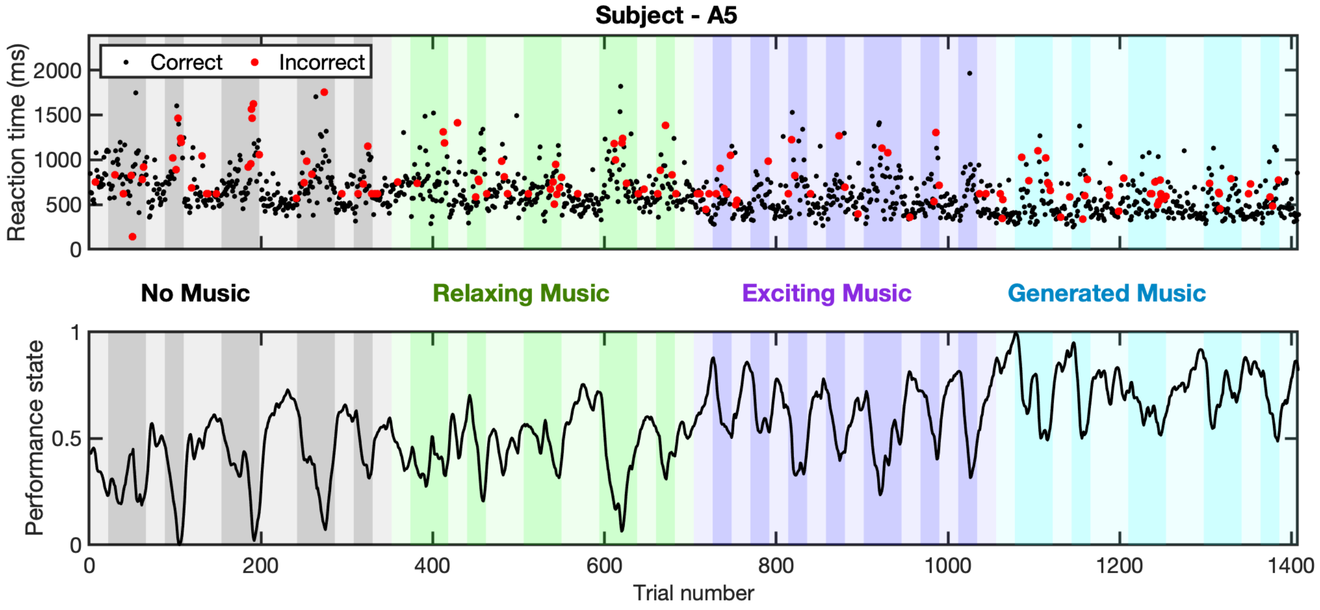

Figure S15. Cognitive Performance Results (Subject - A5, Experiment 1). The top panel shows the reaction time along with correct (black) and incorrect (red) responses. The bottom panel shows cognitive performance state estimates. The grey, green, purple, and blue background colors in turn represent the results associated with no music, relaxing music, exciting music, and newly generated relaxing music sessions, respectively. Bright and dark backgrounds present 1-back and 3-back tasks, respectively.

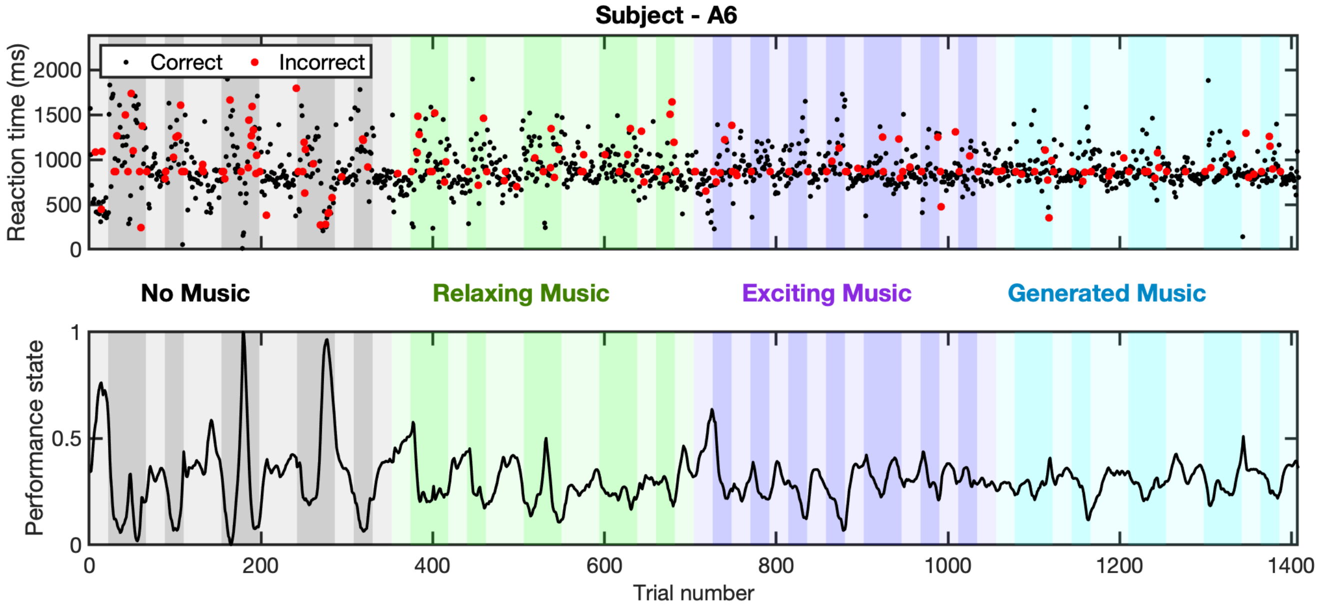

Figure S16. Cognitive Performance Results (Subject - A6, Experiment 1). The top panel shows the reaction time along with correct (black) and incorrect (red) responses. The bottom panel shows cognitive performance state estimates. The grey, green, purple, and blue background colors in turn represent the results associated with no music, relaxing music, exciting music, and newly generated relaxing music sessions, respectively. Bright and dark backgrounds present 1-back and 3-back tasks, respectively.

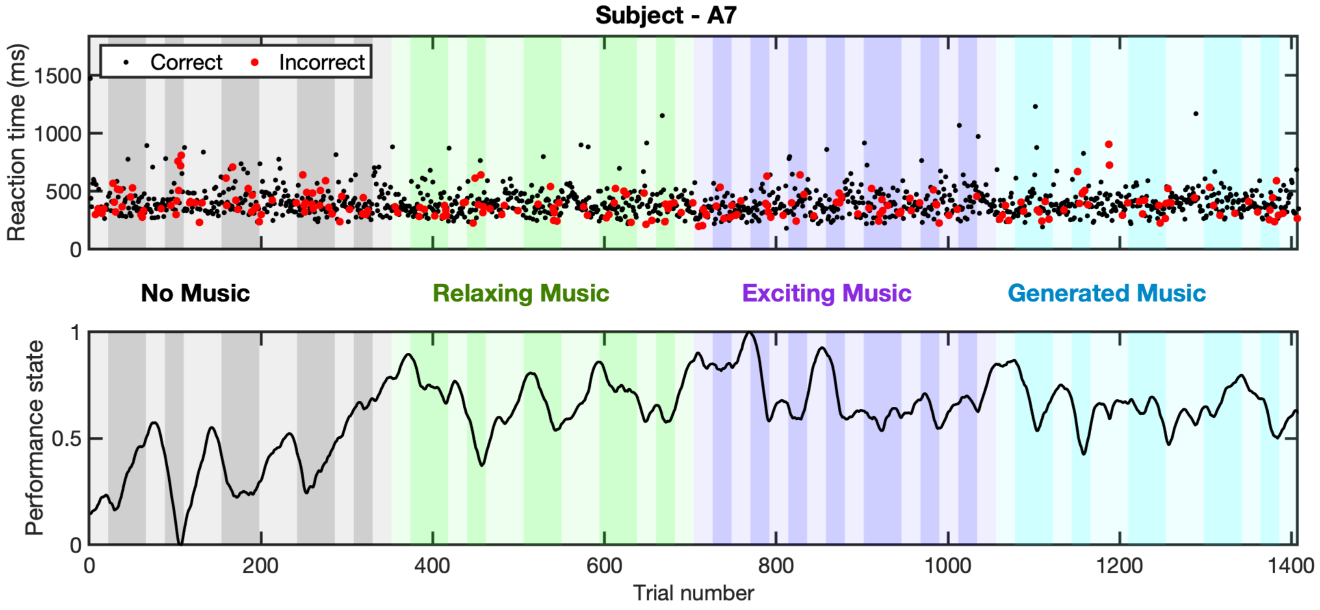

Figure S17. Cognitive Performance Results (Subject - A7, Experiment 1). The top panel shows the reaction time along with correct (black) and incorrect (red) responses. The bottom panel shows cognitive performance state estimates. The grey, green, purple, and blue background colors in turn represent the results associated with no music, relaxing music, exciting music, and newly generated relaxing music sessions, respectively. Bright and dark backgrounds present 1-back and 3-back tasks, respectively.

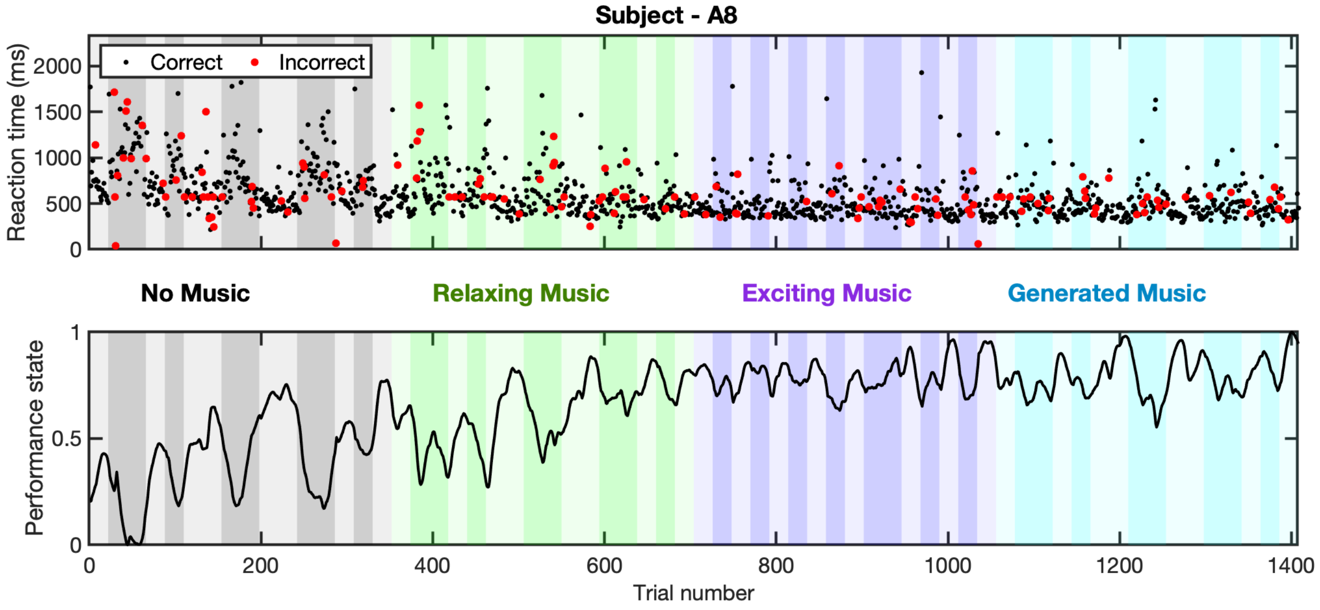

Figure S18. Cognitive Performance Results (Subject - A8, Experiment 1). The top panel shows the reaction time along with correct (black) and incorrect (red) responses. The bottom panel shows cognitive performance state estimates. The grey, green, purple, and blue background colors in turn represent the results associated with no music, relaxing music, exciting music, and newly generated relaxing music sessions, respectively. Bright and dark backgrounds present 1-back and 3-back tasks, respectively.

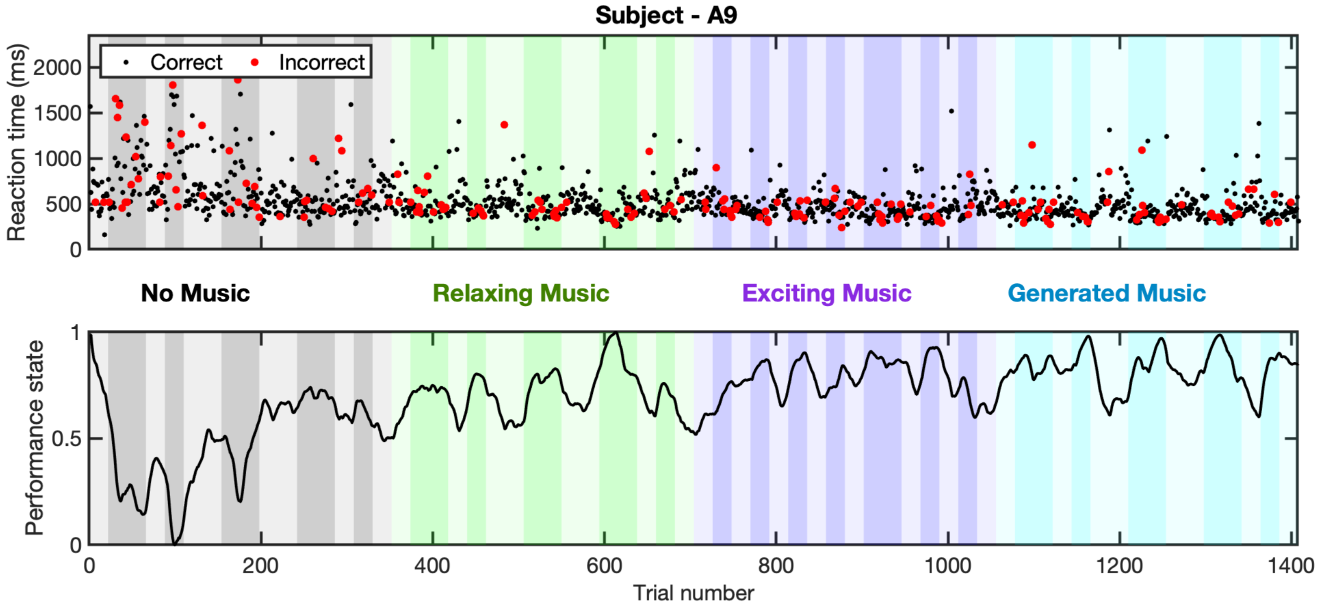

Figure S19. **Cognitive Performance Results (Subject - A9, Experiment 1).** The top panel shows the reaction time along with correct (black) and incorrect (red) responses. The bottom panel shows cognitive performance state estimates. The grey, green, purple, and blue background colors in turn represent the results associated with no music, relaxing music, exciting music, and newly generated relaxing music sessions, respectively. Bright and dark backgrounds present 1-back and 3-back tasks, respectively.

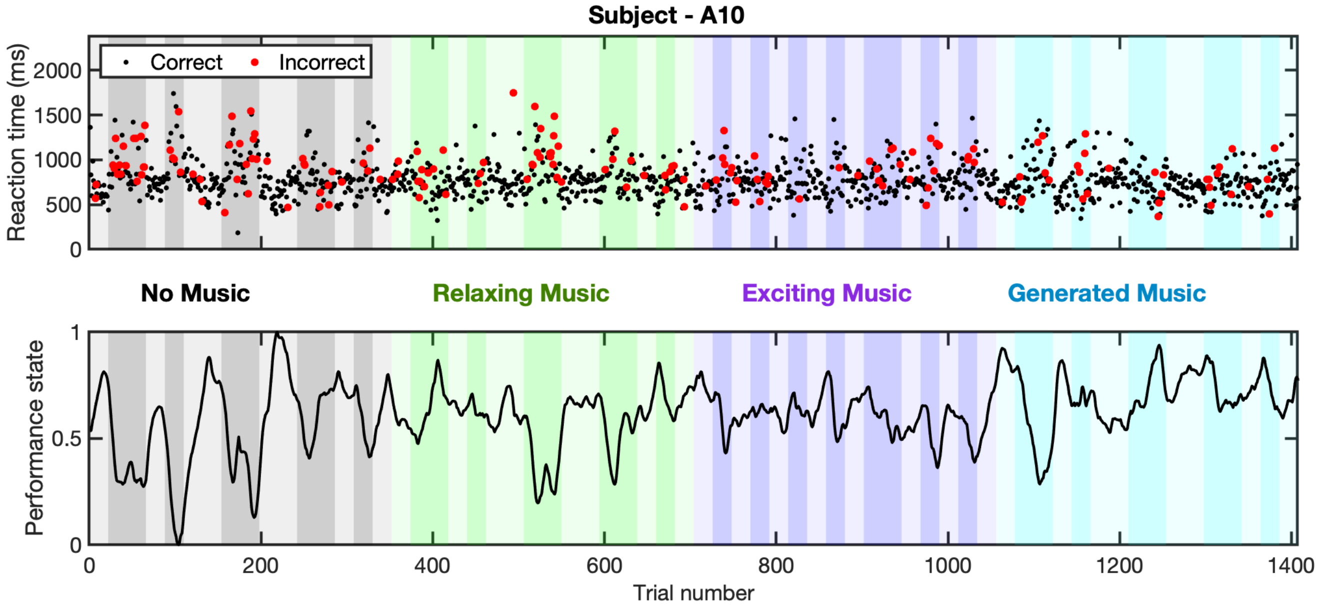

Figure S20. **Cognitive Performance Results (Subject - A10, Experiment 1).** The top panel shows the reaction time along with correct (black) and incorrect (red) responses. The bottom panel shows cognitive performance state estimates. The grey, green, purple, and blue background colors in turn represent the results associated with no music, relaxing music, exciting music, and newly generated relaxing music sessions, respectively. Bright and dark backgrounds present 1-back and 3-back tasks, respectively.

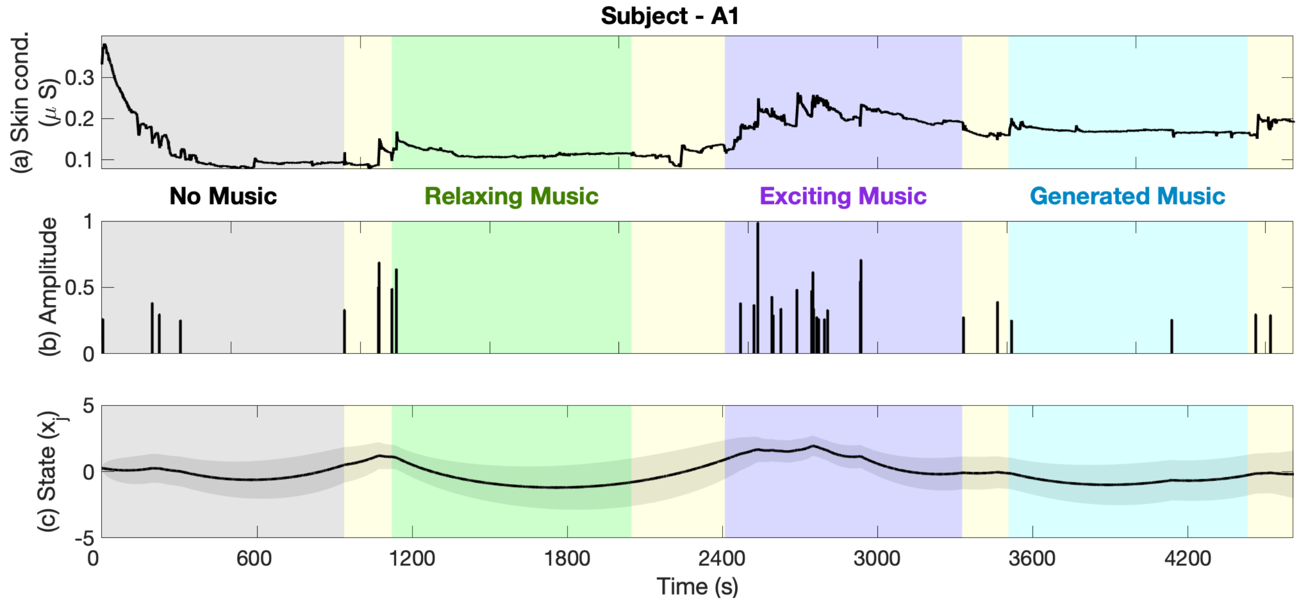

Figure S21. **Cognitive Arousal Results (Subject - A1, Experiment 1).** Panels from the top show in turn the skin conductance signal, underlying neural impulses, and estimated cognitive arousal state. The grey, green, purple, and blue background colors represent the results associated with no music, relaxing music, exciting music, and newly generated relaxing music sessions, respectively. Yellow backgrounds are associated with rest time periods.

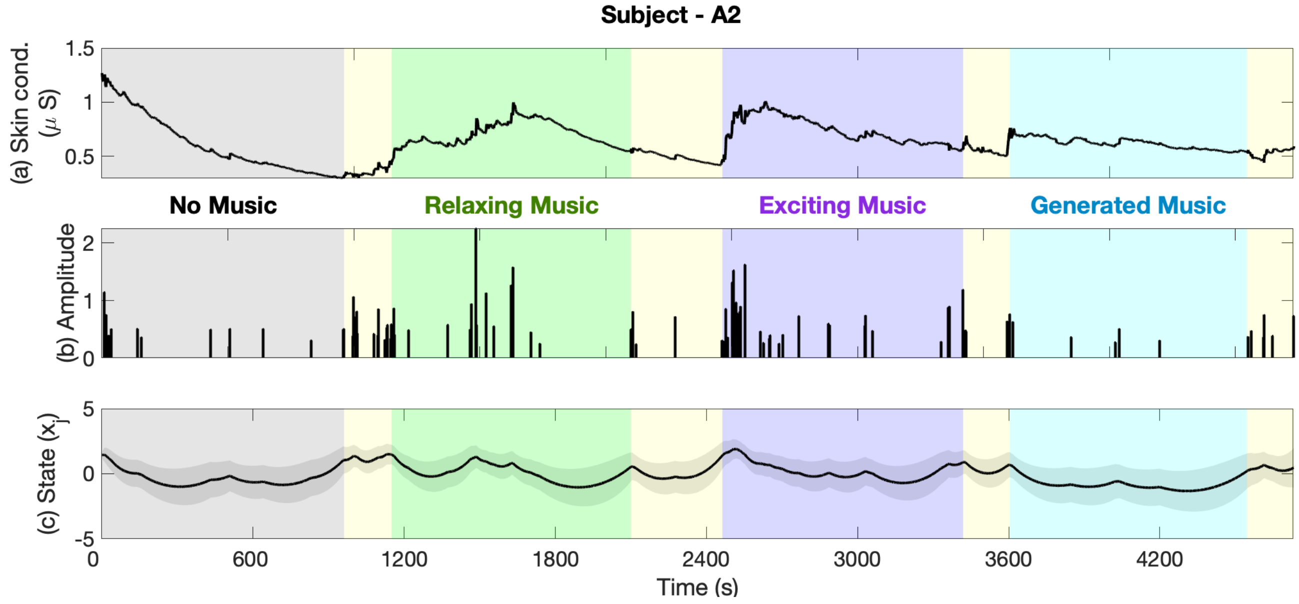

Figure S22. **Cognitive Arousal Results (Subject - A2, Experiment 1).** Panels from the top show in turn the skin conductance signal, underlying neural impulses, and estimated cognitive arousal state. The grey, green, purple, and blue background colors represent the results associated with no music, relaxing music, exciting music, and newly generated relaxing music sessions, respectively. Yellow backgrounds are associated with rest time periods.

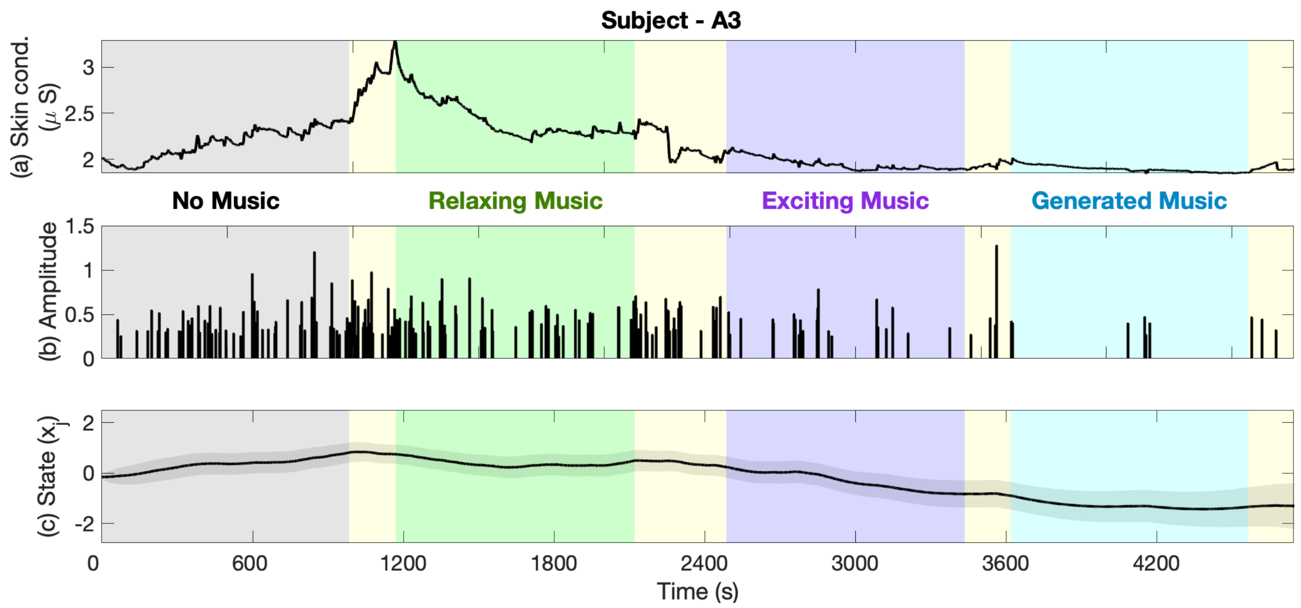

Figure S23. **Cognitive Arousal Results (Subject - A3, Experiment 1).** Panels from the top show in turn the skin conductance signal, underlying neural impulses, and estimated cognitive arousal state. The grey, green, purple, and blue background colors represent the results associated with no music, relaxing music, exciting music, and newly generated relaxing music sessions, respectively. Yellow backgrounds are associated with rest time periods.

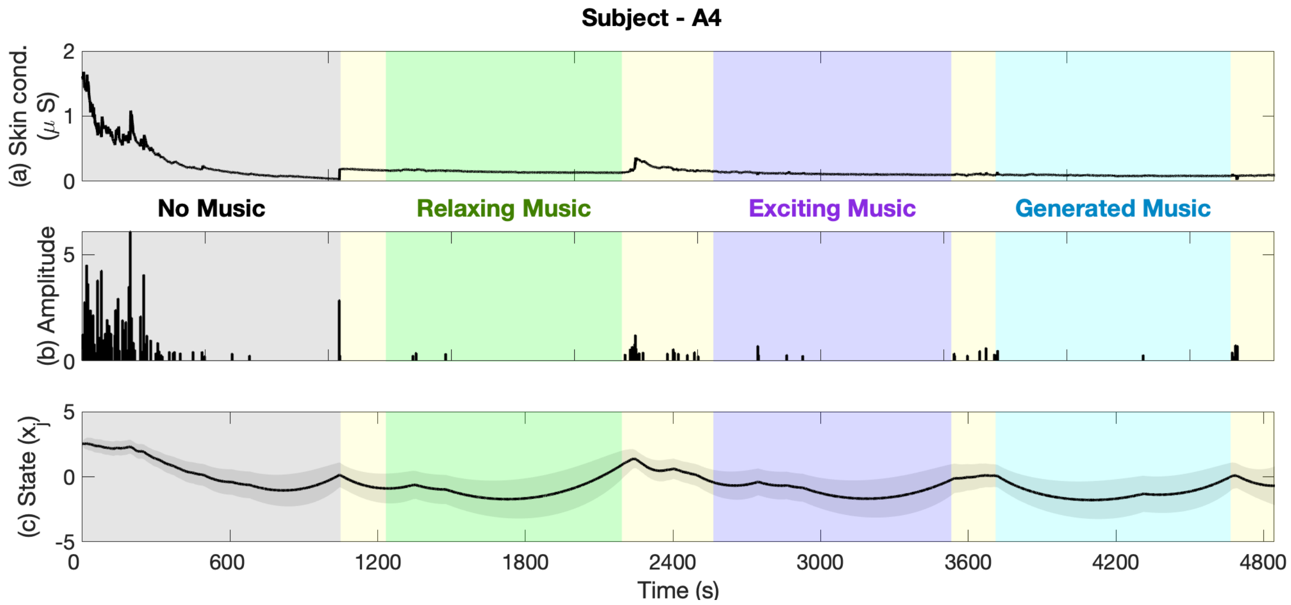

Figure S24. **Cognitive Arousal Results (Subject - A4, Experiment 1).** Panels from the top show in turn the skin conductance signal, underlying neural impulses, and estimated cognitive arousal state. The grey, green, purple, and blue background colors represent the results associated with no music, relaxing music, exciting music, and newly generated relaxing music sessions, respectively. Yellow backgrounds are associated with rest time periods.

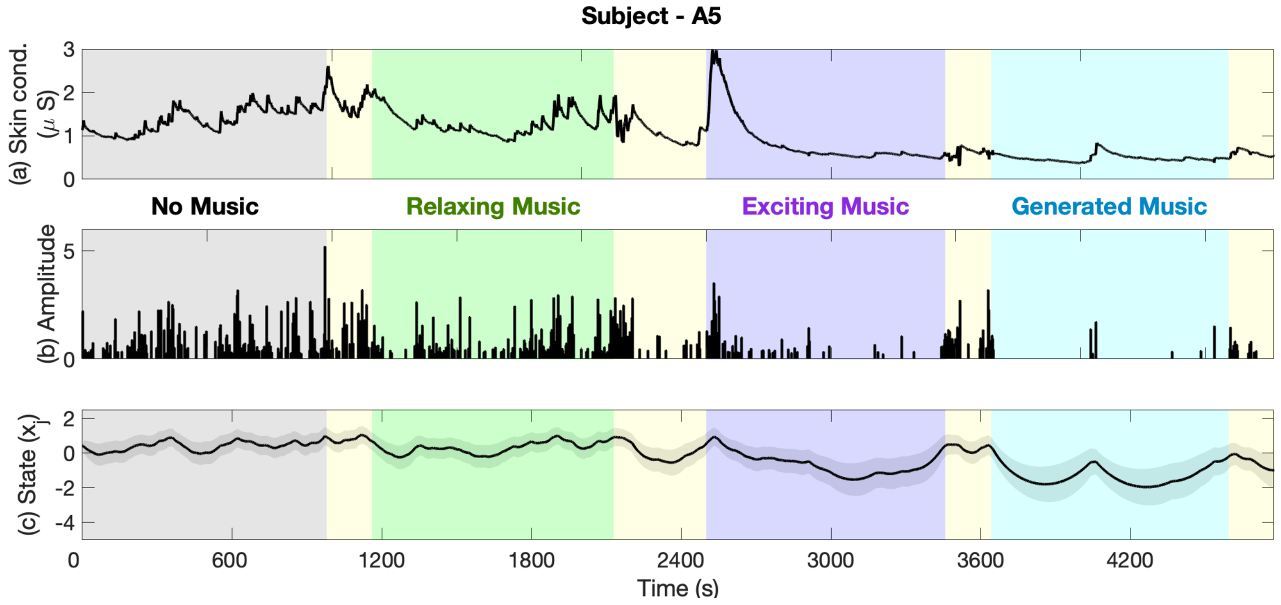

Figure S25. **Cognitive Arousal Results (Subject - A5, Experiment 1).** Panels from the top show in turn the skin conductance signal, underlying neural impulses, and estimated cognitive arousal state. The grey, green, purple, and blue background colors represent the results associated with no music, relaxing music, exciting music, and newly generated relaxing music sessions, respectively. Yellow backgrounds are associated with rest time periods.

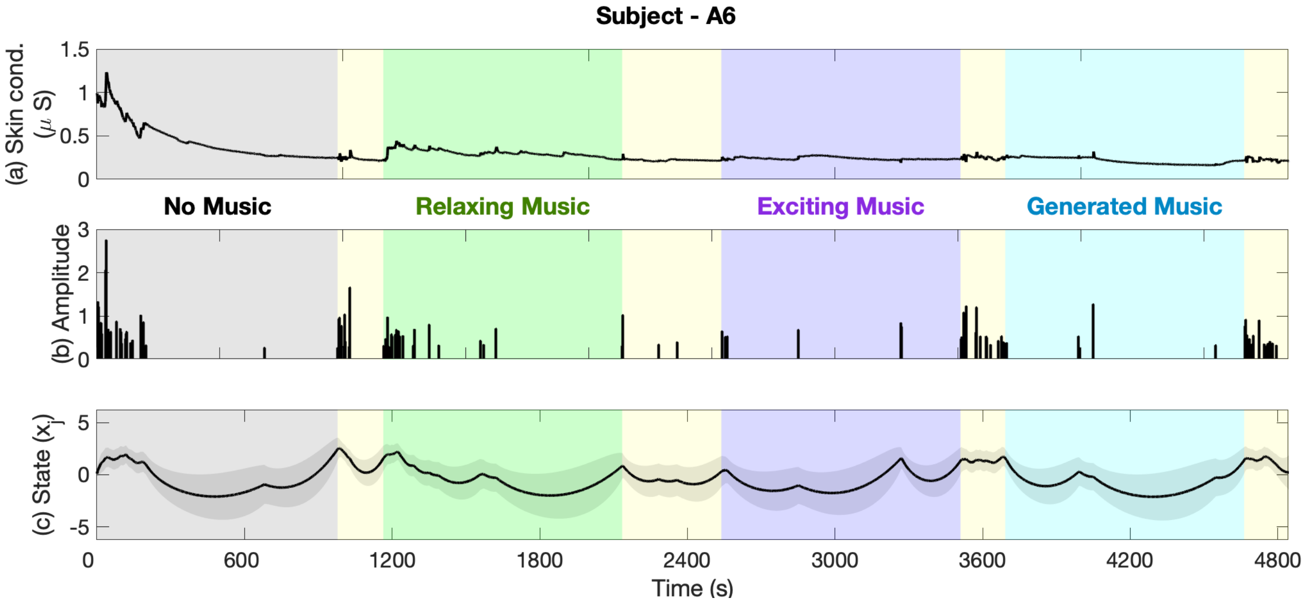

Figure S26. **Cognitive Arousal Results (Subject - A6, Experiment 1).** Panels from the top show in turn the skin conductance signal, underlying neural impulses, and estimated cognitive arousal state. The grey, green, purple, and blue background colors represent the results associated with no music, relaxing music, exciting music, and newly generated relaxing music sessions, respectively. Yellow backgrounds are associated with rest time periods.

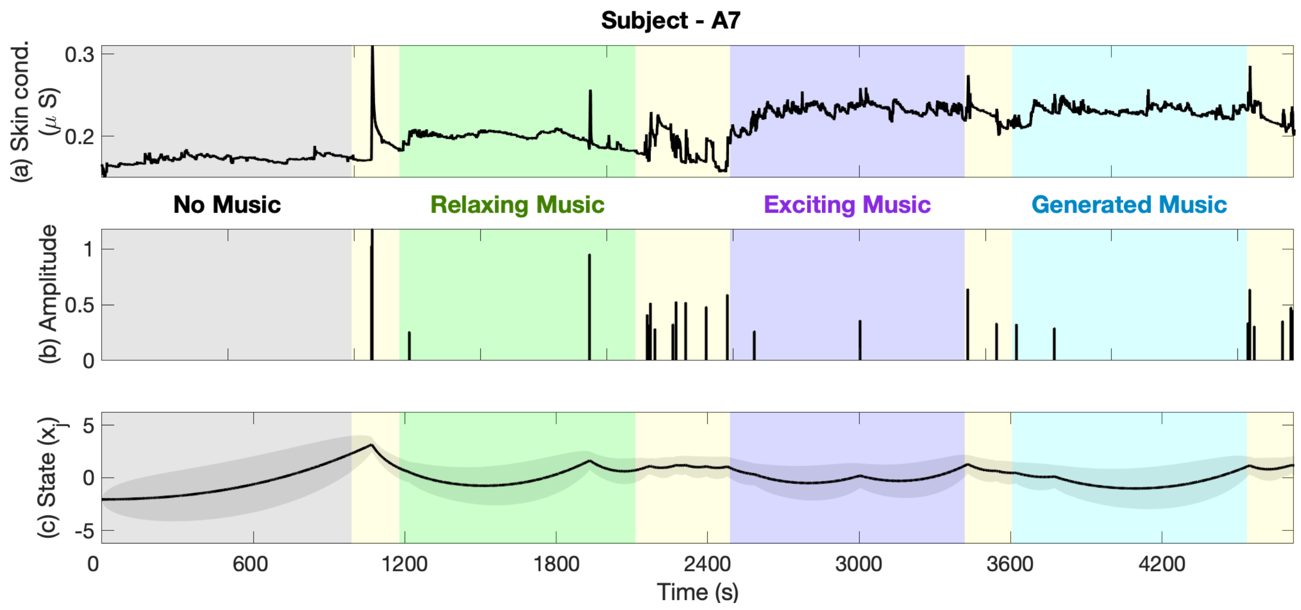

Figure S27. Cognitive Arousal Results (Subject - A7, Experiment 1). Panels from the top show in turn the skin conductance signal, underlying neural impulses, and estimated cognitive arousal state. The grey, green, purple, and blue background colors represent the results associated with no music, relaxing music, exciting music, and newly generated relaxing music sessions, respectively. Yellow backgrounds are associated with rest time periods.

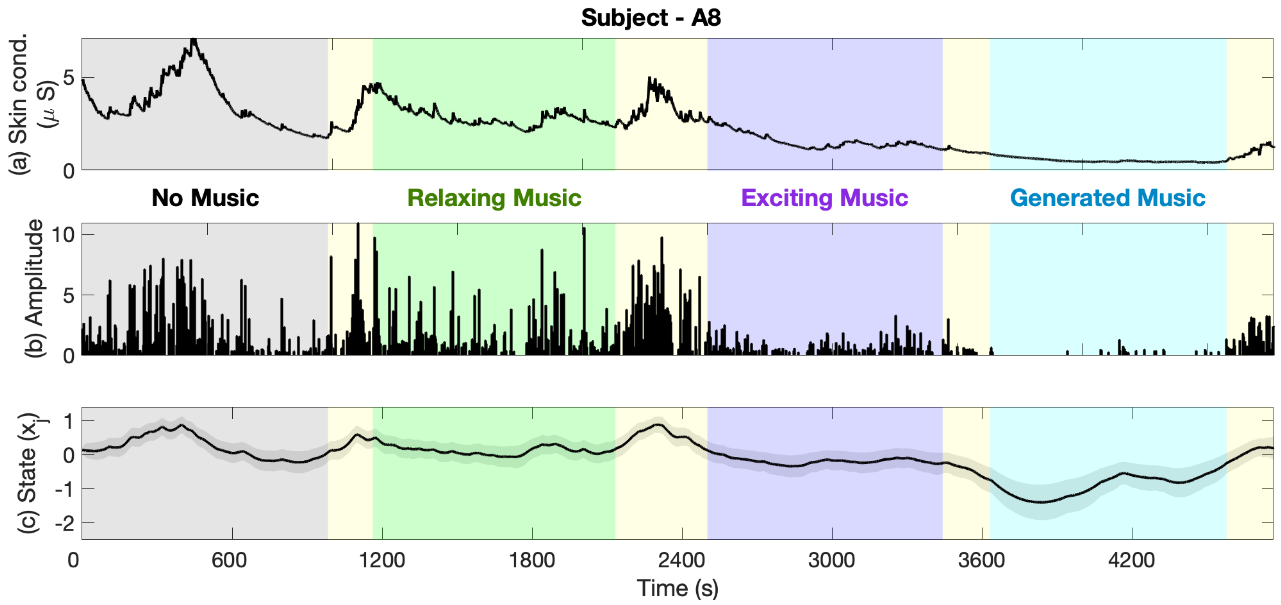

Figure S28. Cognitive Arousal Results (Subject - A8, Experiment 1). Panels from the top show in turn the skin conductance signal, underlying neural impulses, and estimated cognitive arousal state. The grey, green, purple, and blue background colors represent the results associated with no music, relaxing music, exciting music, and newly generated relaxing music sessions, respectively. Yellow backgrounds are associated with rest time periods.

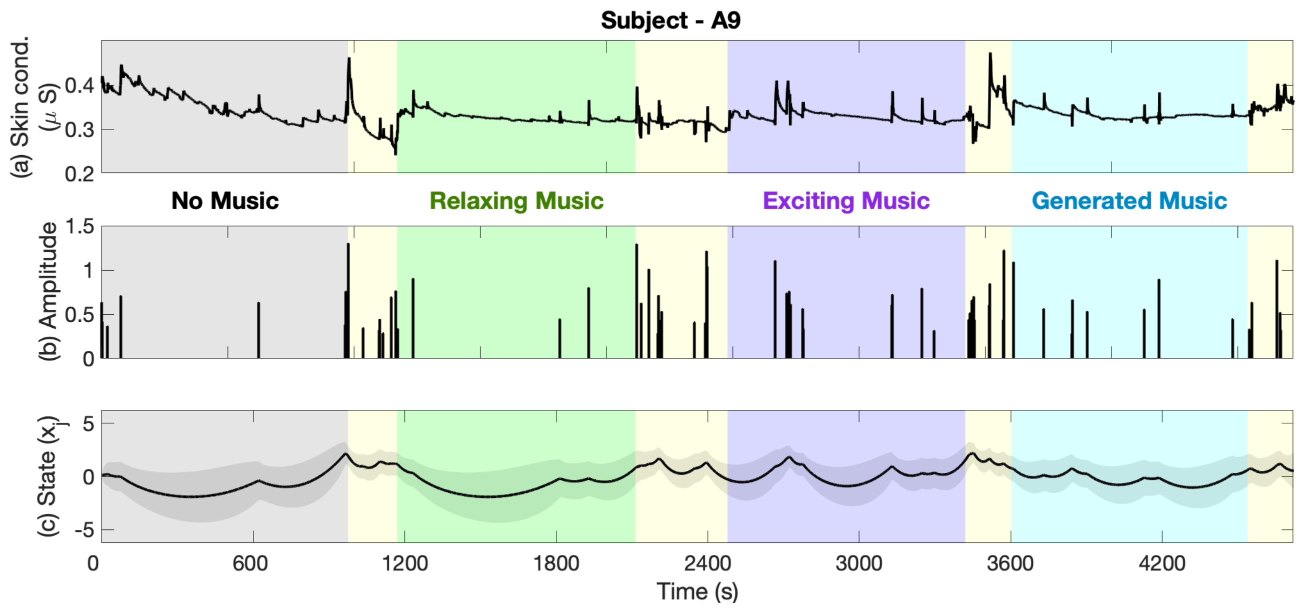

Figure S29. **Cognitive Arousal Results (Subject - A9, Experiment 1).** Panels from the top show in turn the skin conductance signal, underlying neural impulses, and estimated cognitive arousal state. The grey, green, purple, and blue background colors represent the results associated with no music, relaxing music, exciting music, and newly generated relaxing music sessions, respectively. Yellow backgrounds are associated with rest time periods.

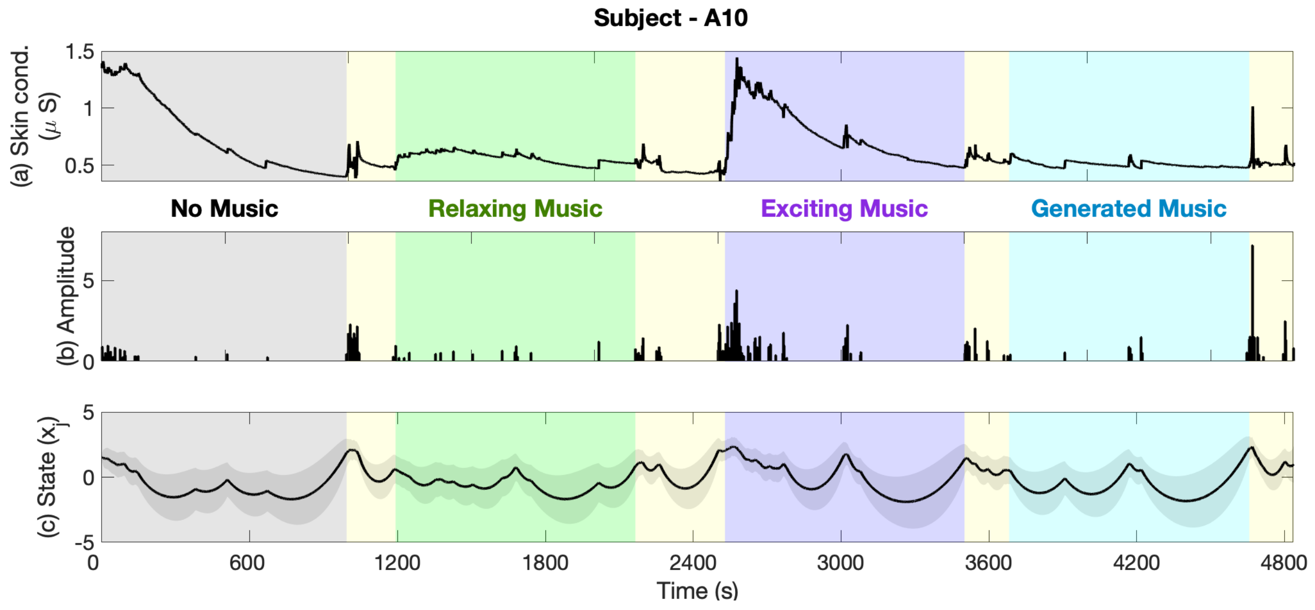

Figure S30. **Cognitive Arousal Results (Subject - A10, Experiment 1).** Panels from the top show in turn the skin conductance signal, underlying neural impulses, and estimated cognitive arousal state. The grey, green, purple, and blue background colors represent the results associated with no music, relaxing music, exciting music, and newly generated relaxing music sessions, respectively. Yellow backgrounds are associated with rest time periods.

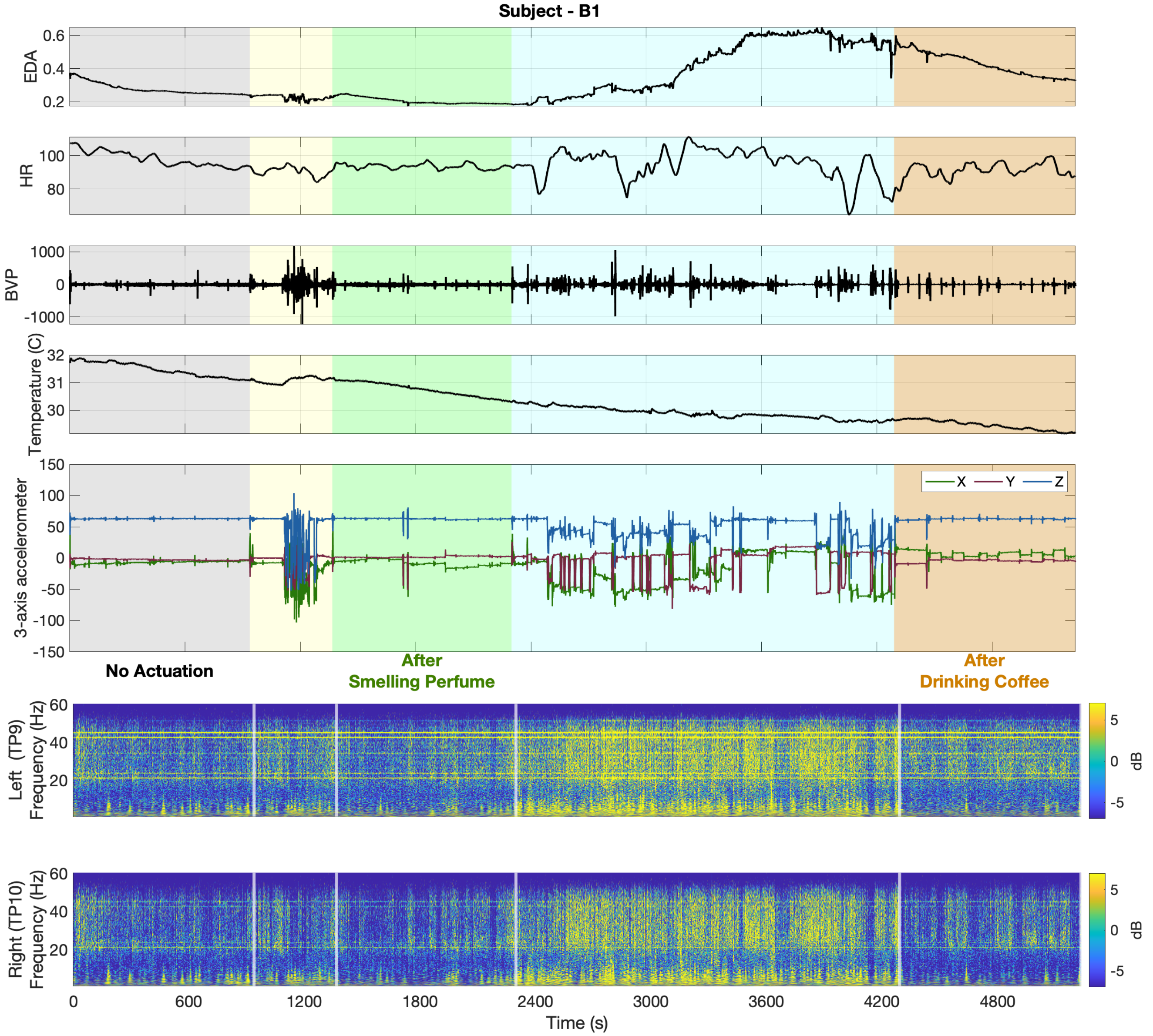

Figure S31. Raw Physiological Data Collected via Empatica E4 Wristband and Muse Headband (Subject - B1, Experiment 2). In the top panel, sub-panels in turn represent the EDA, HR, BVP, body temperature, and 3-axis accelerometer data. The grey, green, and rust background colors in turn represent the results associated with no actuation, smelling perfume, and drinking coffee sessions, respectively. Yellow and blue background colors are associated with rest time periods for smelling perfume and drinking coffee, respectively. Sub-panels in the bottom panel depict spectrogram representations of EEG signals recorded from the left and right temporoparietal areas of the brain (i.e., TP9 and TP10). White vertical lines separate the memory tasks from rest times.

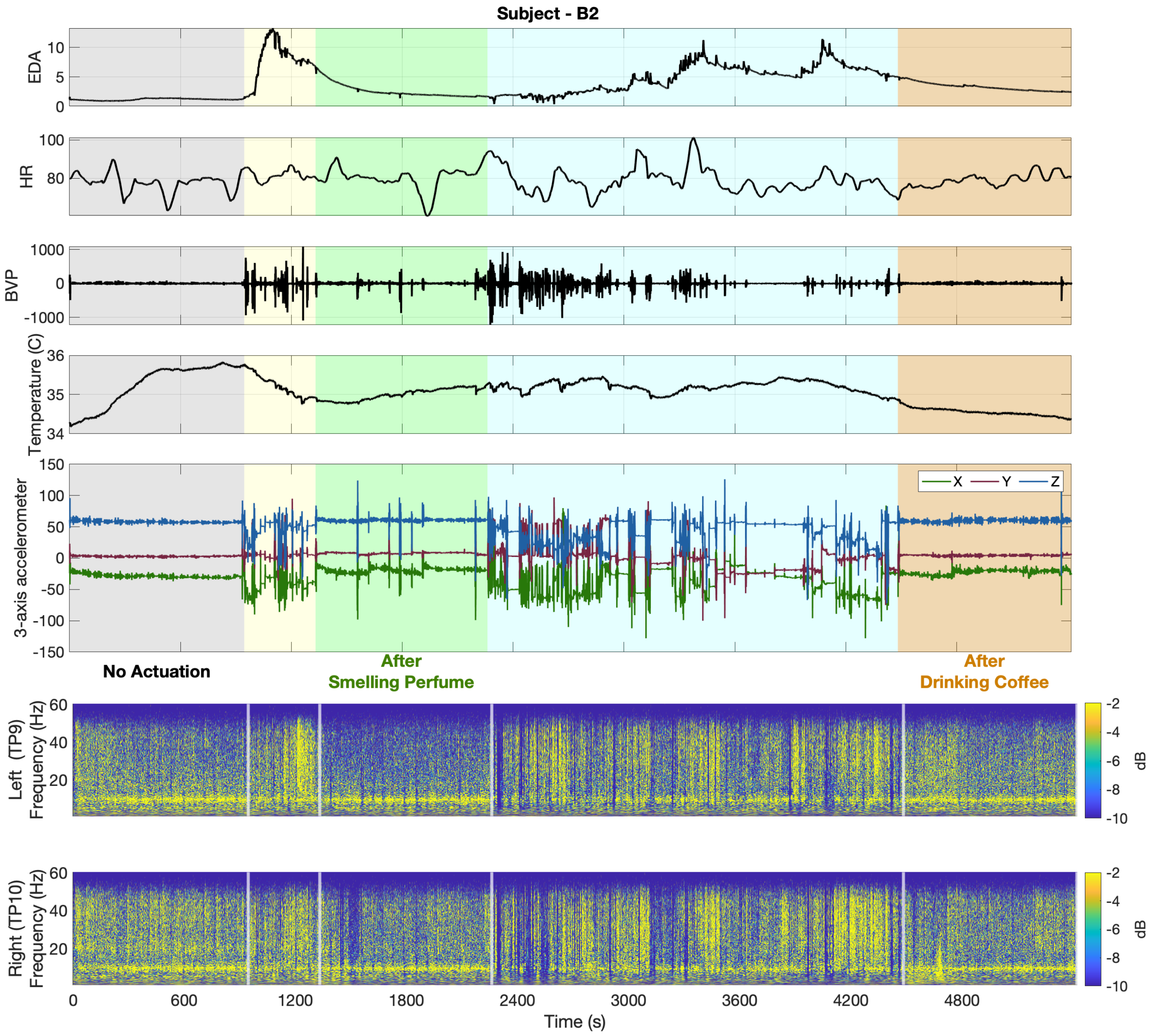

Figure S32. Raw Physiological Data Collected via Empatica E4 Wristband and Muse Headband (Subject - B2, Experiment 2). In the top panel, sub-panels in turn represent the EDA, HR, BVP, body temperature, and 3-axis accelerometer data. The grey, green, and rust background colors in turn represent the results associated with no actuation, smelling perfume, and drinking coffee sessions, respectively. Yellow and blue background colors are associated with rest time periods for smelling perfume and drinking coffee, respectively. Sub-panels in the bottom panel depict spectrogram representations of EEG signals recorded from the left and right temporoparietal areas of the brain (i.e., TP9 and TP10). White vertical lines separate the memory tasks from rest times.

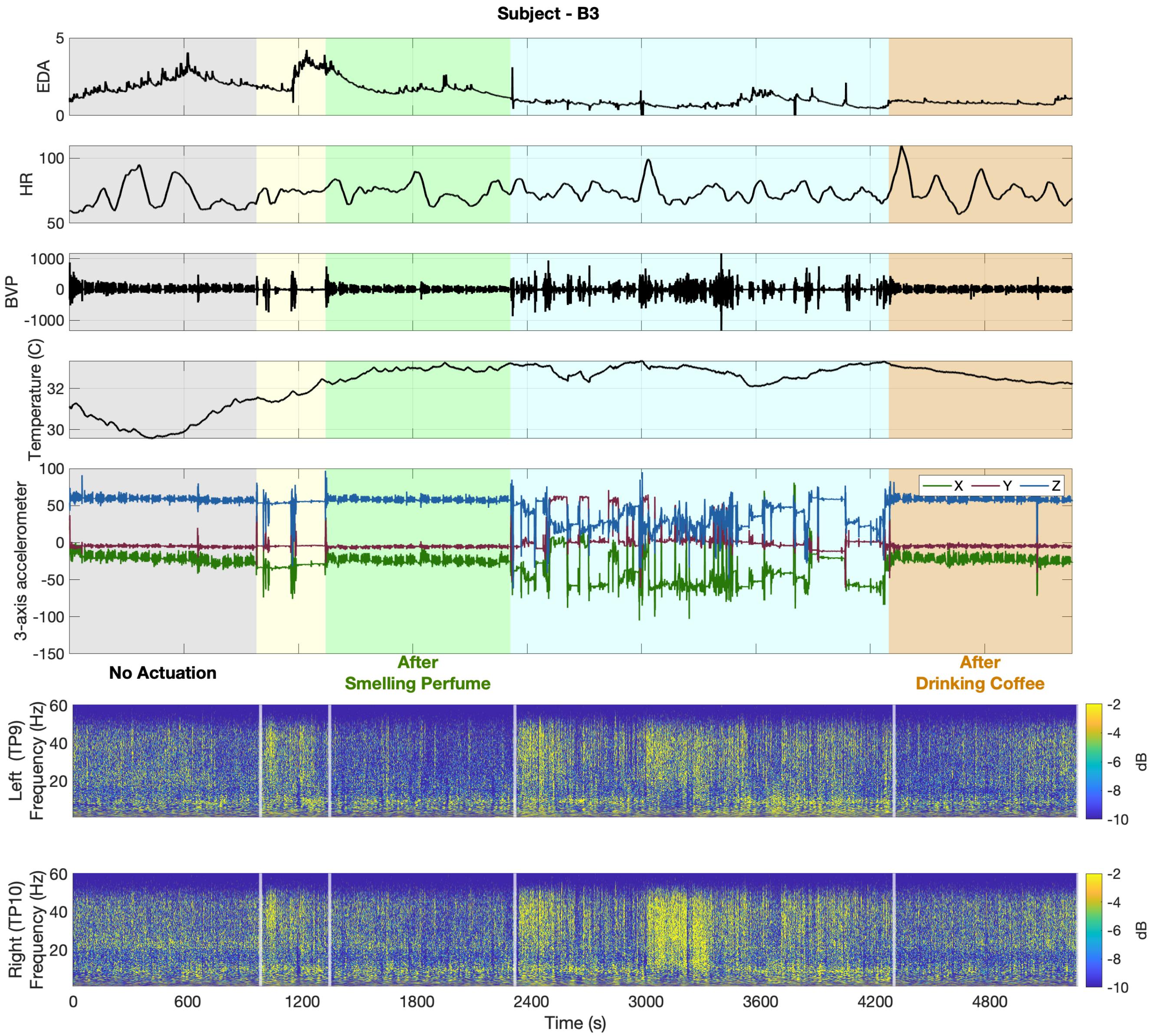

Figure S33. Raw Physiological Data Collected via Empatica E4 Wristband and Muse Headband (Subject - B3, Experiment 2). In the top panel, sub-panels in turn represent the EDA, HR, BVP, body temperature, and 3-axis accelerometer data. The grey, green, and rust background colors in turn represent the results associated with no actuation, smelling perfume, and drinking coffee sessions, respectively. Yellow and blue background colors are associated with rest time periods for smelling perfume and drinking coffee, respectively. Sub-panels in the bottom panel depict spectrogram representations of EEG signals recorded from the left and right temporoparietal areas of the brain (i.e., TP9 and TP10). White vertical lines separate the memory tasks from rest times.

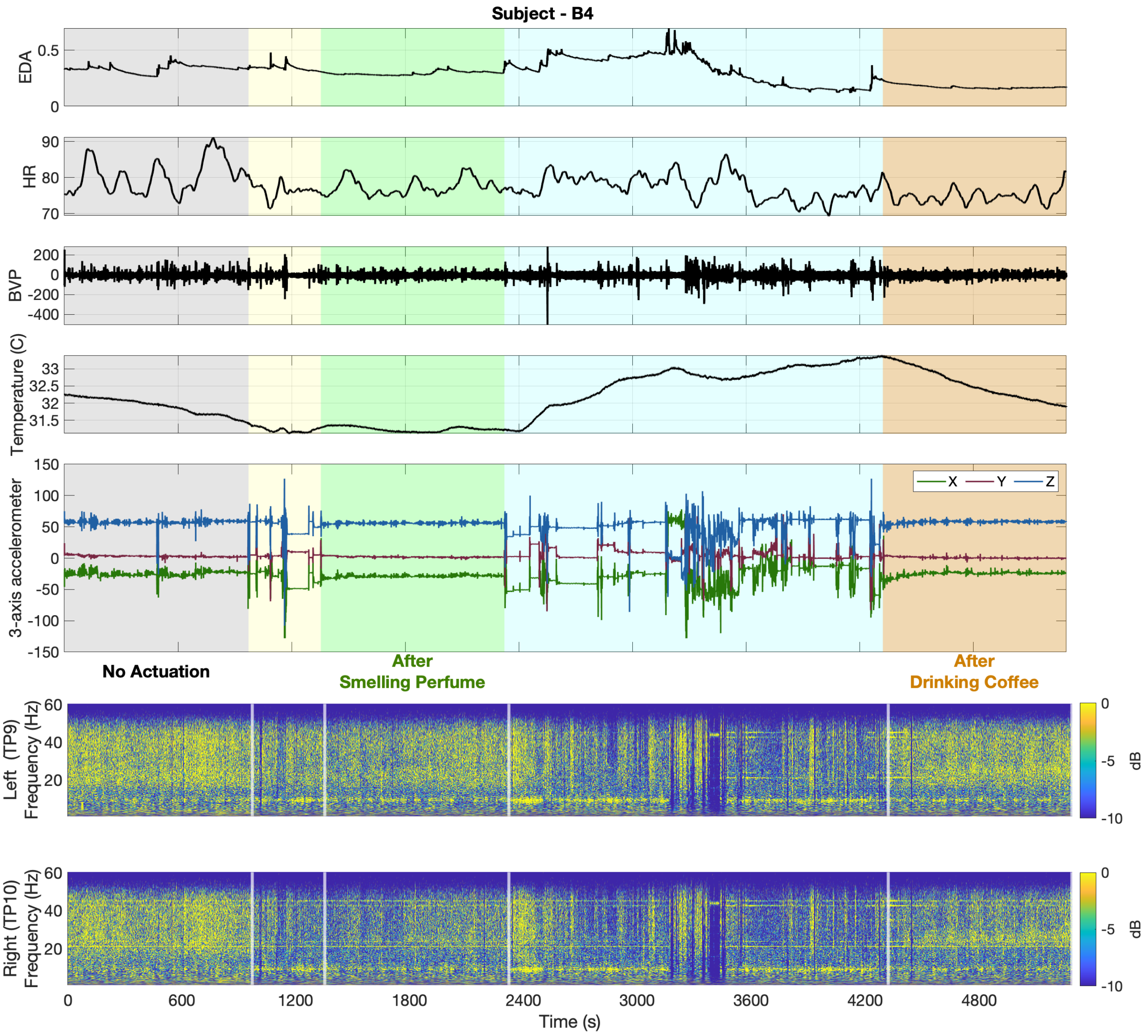

Figure S34. Raw Physiological Data Collected via Empatica E4 Wristband and Muse Headband (Subject - B4, Experiment 2). In the top panel, sub-panels in turn represent the EDA, HR, BVP, body temperature, and 3-axis accelerometer data. The grey, green, and rust background colors in turn represent the results associated with no actuation, smelling perfume, and drinking coffee sessions, respectively. Yellow and blue background colors are associated with rest time periods for smelling perfume and drinking coffee, respectively. Sub-panels in the bottom panel depict spectrogram representations of EEG signals recorded from the left and right temporoparietal areas of the brain (i.e., TP9 and TP10). White vertical lines separate the memory tasks from rest times.

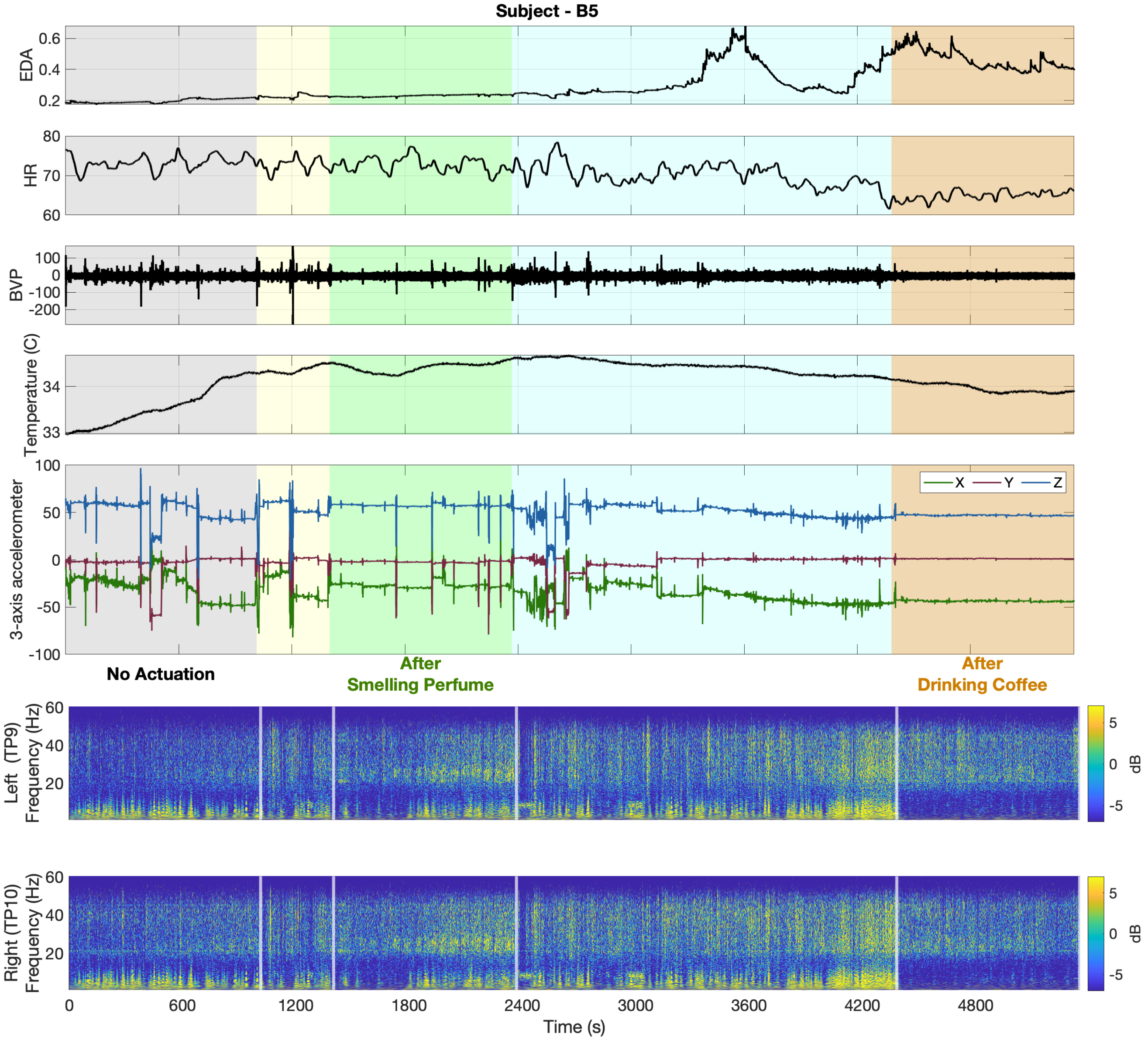

Figure S35. Raw Physiological Data Collected via Empatica E4 Wristband and Muse Headband (Subject - B5, Experiment 2). In the top panel, sub-panels in turn represent the EDA, HR, BVP, body temperature, and 3-axis accelerometer data. The grey, green, and rust background colors in turn represent the results associated with no actuation, smelling perfume, and drinking coffee sessions, respectively. Yellow and blue background colors are associated with rest time periods for smelling perfume and drinking coffee, respectively. Sub-panels in the bottom panel depict spectrogram representations of EEG signals recorded from the left and right temporoparietal areas of the brain (i.e., TP9 and TP10). White vertical lines separate the memory tasks from rest times.

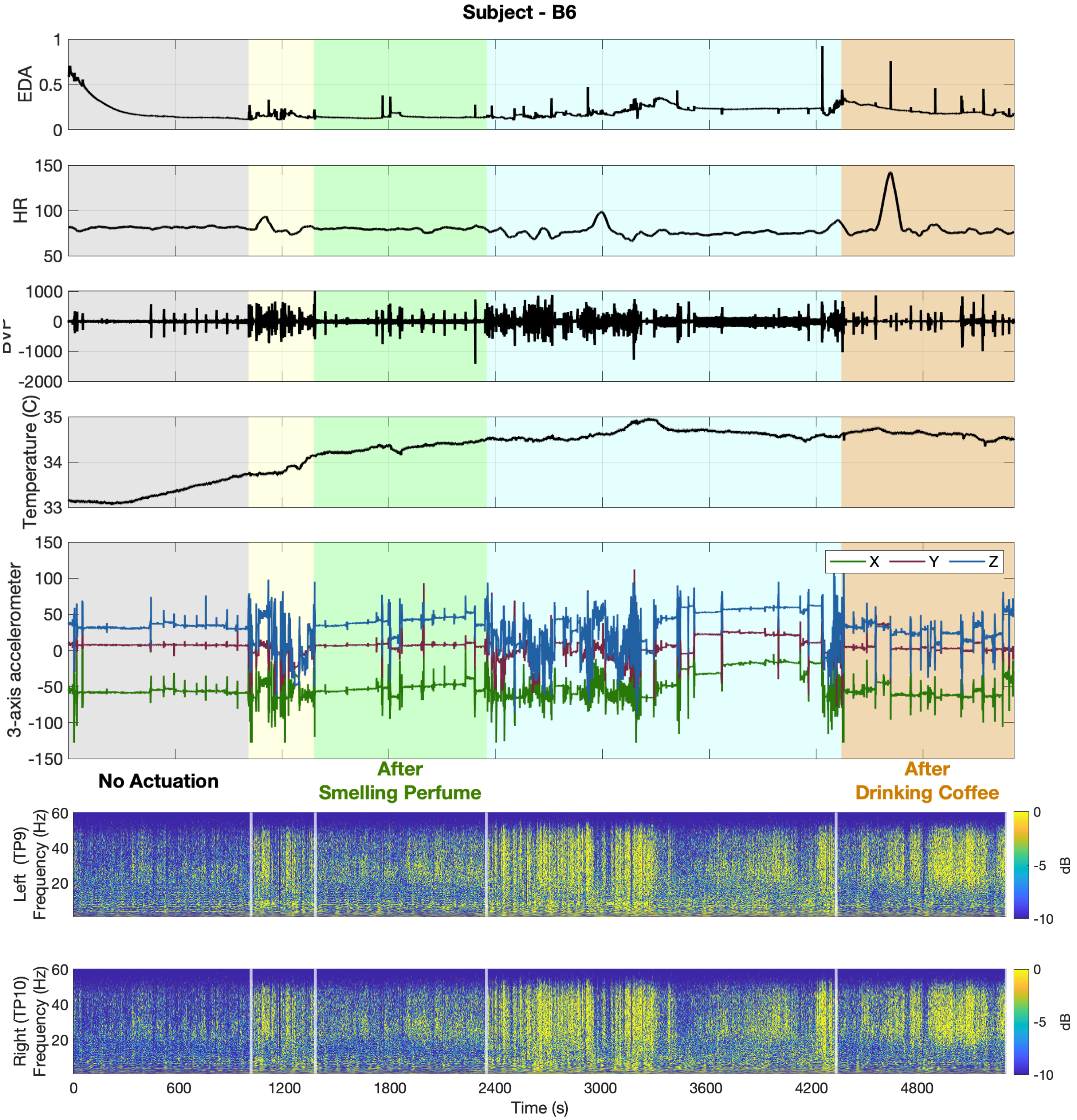

Figure S36. Raw Physiological Data Collected via Empatica E4 Wristband and Muse Headband (Subject - B6, Experiment 2). In the top panel, sub-panels in turn represent the EDA, HR, BVP, body temperature, and 3-axis accelerometer data. The grey, green, and rust background colors in turn represent the results associated with no actuation, smelling perfume, and drinking coffee sessions, respectively. Yellow and blue background colors are associated with rest time periods for smelling perfume and drinking coffee, respectively. Sub-panels in the bottom panel depict spectrogram representations of EEG signals recorded from the left and right temporoparietal areas of the brain (i.e., TP9 and TP10). White vertical lines separate the memory tasks from rest times.

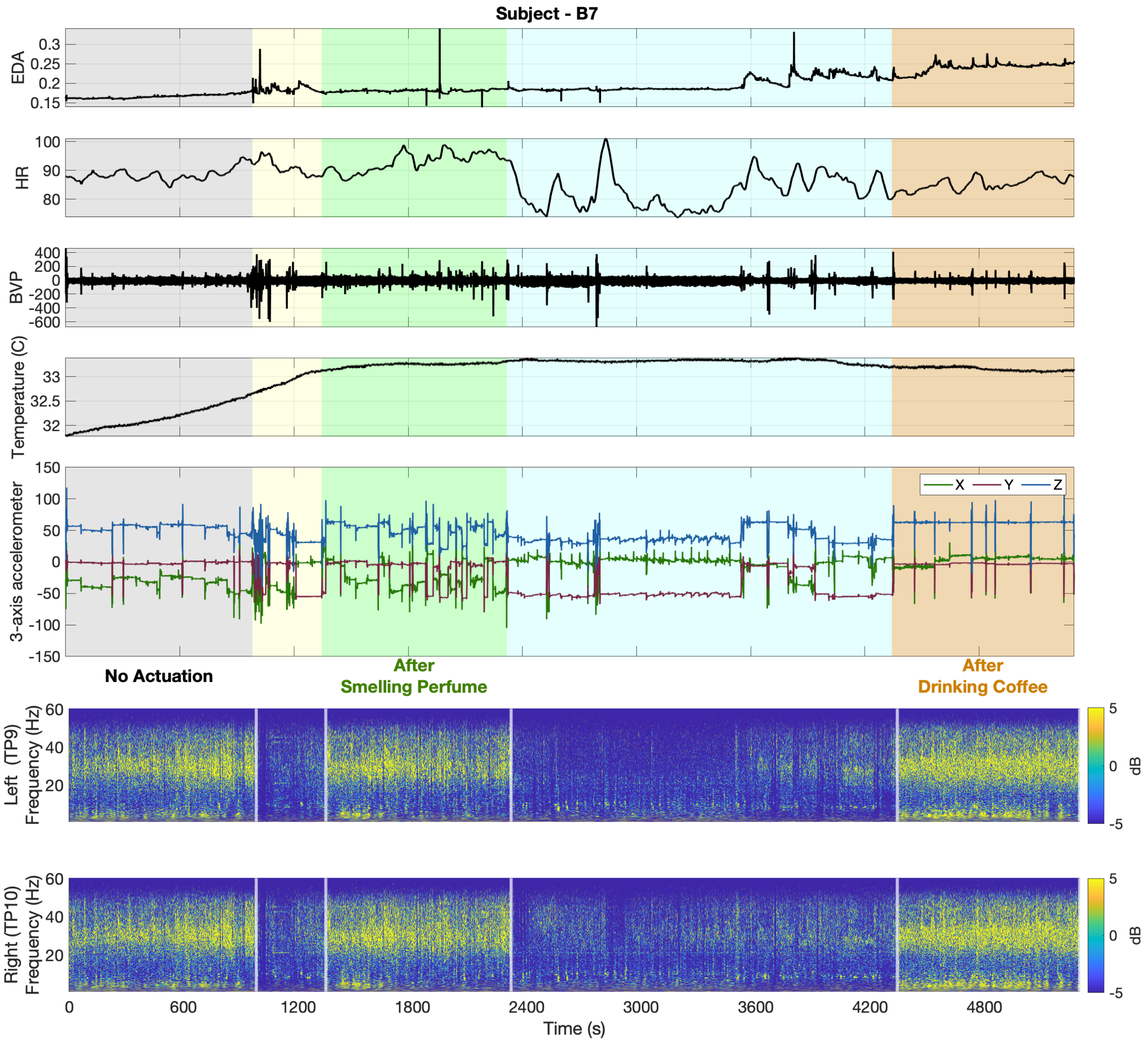

Figure S37. Raw Physiological Data Collected via Empatica E4 Wristband and Muse Headband (Subject - B7, Experiment 2). In the top panel, sub-panels in turn represent the EDA, HR, BVP, body temperature, and 3-axis accelerometer data. The grey, green, and rust background colors in turn represent the results associated with no actuation, smelling perfume, and drinking coffee sessions, respectively. Yellow and blue background colors are associated with rest time periods for smelling perfume and drinking coffee, respectively. Sub-panels in the bottom panel depict spectrogram representations of EEG signals recorded from the left and right temporoparietal areas of the brain (i.e., TP9 and TP10). White vertical lines separate the memory tasks from rest times.

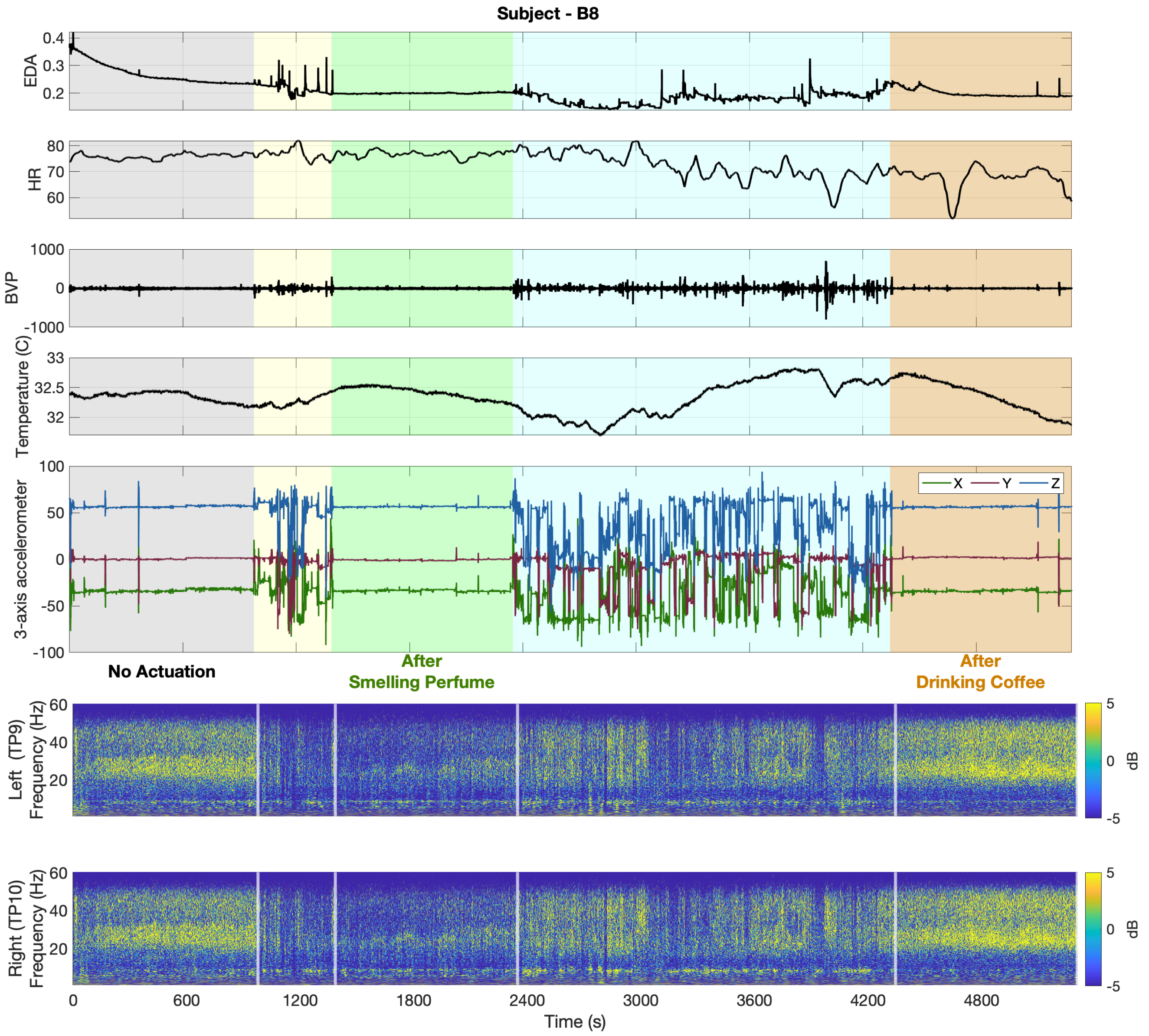

Figure S38. Raw Physiological Data Collected via Empatica E4 Wristband and Muse Headband (Subject - B8, Experiment 2). In the top panel, sub-panels in turn represent the EDA, HR, BVP, body temperature, and 3-axis accelerometer data. The grey, green, and rust background colors in turn represent the results associated with no actuation, smelling perfume, and drinking coffee sessions, respectively. Yellow and blue background colors are associated with rest time periods for smelling perfume and drinking coffee, respectively. Sub-panels in the bottom panel depict spectrogram representations of EEG signals recorded from the left and right temporoparietal areas of the brain (i.e., TP9 and TP10). White vertical lines separate the memory tasks from rest times.

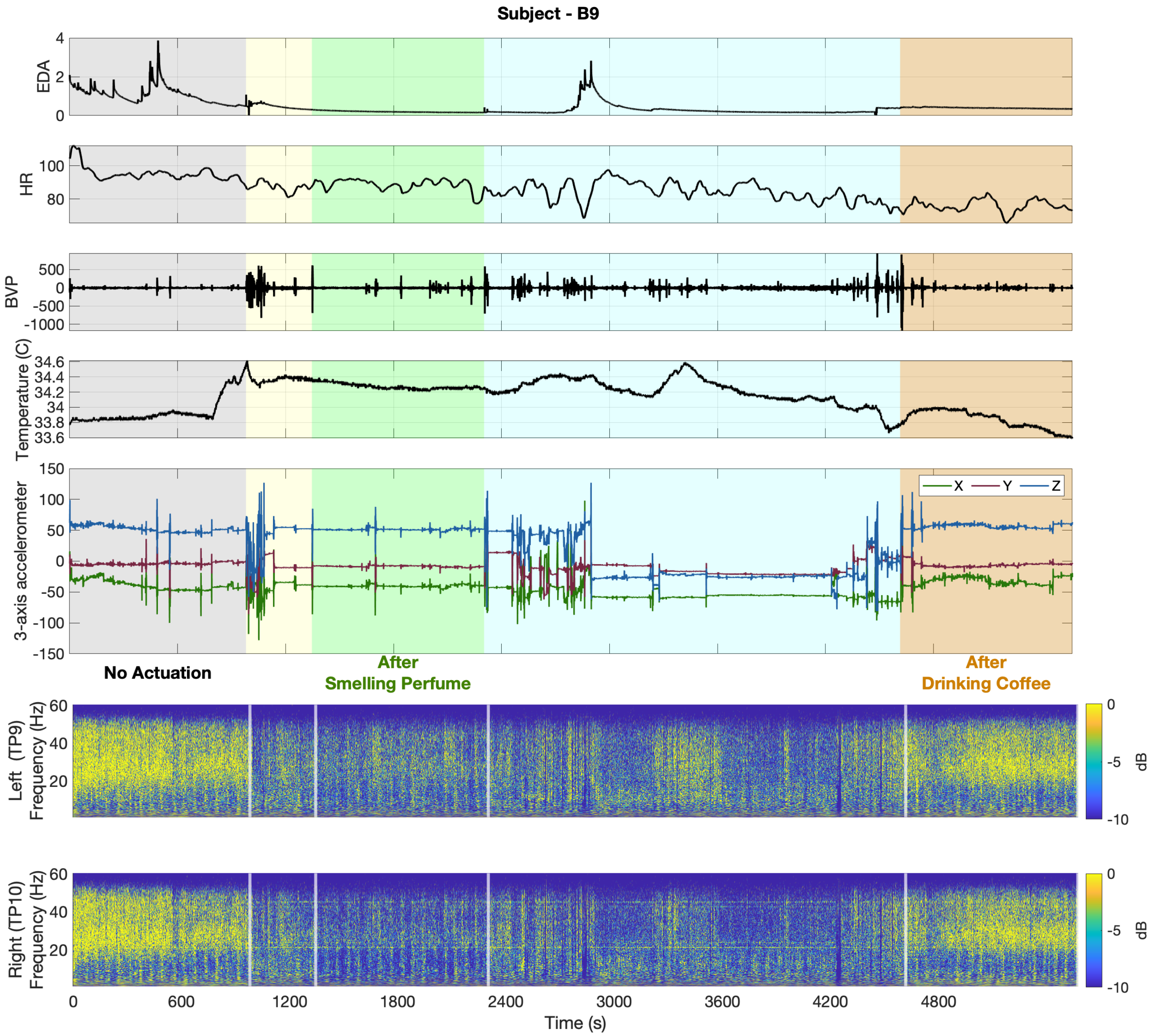

Figure S39. Raw Physiological Data Collected via Empatica E4 Wristband and Muse Headband (Subject - B9, Experiment 2). In the top panel, sub-panels in turn represent the EDA, HR, BVP, body temperature, and 3-axis accelerometer data. The grey, green, and rust background colors in turn represent the results associated with no actuation, smelling perfume, and drinking coffee sessions, respectively. Yellow and blue background colors are associated with rest time periods for smelling perfume and drinking coffee, respectively. Sub-panels in the bottom panel depict spectrogram representations of EEG signals recorded from the left and right temporoparietal areas of the brain (i.e., TP9 and TP10). White vertical lines separate the memory tasks from rest times.

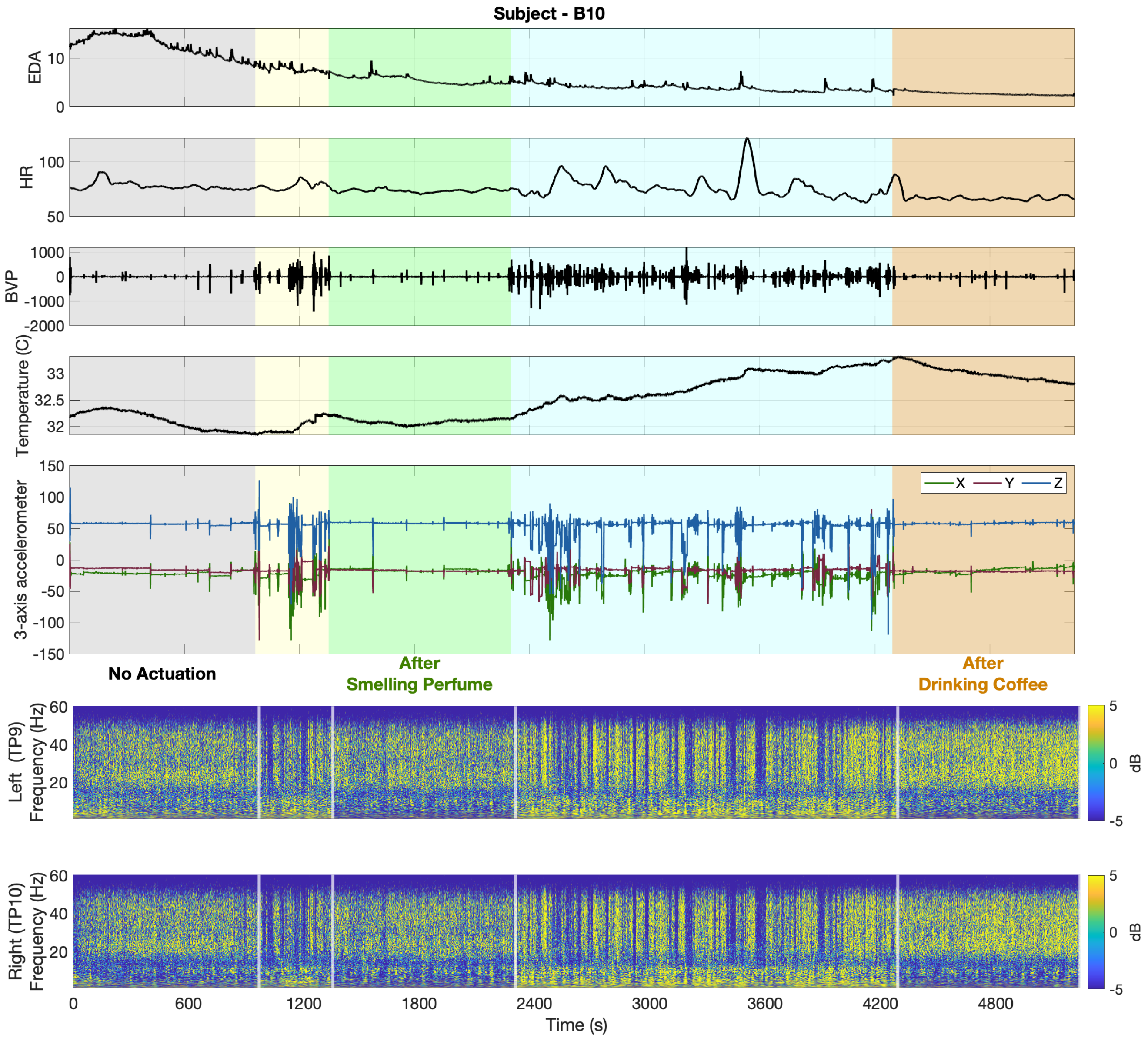

Figure S40. Raw Physiological Data Collected via Empatica E4 Wristband and Muse Headband (Subject - B10, Experiment 2). In the top panel, sub-panels in turn represent the EDA, HR, BVP, body temperature, and 3-axis accelerometer data. The grey, green, and rust background colors in turn represent the results associated with no actuation, smelling perfume, and drinking coffee sessions, respectively. Yellow and blue background colors are associated with rest time periods for smelling perfume and drinking coffee, respectively. Sub-panels in the bottom panel depict spectrogram representations of EEG signals recorded from the left and right temporoparietal areas of the brain (i.e., TP9 and TP10). White vertical lines separate the memory tasks from rest times.

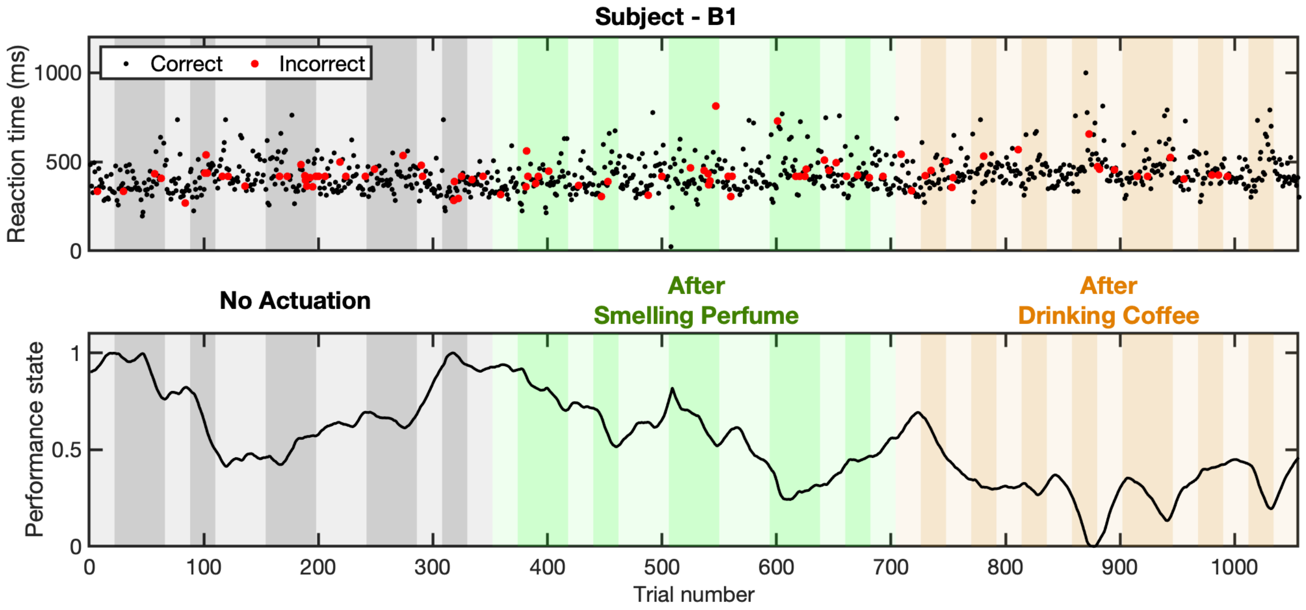

Figure S41. **Cognitive Performance Results (Subject - B1, Experiment 2).** The top panel shows the reaction time along with correct (black) and incorrect (red) responses. The bottom panel shows cognitive performance state estimates. The grey, green, and rust background colors in turn represent the results associated with no actuation, smelling perfume, and drinking coffee sessions, respectively. Bright and dark backgrounds present 1-back and 3-back tasks, respectively.

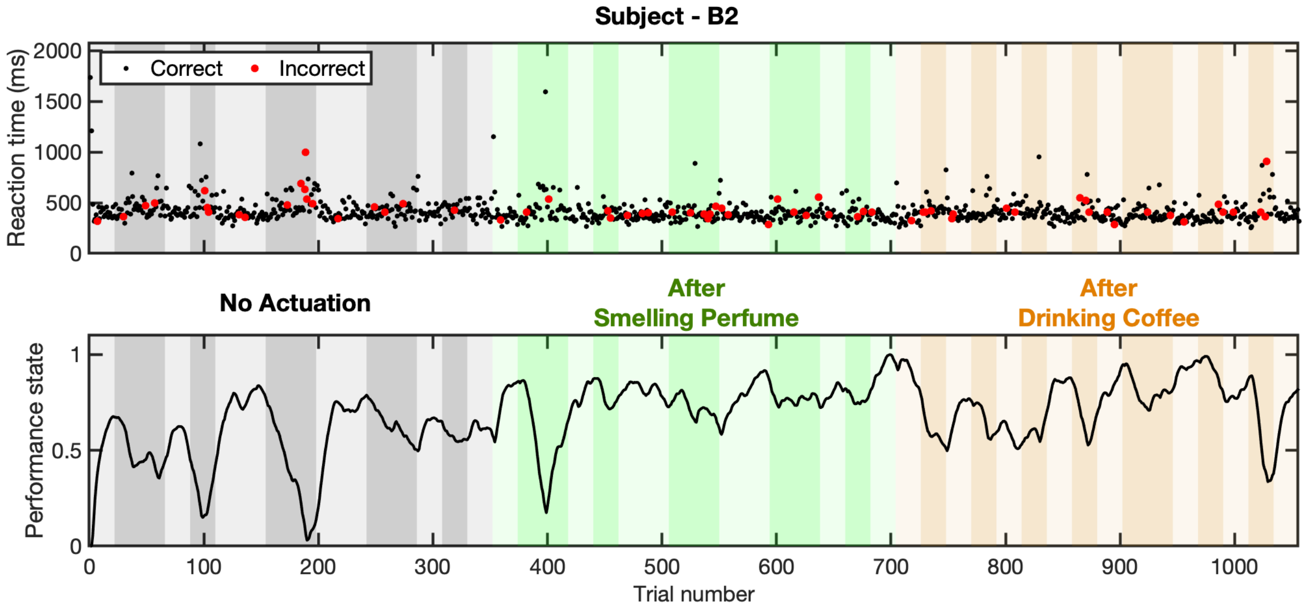

Figure S42. **Cognitive Performance Results (Subject - B2, Experiment 2).** The top panel shows the reaction time along with correct (black) and incorrect (red) responses. The bottom panel shows cognitive performance state estimates. The grey, green, and rust background colors in turn represent the results associated with no actuation, smelling perfume, and drinking coffee sessions, respectively. Bright and dark backgrounds present 1-back and 3-back tasks, respectively.

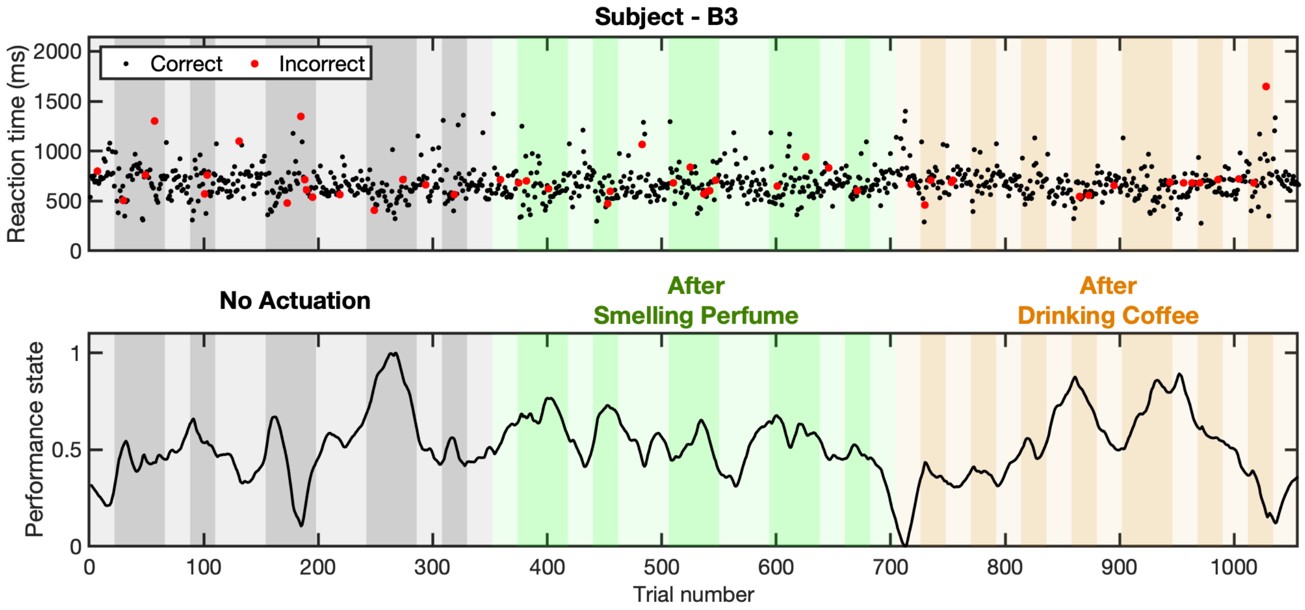

Figure S43. Cognitive Performance Results (Subject - B3, Experiment 2). The top panel shows the reaction time along with correct (black) and incorrect (red) responses. The bottom panel shows cognitive performance state estimates. The grey, green, and rust background colors in turn represent the results associated with no actuation, smelling perfume, and drinking coffee sessions, respectively. Bright and dark backgrounds present 1-back and 3-back tasks, respectively.

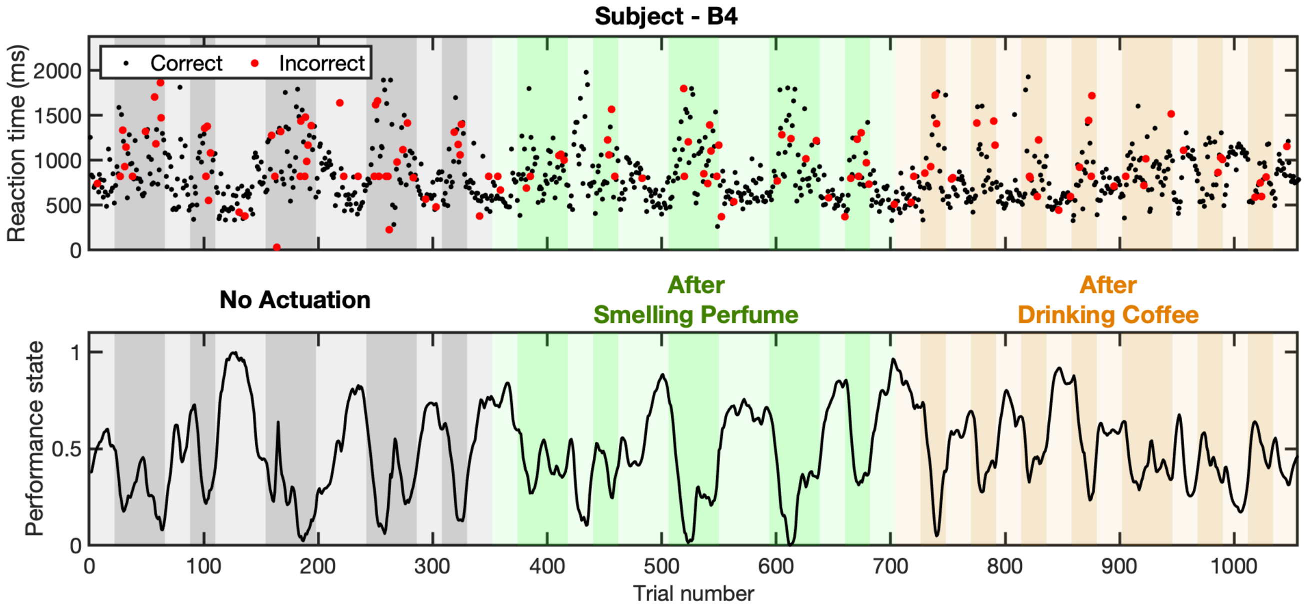

Figure S44. Cognitive Performance Results (Subject - B4, Experiment 2). The top panel shows the reaction time along with correct (black) and incorrect (red) responses. The bottom panel shows cognitive performance state estimates. The grey, green, and rust background colors in turn represent the results associated with no actuation, smelling perfume, and drinking coffee sessions, respectively. Bright and dark backgrounds present 1-back and 3-back tasks, respectively.

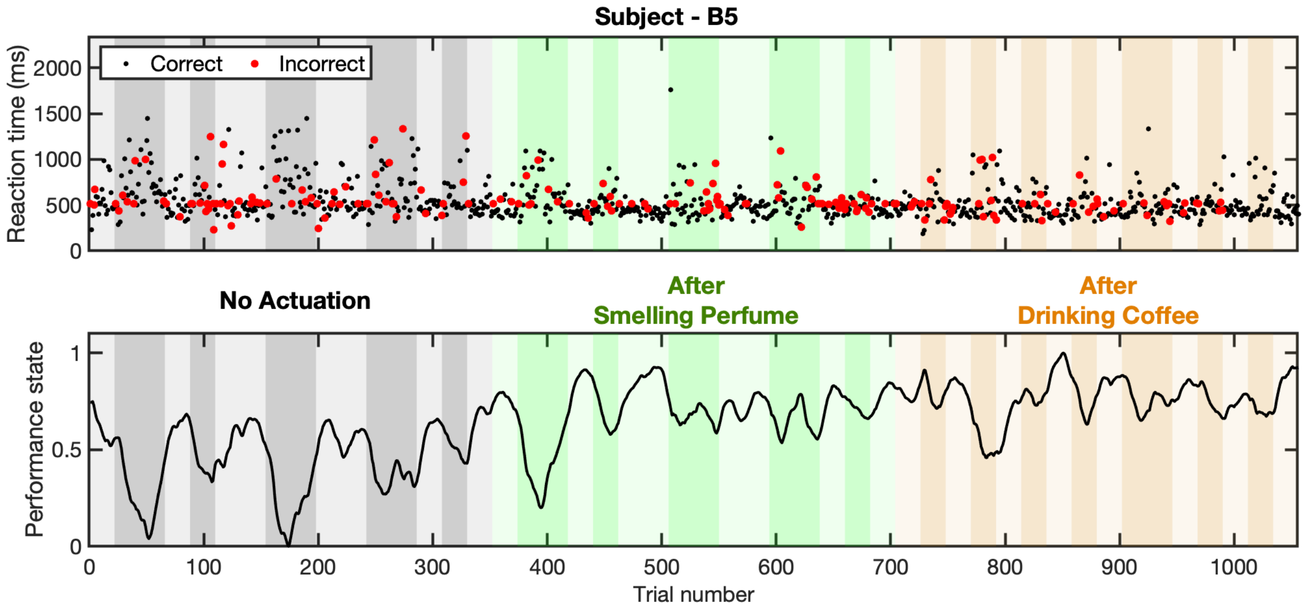

Figure S45. **Cognitive Performance Results (Subject - B5, Experiment 2).** The top panel shows the reaction time along with correct (black) and incorrect (red) responses. The bottom panel shows cognitive performance state estimates. The grey, green, and rust background colors in turn represent the results associated with no actuation, smelling perfume, and drinking coffee sessions, respectively. Bright and dark backgrounds present 1-back and 3-back tasks, respectively.

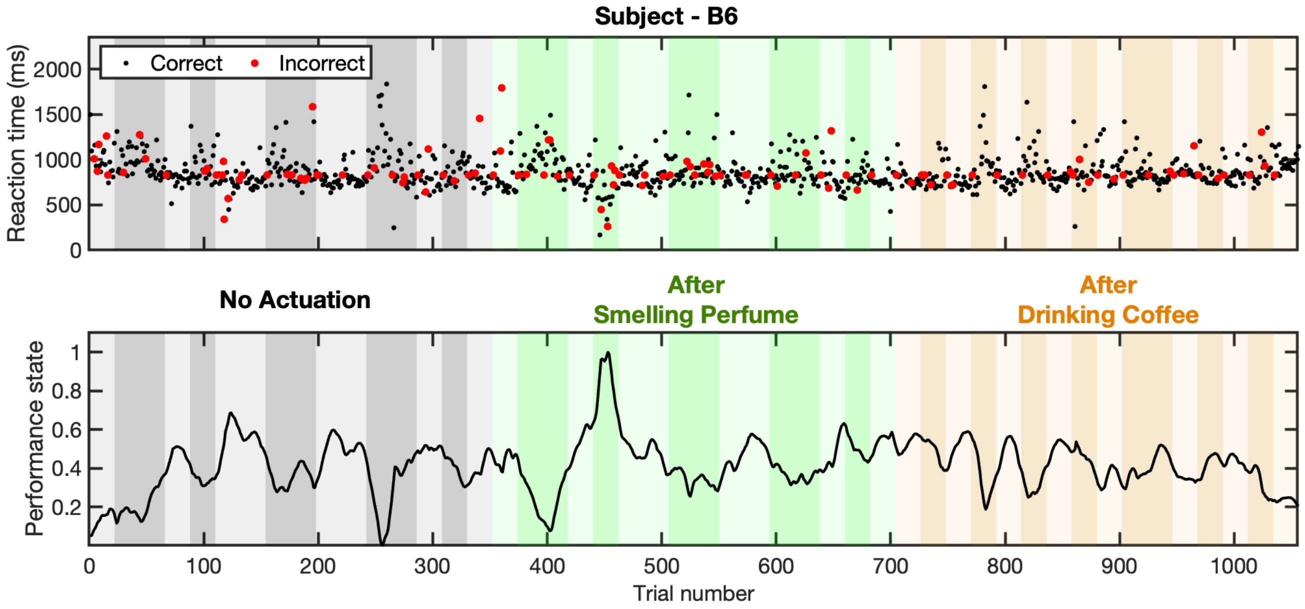

Figure S46. **Cognitive Performance Results (Subject - B6, Experiment 2).** The top panel shows the reaction time along with correct (black) and incorrect (red) responses. The bottom panel shows cognitive performance state estimates. The grey, green, and rust background colors in turn represent the results associated with no actuation, smelling perfume, and drinking coffee sessions, respectively. Bright and dark backgrounds present 1-back and 3-back tasks, respectively.

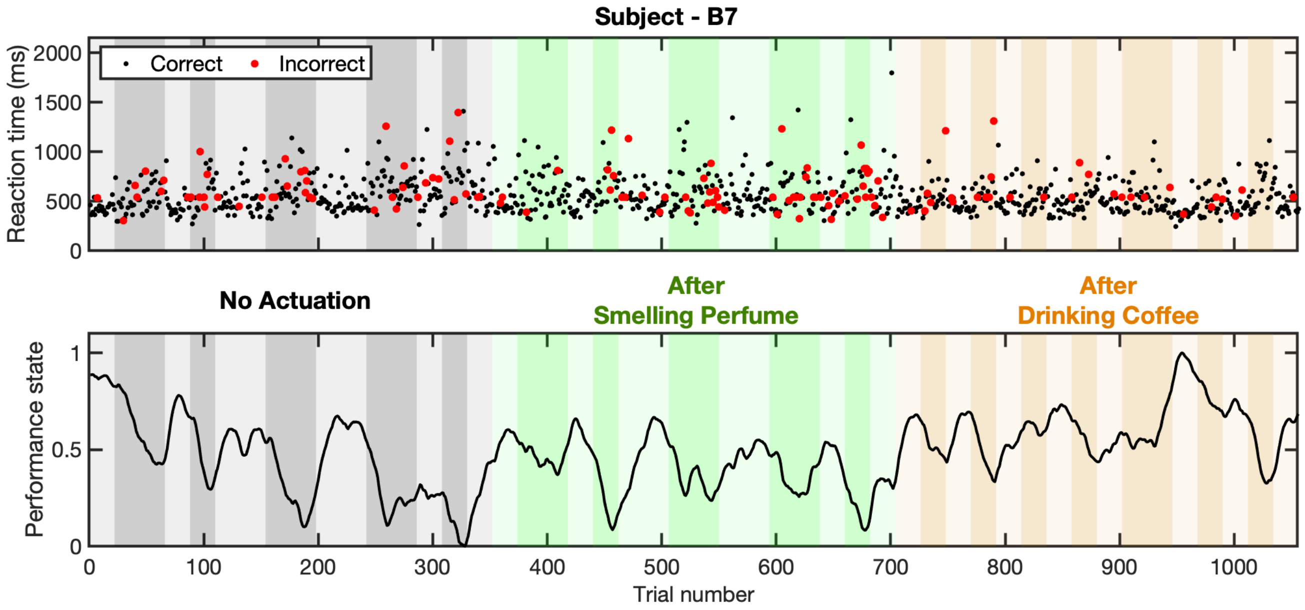

Figure S47. **Cognitive Performance Results (Subject - B7, Experiment 2).** The top panel shows the reaction time along with correct (black) and incorrect (red) responses. The bottom panel shows cognitive performance state estimates. The grey, green, and rust background colors in turn represent the results associated with no actuation, smelling perfume, and drinking coffee sessions, respectively. Bright and dark backgrounds present 1-back and 3-back tasks, respectively.

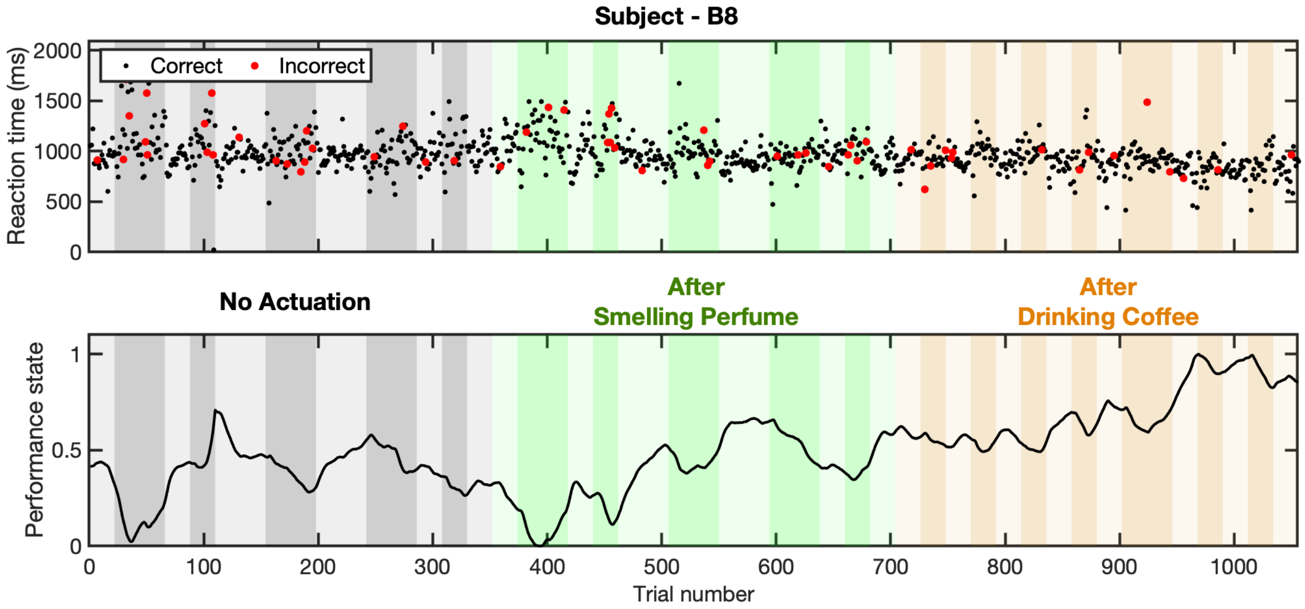

Figure S48. **Cognitive Performance Results (Subject - B8, Experiment 2).** The top panel shows the reaction time along with correct (black) and incorrect (red) responses. The bottom panel shows cognitive performance state estimates. The grey, green, and rust background colors in turn represent the results associated with no actuation, smelling perfume, and drinking coffee sessions, respectively. Bright and dark backgrounds present 1-back and 3-back tasks, respectively.

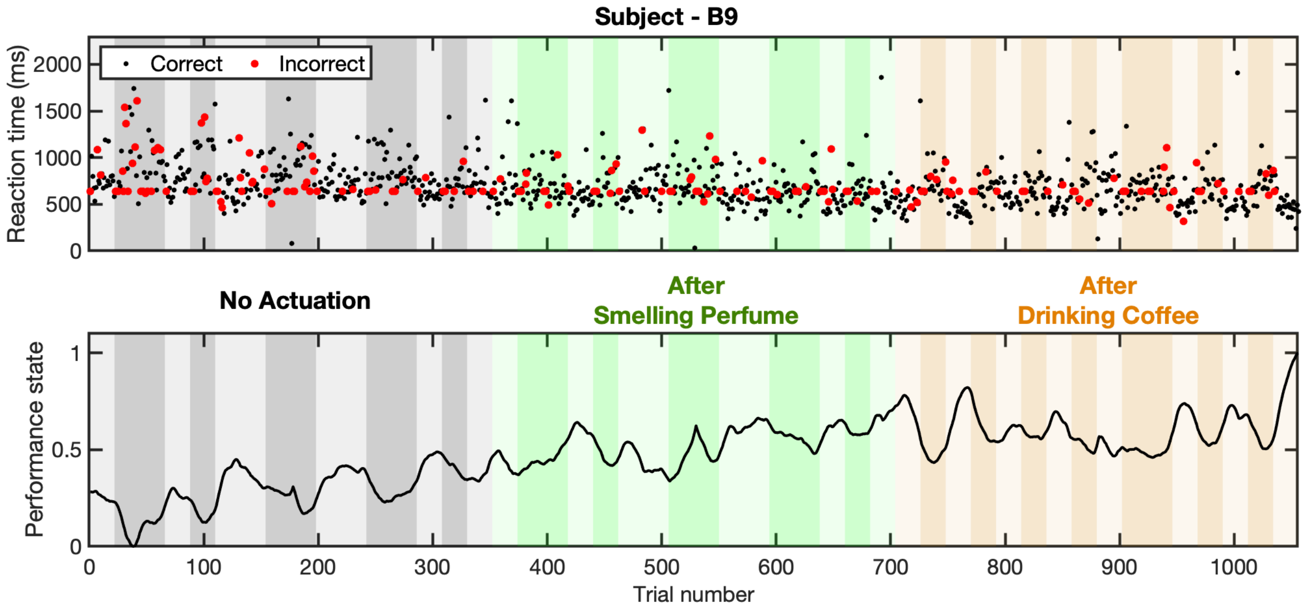

Figure S49. Cognitive Performance Results (Subject - B9, Experiment 2). The top panel shows the reaction time along with correct (black) and incorrect (red) responses. The bottom panel shows cognitive performance state estimates. The grey, green, and rust background colors in turn represent the results associated with no actuation, smelling perfume, and drinking coffee sessions, respectively. Bright and dark backgrounds present 1-back and 3-back tasks, respectively.

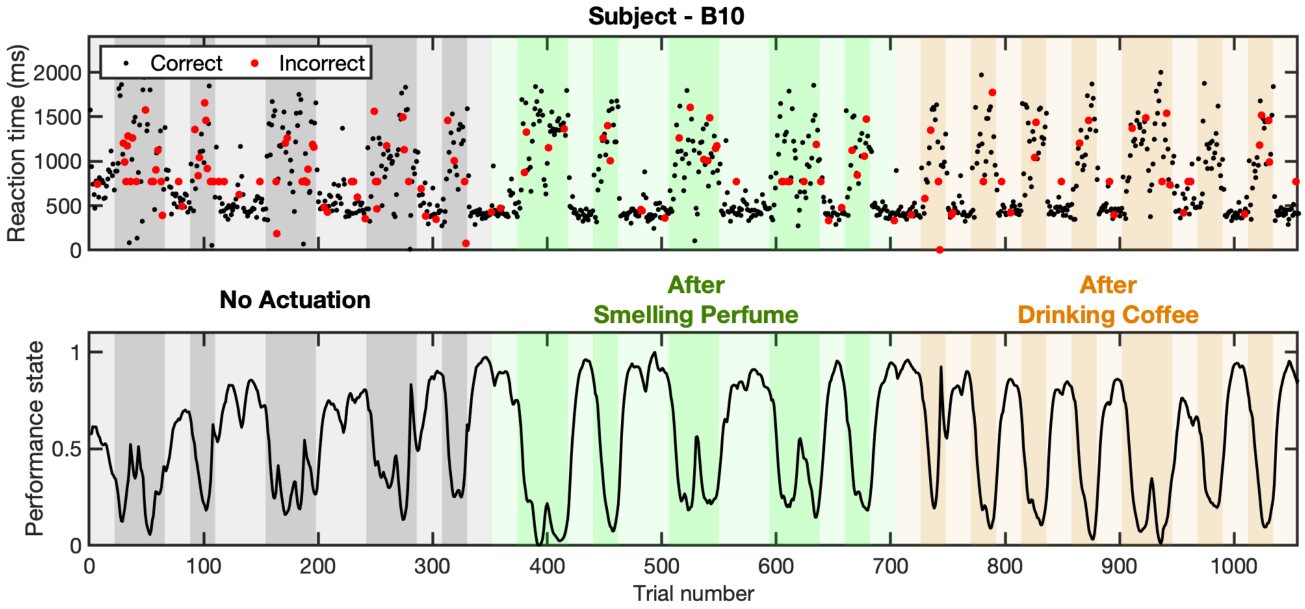

Figure S50. Cognitive Performance Results (Subject - B10, Experiment 2). The top panel shows the reaction time along with correct (black) and incorrect (red) responses. The bottom panel shows cognitive performance state estimates. The grey, green, and rust background colors in turn represent the results associated with no actuation, smelling perfume, and drinking coffee sessions, respectively. Bright and dark backgrounds present 1-back and 3-back tasks, respectively.

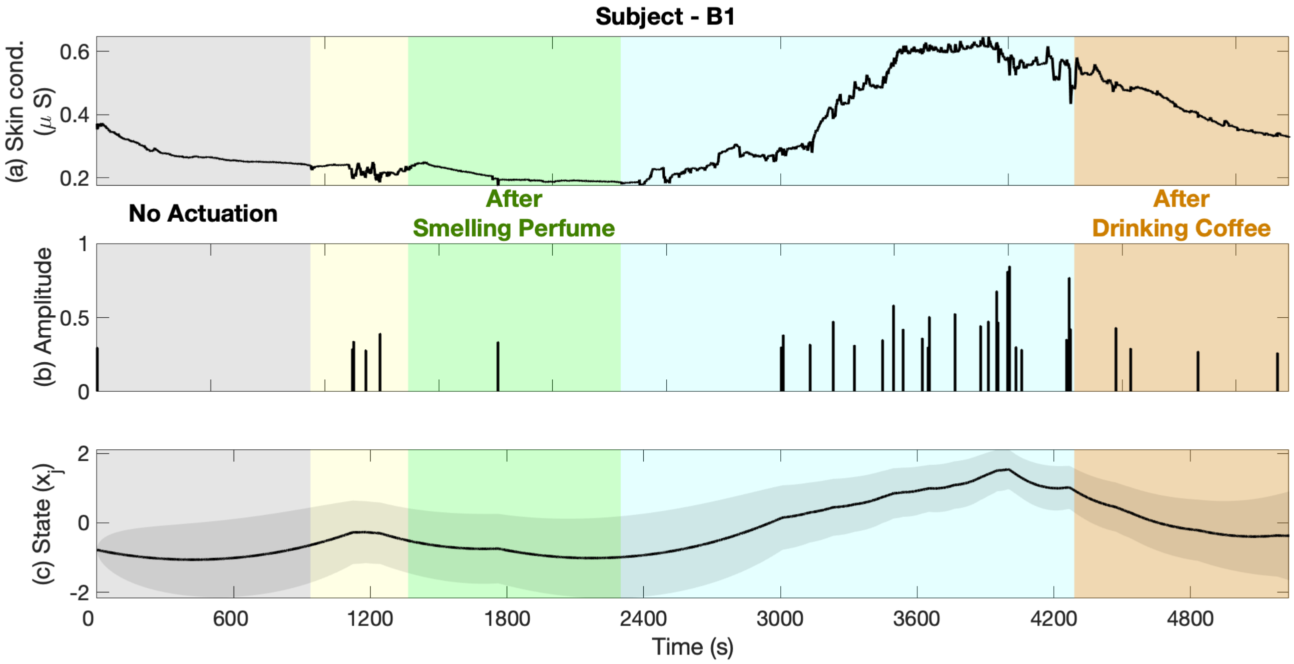

Figure S51. Cognitive Arousal Results (Subject - B1, Experiment 2). Panels from the top show in turn the skin conductance signal, underlying neural impulses, and estimated cognitive arousal state. The grey, green, and rust background colors represent the results associated with no actuation, smelling perfume, and drinking coffee sessions, respectively. Yellow and blue background colors are associated with rest time periods for smelling perfume and drinking coffee, respectively.

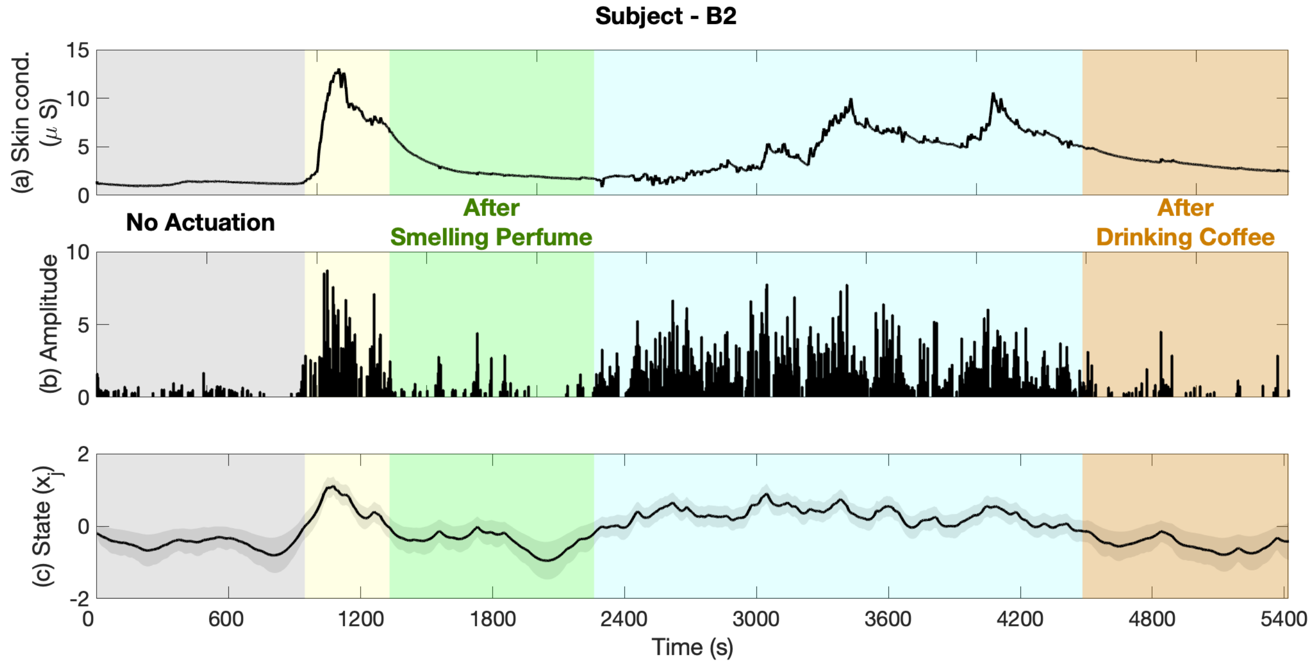

Figure S52. Cognitive Arousal Results (Subject - B2, Experiment 2). Panels from the top show in turn the skin conductance signal, underlying neural impulses, and estimated cognitive arousal state. The grey, green, and rust background colors represent the results associated with no actuation, smelling perfume, and drinking coffee sessions, respectively. Yellow and blue background colors are associated with rest time periods for smelling perfume and drinking coffee, respectively.

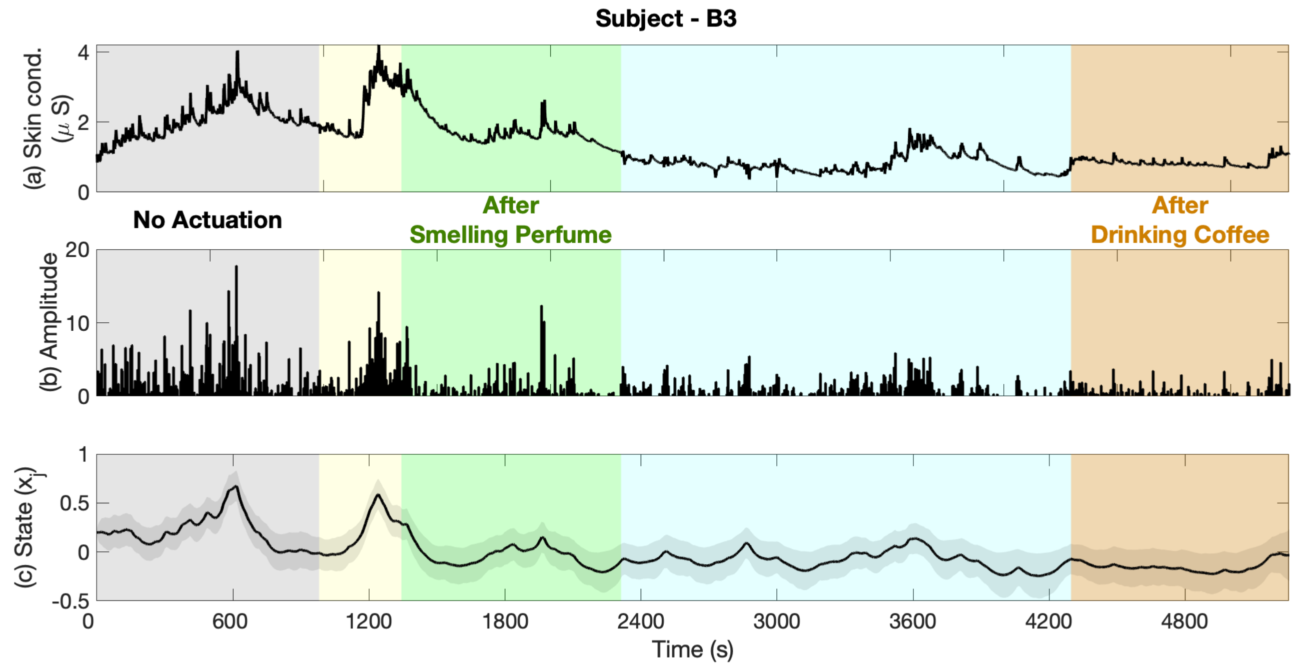

Figure S53. **Cognitive Arousal Results (Subject - B3, Experiment 2).** Panels from the top show in turn the skin conductance signal, underlying neural impulses, and estimated cognitive arousal state. The grey, green, and rust background colors represent the results associated with no actuation, smelling perfume, and drinking coffee sessions, respectively. Yellow and blue background colors are associated with rest time periods for smelling perfume and drinking coffee, respectively.

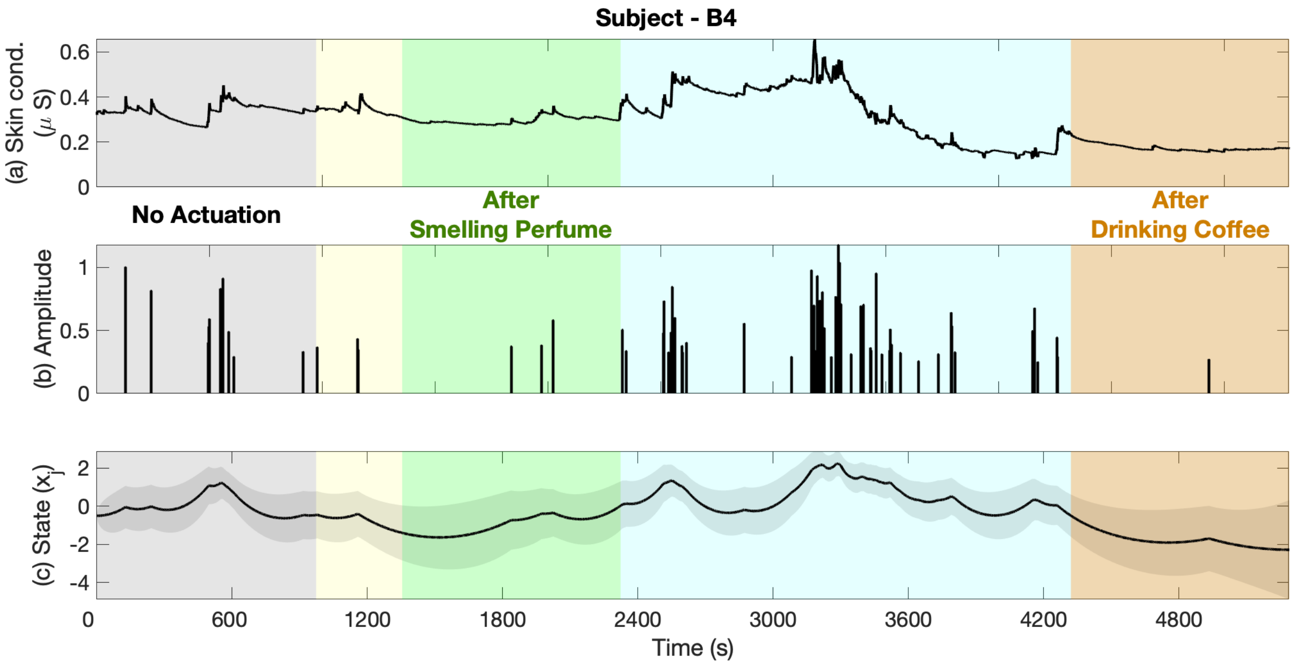

Figure S54. **Cognitive Arousal Results (Subject - B4, Experiment 2).** Panels from the top show in turn the skin conductance signal, underlying neural impulses, and estimated cognitive arousal state. The grey, green, and rust background colors represent the results associated with no actuation, smelling perfume, and drinking coffee sessions, respectively. Yellow and blue background colors are associated with rest time periods for smelling perfume and drinking coffee, respectively.

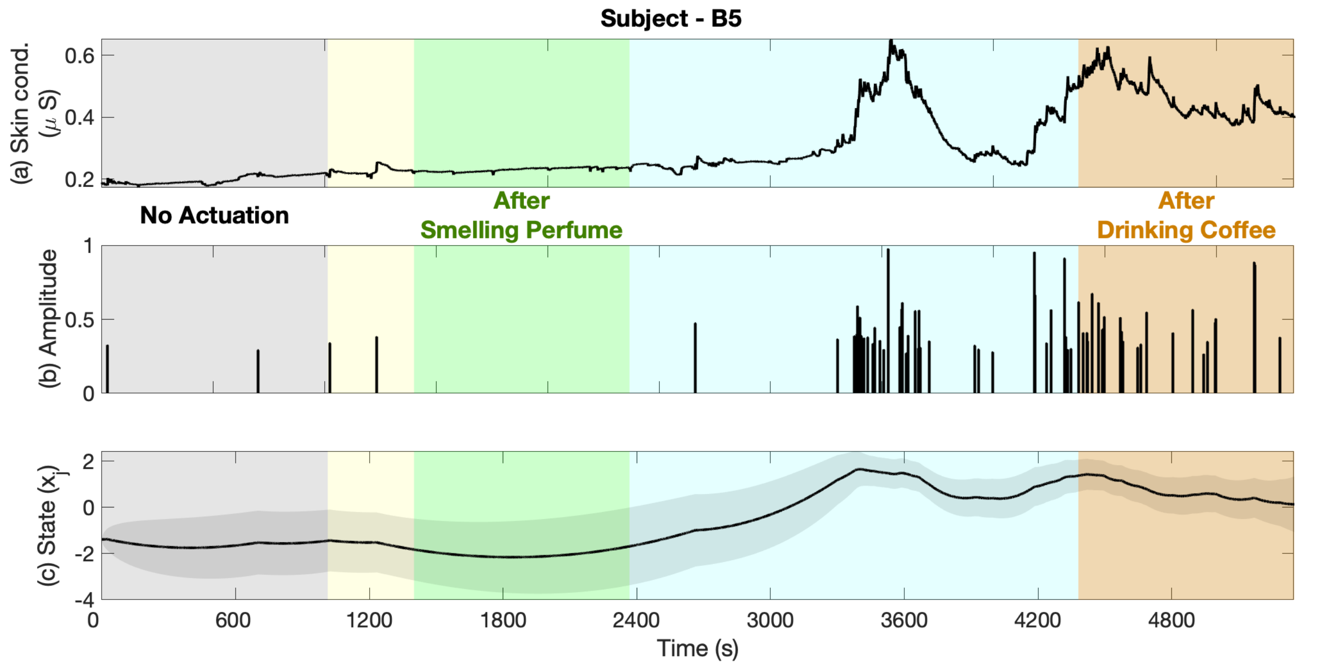

Figure S55. **Cognitive Arousal Results (Subject - B5, Experiment 2).** Panels from the top show in turn the skin conductance signal, underlying neural impulses, and estimated cognitive arousal state. The grey, green, and rust background colors represent the results associated with no actuation, smelling perfume, and drinking coffee sessions, respectively. Yellow and blue background colors are associated with rest time periods for smelling perfume and drinking coffee, respectively.

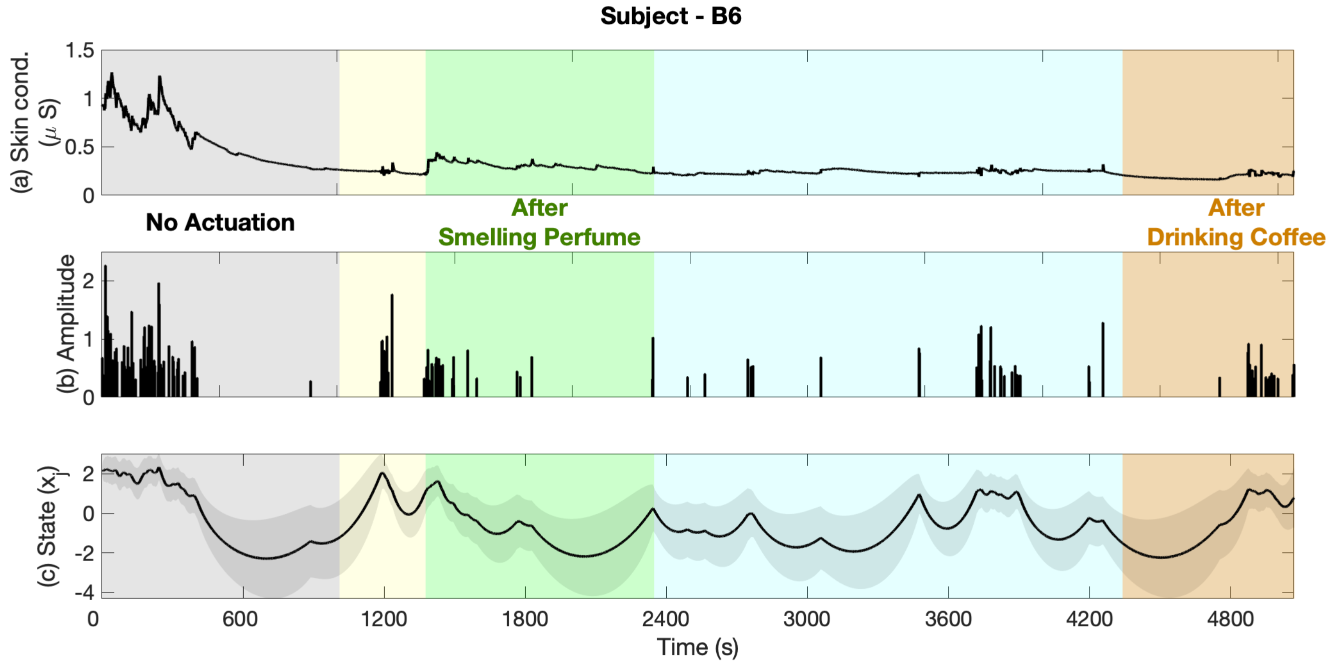

Figure S56. **Cognitive Arousal Results (Subject - B6, Experiment 2).** Panels from the top show in turn the skin conductance signal, underlying neural impulses, and estimated cognitive arousal state. The grey, green, and rust background colors represent the results associated with no actuation, smelling perfume, and drinking coffee sessions, respectively. Yellow and blue background colors are associated with rest time periods for smelling perfume and drinking coffee, respectively.

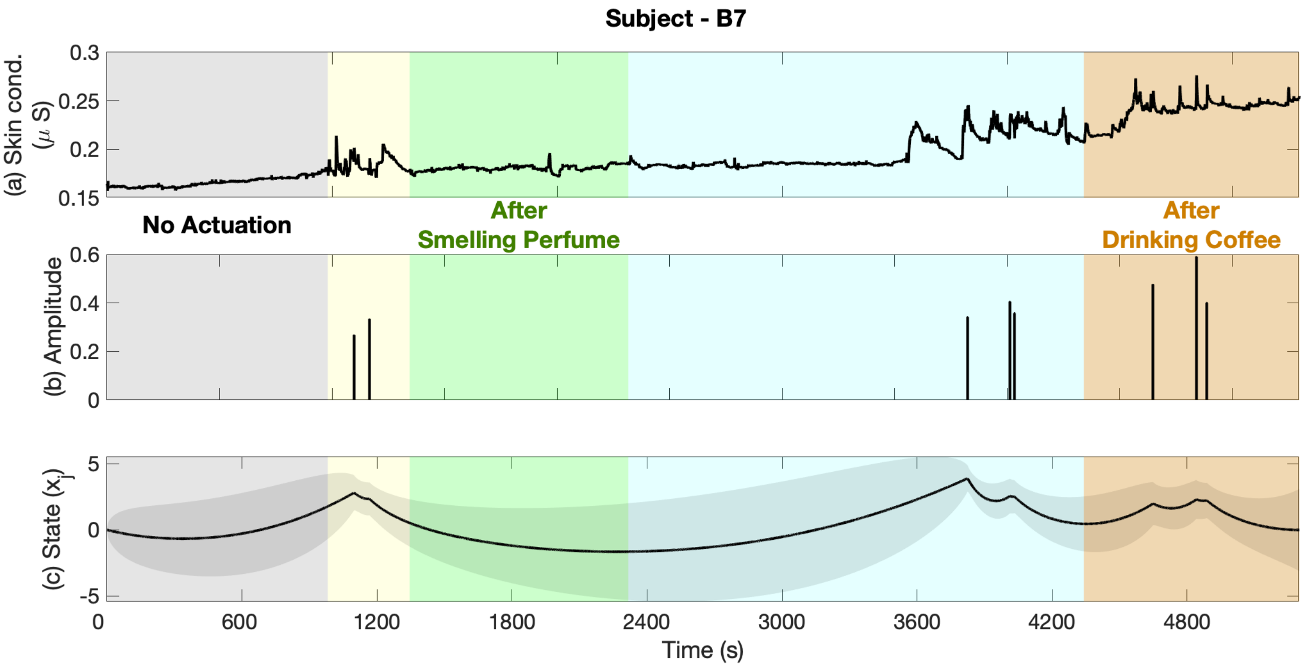

Figure S57. **Cognitive Arousal Results (Subject - B7, Experiment 2).** Panels from the top show in turn the skin conductance signal, underlying neural impulses, and estimated cognitive arousal state. The grey, green, and rust background colors represent the results associated with no actuation, smelling perfume, and drinking coffee sessions, respectively. Yellow and blue background colors are associated with rest time periods for smelling perfume and drinking coffee, respectively.

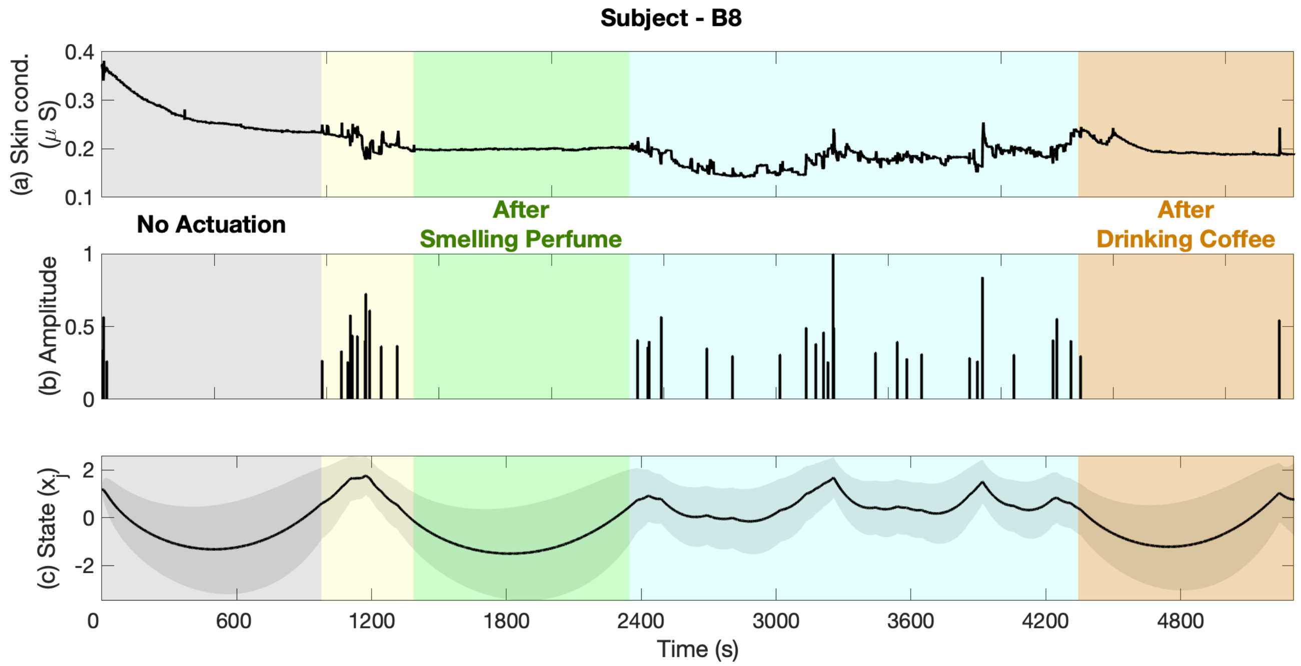

Figure S58. **Cognitive Arousal Results (Subject - B8, Experiment 2).** Panels from the top show in turn the skin conductance signal, underlying neural impulses, and estimated cognitive arousal state. The grey, green, and rust background colors represent the results associated with no actuation, smelling perfume, and drinking coffee sessions, respectively. Yellow and blue background colors are associated with rest time periods for smelling perfume and drinking coffee, respectively.

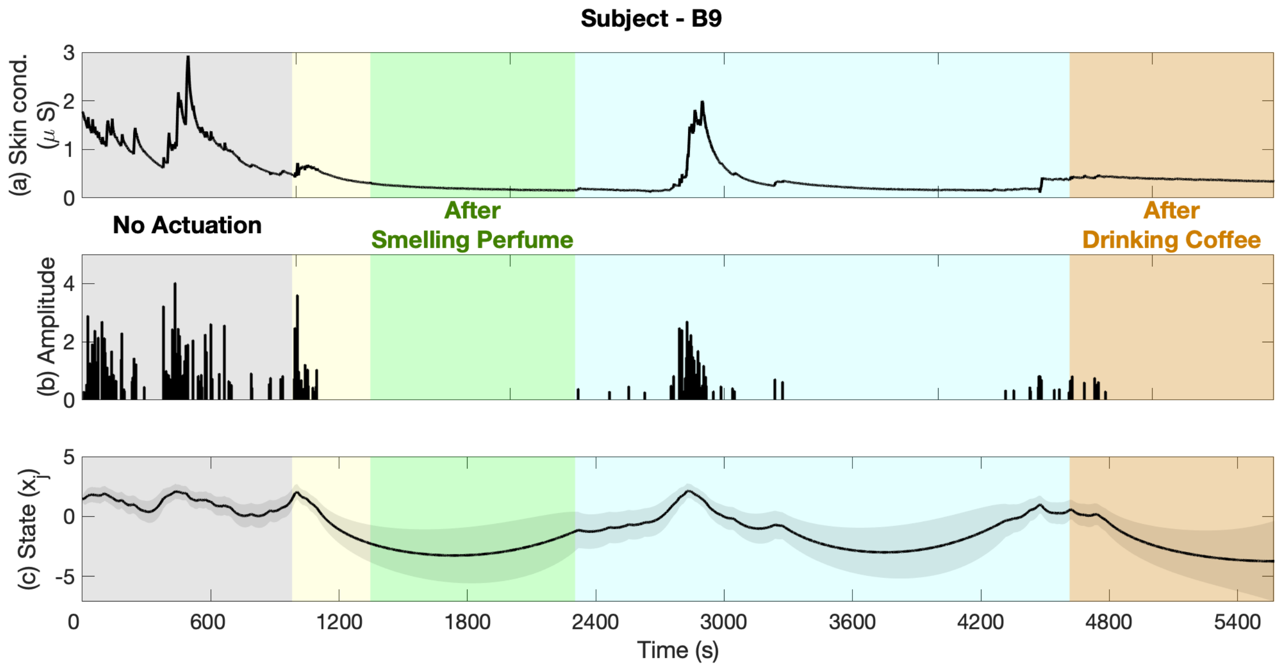

Figure S59. **Cognitive Arousal Results (Subject - B9, Experiment 2).** Panels from the top show in turn the skin conductance signal, underlying neural impulses, and estimated cognitive arousal state. The grey, green, and rust background colors represent the results associated with no actuation, smelling perfume, and drinking coffee sessions, respectively. Yellow and blue background colors are associated with rest time periods for smelling perfume and drinking coffee, respectively.

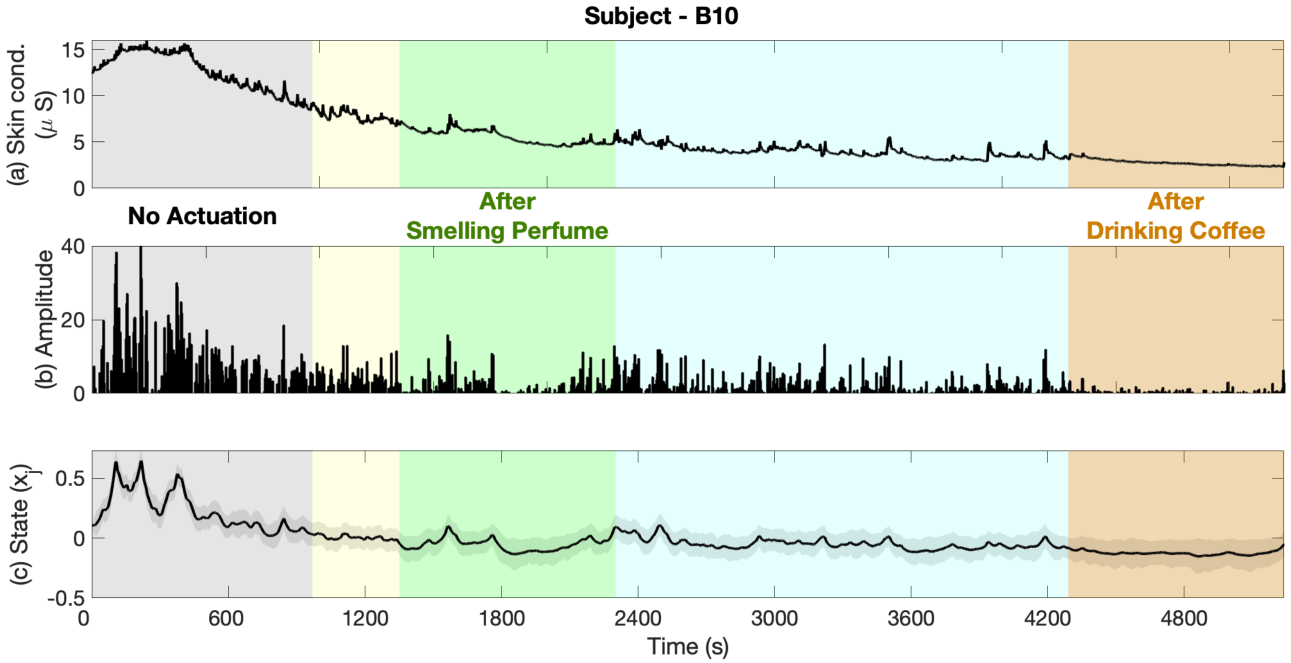

Figure S60. **Cognitive Arousal Results (Subject - B10, Experiment 2).** Panels from the top show in turn the skin conductance signal, underlying neural impulses, and estimated cognitive arousal state. The grey, green, and rust background colors represent the results associated with no actuation, smelling perfume, and drinking coffee sessions, respectively. Yellow and blue background colors are associated with rest time periods for smelling perfume and drinking coffee, respectively.

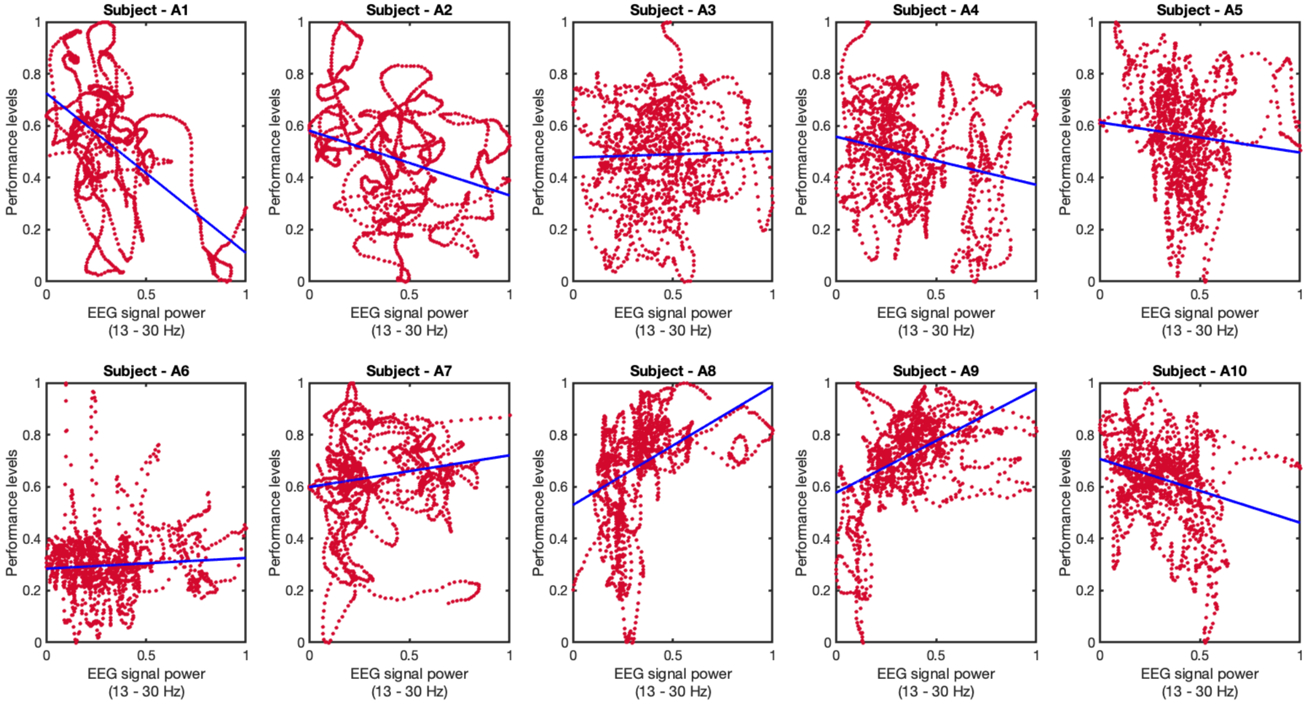

Figure S61. EEG Beta Band Power-Performance Relationship. Panels show the relationship between the averaged levels of beta band power in the EEG signal and the normalized levels of estimated cognitive performance state for all subjects in experiment 1. The blue line in each figure shows the fitted line to the actual data shown in red.

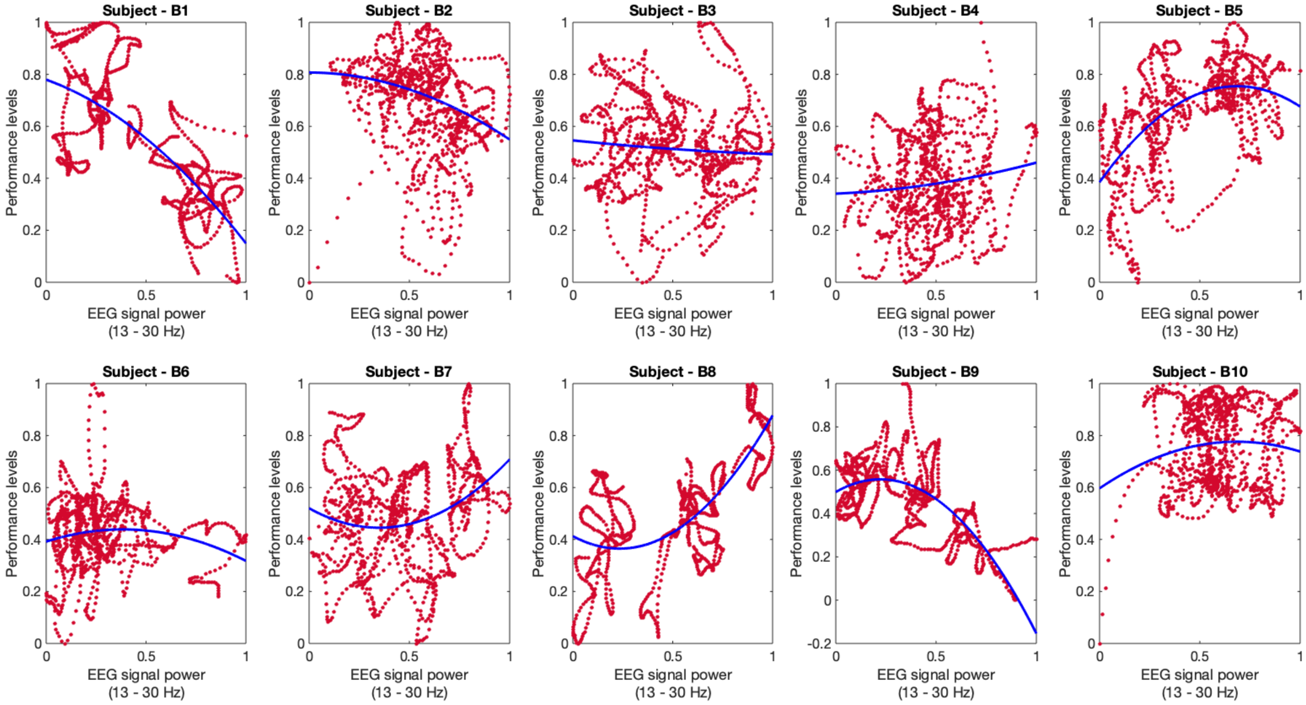

Figure S62. EEG Beta Band Power-Performance Relationship. Panels show the relationship between the averaged levels of beta band power in the EEG signal and the normalized levels of estimated cognitive performance state for all subjects in experiment 2. The blue curve in each figure shows the fitted curve to the actual data shown in red.

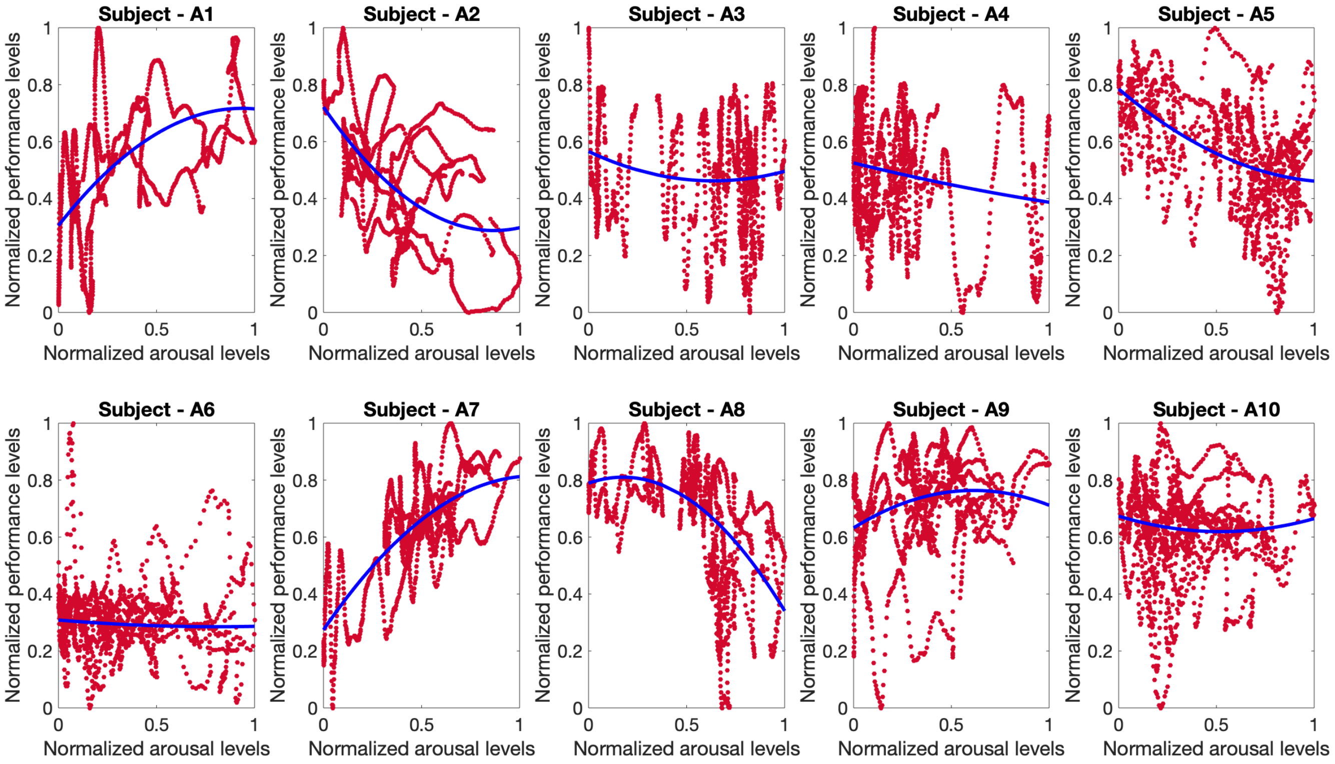

Figure S63. Arousal-Performance Relationship. Panels show the relationship between the normalized levels of estimated cognitive arousal and estimated cognitive performance state for all subjects in experiment 1. The blue curve in each figure shows the curve fitted to the actual data shown in red.

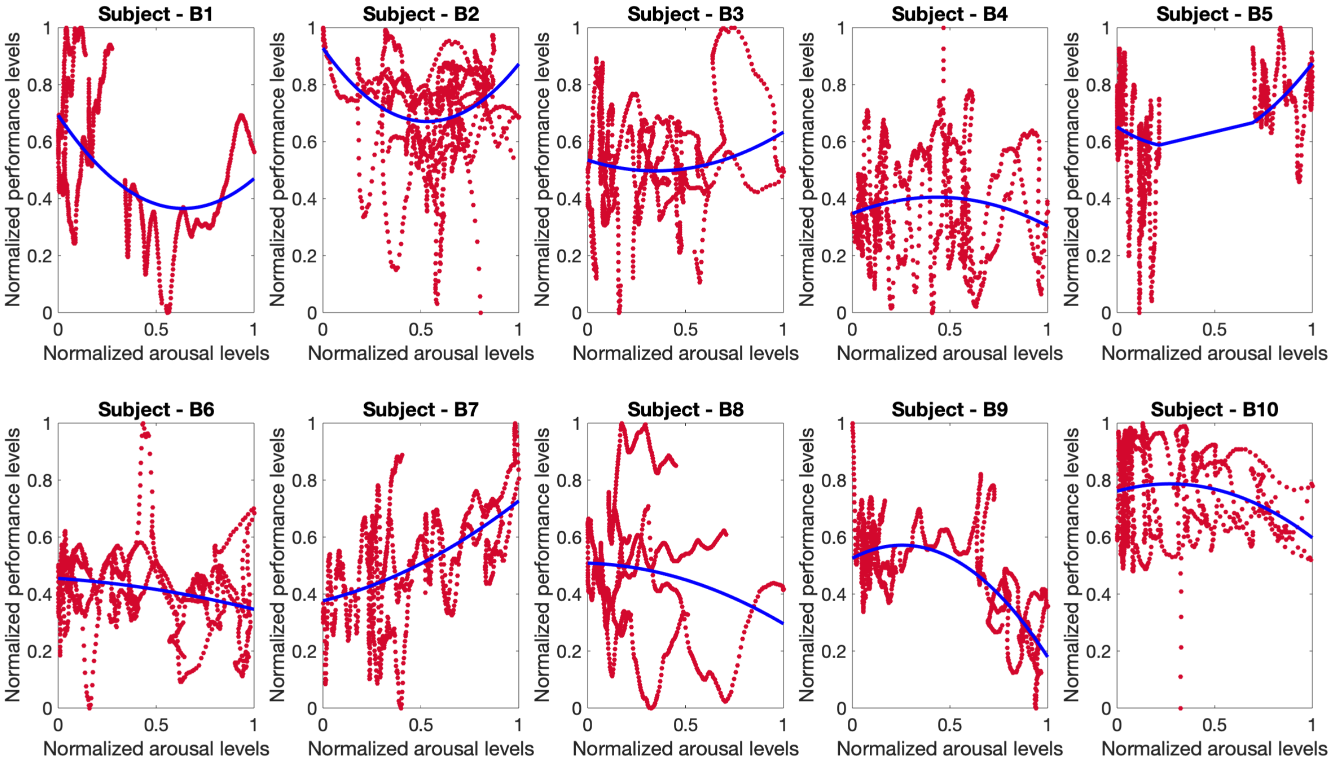

Figure S64. **Arousal-Performance Relationship.** Panels show the relationship between the normalized levels of estimated cognitive arousal and estimated cognitive performance state for all subjects in experiment 2. The blue curve in each figure shows the curve fitted to the actual data shown in red.
